# Advancements in antimicrobial nanoscale materials and self-assembling systems†

**DOI:** 10.1039/d1cs00915j

**Published:** 2022-10-03

**Authors:** Jack A. Doolan, George T. Williams, Kira L. F. Hilton, Rajas Chaudhari, John S. Fossey, Benjamin T. Goult, Jennifer R. Hiscock

**Affiliations:** School of Chemistry and Forensic Science, University of Kent Canterbury Kent CT2 7NH UK J.R.Hiscock@Kent.ac.uk; School of Biosciences, University of Kent Canterbury Kent CT2 7NJ UK B.T.Goult@Kent.ac.uk; School of Chemistry, University of Birmingham Edgbaston Birmingham B15 2TT UK G.T.Williams@Bham.ac.uk

## Abstract

Antimicrobial resistance is directly responsible for more deaths per year than either HIV/AIDS or malaria and is predicted to incur a cumulative societal financial burden of at least $100 trillion between 2014 and 2050. Already heralded as one of the greatest threats to human health, the onset of the coronavirus pandemic has accelerated the prevalence of antimicrobial resistant bacterial infections due to factors including increased global antibiotic/antimicrobial use. Thus an urgent need for novel therapeutics to combat what some have termed the ‘silent pandemic’ is evident. This review acts as a repository of research and an overview of the novel therapeutic strategies being developed to overcome antimicrobial resistance, with a focus on self-assembling systems and nanoscale materials. The fundamental mechanisms of action, as well as the key advantages and disadvantages of each system are discussed, and attention is drawn to key examples within each field. As a result, this review provides a guide to the further design and development of antimicrobial systems, and outlines the interdisciplinary techniques required to translate this fundamental research towards the clinic.

## Abstract 2

Antimicrobial resistance is directly responsible for more deaths per year than either HIV/AIDS or malaria and is predicted to incur a cumulative societal financial burden of at least $100 trillion between 2014 and 2050.

## Graphical abstract

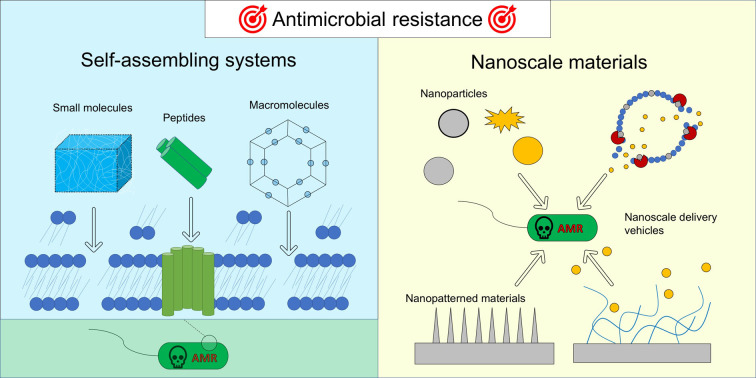

## 1. Introduction

The rise of antimicrobial resistant (AMR) bacterial infections is known to be one of the greatest threats to human health.^1^ Termed the ‘silent pandemic’ by some,^2^ from the year 2014 to 2050 AMR is predicted to have a cumulative financial burden of $100 trillion, and be directly responsible for 50 million deaths globally.^3^ However, this figure is not representative of the total indirect cost to human health; many other treatment regimens such as chemotherapy require the prophylactic co-administration of antibiotics due to increased infection risk,^4^ which will become harder to prescribe effectively as antimicrobial resistance spreads. Indeed, infection represents the second highest cause of death in cancer patients.^5^ Currently, AMR is responsible for more deaths per year than those attributed to HIV/AIDS or malaria.^6^ In addition, the recent COVID-19 pandemic has resulted in the increased use of antimicrobial agents, and further driven the rise of AMR.^7^

Since Fleming's discovery of penicillin in 1928,^8^ small molecule antibiotics have become a linchpin in the war against infectious disease. So followed the golden age of antimicrobial discovery/development. Between 1950 and 1960, half of all currently used antibiotics were isolated, with the rapid discovery of new agents continuing until the early 1990s.^9–11^ Today, the antimicrobial development pipeline has all but dried up as a result of the increasing prevalence of AMR, combined with poor market returns on investment for drug developers.^12^ In the last 20 years only two new classes of antibiotic have been developed, with a selective antimicrobial activity against the more susceptible Gram-positive over the harder to kill Gram-negative bacteria.^13^ Thus, novel antimicrobial development now lies within the remit of academic and small/medium-sized enterprises (SMEs).

The World Health Organisation (WHO) has produced a list of high priority pathogens, for which novel antimicrobial solutions are most urgently required.^14^ Perhaps the greatest cause of current concern is multidrug resistant tuberculosis (MD TB),^15^ however there are a number of other microbial species and strains considered a major threat to human health, owing to their broad spectrum resistance and subsequent lack of treatment options, see Table 1.^16^

**Table 1:** The priority list of bacteria for which novel antimicrobials are desperately needed, as published by the WHO^14^

Priority level	Bacteria
Critical	*Acinetobacter baumannii* – carbapenem resistant
	*Pseudomonas aeruginosa* – carbapenem resistant
	*Enterobacteriaceae* – carbapenem resistant, third generation cephalosporin resistant
High	*Enterococcus faecium* – vancomycin resistant
	*Staphylococcus aureus* – vancomycin resistant, methicillin resistant
	*Helicobacter pylori –* clarithromycin resistant
	*Campylobacter* spp. – fluoroquinolone resistant
	*Salmonella* spp. – fluoroquinolone resistant
	*Neisseria gonorrheae* – third generation cephalosporin resistant, fluoroquinolone resistant
Medium	*Streptococcus pneumoniae* – penicillin non-susceptible
	*Haemophilus influenzae* – ampicillin resistant
	*Shigella* spp. – fluoroquinolone resistant

When developing a new antimicrobial treatment and considering its translation into clinical practice, there is more to consider than simply an ability to kill proof-of-principle strains of planktonic bacteria. Bacterial infections, even from the same species, can differ massively depending on the patient, the site of infection, and the presence (or lack) of a biofilm.^17^ Biofilms are surface associated communities comprising of either single or multiple species,^18^ which gain the ability to act as a pseudo-multicellular organism, often resulting in increased levels of AMR.^18^ Indeed, the minimum biofilm inhibition concentration (MBIC) of an antibiotic towards certain bacteria can be up to a thousand-fold higher than the minimum inhibition concentration (MIC) of the same bacteria displaying a planktonic phenotype.^19^ Certain infections such as those associated with chronic wounds,^20^ diabetic foot ulcers^21^ and medical implants^22^ run a much higher risk of being associated with biofilms. In the case of diabetic ulcers/exposed wounds, treatments may be administered in high concentrations topically. However, delivering the high concentrations of antimicrobial needed to treat internal biofilms can be problematic,^23^ leading to persistent infections. These static communities of bacteria can be quiescent; in addition to possessing the capability of releasing planktonic cells which circulate, spreading disease.^18^ Often systemic antimicrobial treatments will eradicate these newly released bacteria, but not affect the biofilm, allowing it to persist.

As in all medicinal chemistry, the route of administration of an antimicrobial is a crucial consideration in its development. The topical application of antimicrobial agents in the form of creams, gels, liquids and sprays, is common in the treatment of surface wounds and skin infection, whilst systemic treatments are generally administered orally or intravenously. It is conventionally the preference that drugs be administered orally, as this can be performed by untrained personal and there are fewer patient compliance issues.^24^ However, in severe infections such as sepsis, the increased speed with which IV antibiotics act systemically can mean the difference between life and death.^25^

The multitude of mechanisms by which bacteria may gain resistance to traditional small molecule antimicrobials only serves to add to the complexity associated with combatting AMR. One such mechanism is bacterial evolution altering the structure of antimicrobial targets, thus rendering the antimicrobial ineffective.^26^ A resistance mechanism of *Staphylococcus aureus* (*S. aureus*) to β-lactam antibiotics (*i.e.* penicillin) offers a classic example of such a strategy. To elicit antimicrobial action, the β-lactams bind to the penicillin binding protein, a key enzyme in cell wall synthesis.^27^ Certain strains of *S. aureus* have now evolved to instead express penicillin binding protein 2a (PBP2A).^28^ This displays a much lower affinity for β-lactam antibiotics, preventing their activity and conferring these strains of bacteria with resistance.^28^ As well as alteration of bacterial target sites, many microbes have also evolved to produce enzymes that inactivate certain therapies.^29^ These include but are not limited to: the production of β-lactamases to deactivate β-lactam antibiotics,^30^ aminoglycoside modifying enzymes to confer resistance to aminoglycoside antibiotics^31^ and esterase mediated hydrolysis of macrolide antibiotics.^32^ Increased expression of efflux pumps, protein channels capable of removing drug molecules (as well as organic pollutants and other biocides) from within the cell, is another common resistance mechanism, often conferring multidrug resistance to bacteria.^33,34^ Whilst the drug is still able to cross the cellular membrane and enter the bacteria, subsequent ejection of these molecules reduces the effective concentration to below the therapeutic threshold of the drug.^33,34^ A conceptually more primitive mechanism of antimicrobial resistance is that of bacterial cell wall thickening; this thickening has been shown to be responsible for vancomycin resistance in *S. aureus*, thought to be due to entrapment of the drug within the cell wall preventing efficient cellular uptake of the antibiotic.^35^

Small molecule biocidal agents that target cell membranes are already used extensively in clinical care,^36^*e.g.* chlorhexidine, used in topical treatments such as antimicrobial mouthwash formulations.^37^ Such agents are generally cationic, relying on the difference in cellular surface charge to ensure selectivity for bacterial over mammalian cells.^38^ Thus, bacterial modification of surface charge, through alteration of their phospholipid compositions, offers an avenue for resistance to these antimicrobials.^39,40^ Despite this, this class of antimicrobial agents are less affected by antibiotic inactivating enzymes and the over-expression of efflux pumps, indicating a reduced propensity for the generation of resistance. The use of membrane active nano-structures that are not reliant on this difference in charge offer a potential avenue for the development of antimicrobial treatments for which bacteria can less readily develop resistance. Furthermore, by developing complex nanostructures comprised of multiple independent small molecules, a high effective concentration of the active agent can be delivered to the bacteria, despite a low systemic concentration, effectively killing bacteria to treat the resultant infection.

Eliminating the threat of AMR is multifaceted, and will require a paradigm shift in the way antimicrobials are considered in both the societal and economic sense.^3^ Perhaps the most obvious solution to AMR is in the development of novel traditional small molecule antibiotics.^41,42^ However, many researchers are pursuing alternative antimicrobial innovations. These are varied and span the full range of the physical and life sciences, and include the use of drug delivery systems,^43^ atmospheric plasmas,^44,45^ bacteriophage,^46^ probiotics^47^ and even the use of CRISPR.^48^ This review aims to act as a repository for recent antimicrobial innovations, bringing together the work of those developing novel antimicrobial therapies, with a focus on self-assembling systems, nanoparticles, nanopatterned materials and nanoscale drug delivery systems.

### 1.1. Self-assembly

Much of the research outlined in this review utilises the concept of self-assembly and supramolecular interactions. These supramolecular interactions include a range of intermolecular non-covalent bonds. Unlike the majority of covalent bonds, these interactions are reversible and include: electrostatic van der Waals forces (<5 kJ mol^−1^);^49^ dipole–dipole interactions (5–50 kJ mol^−1^);^50^ π–π interactions (5–40 kJ mol^−1^);^51^ hydrogen bonding (4–165 kJ mol^−1^);^50^ ion–dipole interactions (50–200 kJ mol^−1^)^52^ and ion–ion interactions (100–350 kJ mol^−1^)^53^ Beyond these forces, supramolecular assembly may also be driven by hydrophilic/hydrophobic interactions, as is often the case in nature.^54^ Self-assembly and supramolecular interactions are crucial in the interactions and folding of biomolecules such as proteins,^55^ as well as underpinning the stability of DNA.^56^

### 1.2. Practical considerations for complex antimicrobial systems

Regardless of the amount of therapeutic agent that is administered to a patient, only the amount of agent that reaches the disease site can contribute towards the treatment of the disease; this amount is known as the effective concentration. Drugs are often formulated to improve this effective concentration, for example to avoid degradation in the gastrointestinal tract, or to increase uptake across mucosal membranes.^57^ This complex problem is only further complicated by the consideration of self-assembling systems, which by their very nature are targeted to interact with other molecules. A multitude of the examples explored within this review rely on utilising the cationic charge associated with an aggregated or monomeric antimicrobial species to enable the selective targeting of anionic bacterial membranes, however optimising these interactions is known to result in off-site binding with serum proteins *in vivo*.^58^ Whilst strategies such as co-formulation with poly(ethylyne glycol) and the use of carriers have been explored,^59–61^ it is clear that the administration method and subsequent formulation of these self-assembling systems is of the utmost importance when considering the treatment of infection *in vivo*, and will often require specific molecular/formulation design optimisation on a case by case basis. This places another barrier to the translation of these technologies into the clinic, as it is paramount that the characteristics of these formulations/carriers be considered, for example size, size distribution, degree of charge and shape, as each of these factors can affect biodistribution and accumulation. Each of these factors may act independently or interdependently, presenting potential roadblocks to translation.^62^ Such barriers are less of a problem with topically applied therapies, for which organ accumulation and eventual excretion are not an issue.

As well as the method by which these antimicrobial therapies are administered to a patient, the practical consideration of their pre-administration sterilisation must also be considered. Traditional sterilisation methods including pasteurisation with heat and pressure (often performed in an autoclave), sterilisation using gamma radiation and filter sterilisation, typically performed using 0.22 μM filters, are often inappropriate for use with certain synthetic (heating or radiation) or self-associated (filtration) species.^63^ Nanomedicine is a comparatively new field when compared to classical pharmacology,^64^ and as such is still currently treated similar to conventional chemicals. As a consequence, scientists and regulatory bodies have made efforts to devise a set of unifying procedures for establishing the safety of nanomaterials.^65,66^ Wilhelm and co-workers have produced an in-depth report outlining potential nanomaterial toxicities,^67^ concluding the requirement for an assessment of these therapies on a case-by-case basis, with the end fate of these materials varying dependent on a range of physical and chemical parameters.^67^ The complexity of such systems combined with that of an *in vivo* environment makes predicting their toxicological effects incredibly difficult to achieve.

## 2. Self-assembling peptides as antimicrobial agents

Exploiting the antimicrobial activity of naturally derived peptides has been ongoing since 1939, when gramicidin, the first isolated antimicrobial peptide (AMP), was isolated from *Bacillus brevis.*^68^ Gramicidin was subsequently shown to be effective in the treatment of infected wounds and ulcers.^69^ This discovery kick started a pursuit to harness natural AMPs, which act as a first line of defence against invading pathogens within the innate immune system of humans.^70,71^ To date, a diverse array of AMPs have been identified in mammals,^72^ amphibians,^73^ fungi^74^ and invertebrates,^75–77^ prompting the publication of a number of review articles.^78–80^ Building on these discoveries, the development and investigation of short synthetic self-assembling AMPs and their application as antimicrobials has become increasingly popular, offering multiple benefits over their naturally derived counterparts. The importance of this class of antimicrobials is evidenced by the potential translation of this technology into the clinic *e.g.* as of 2019, 27 AMPs were in clinical trials.^80,81^

### 2.1. Self-assembling AMPs

**Fig. 1:**
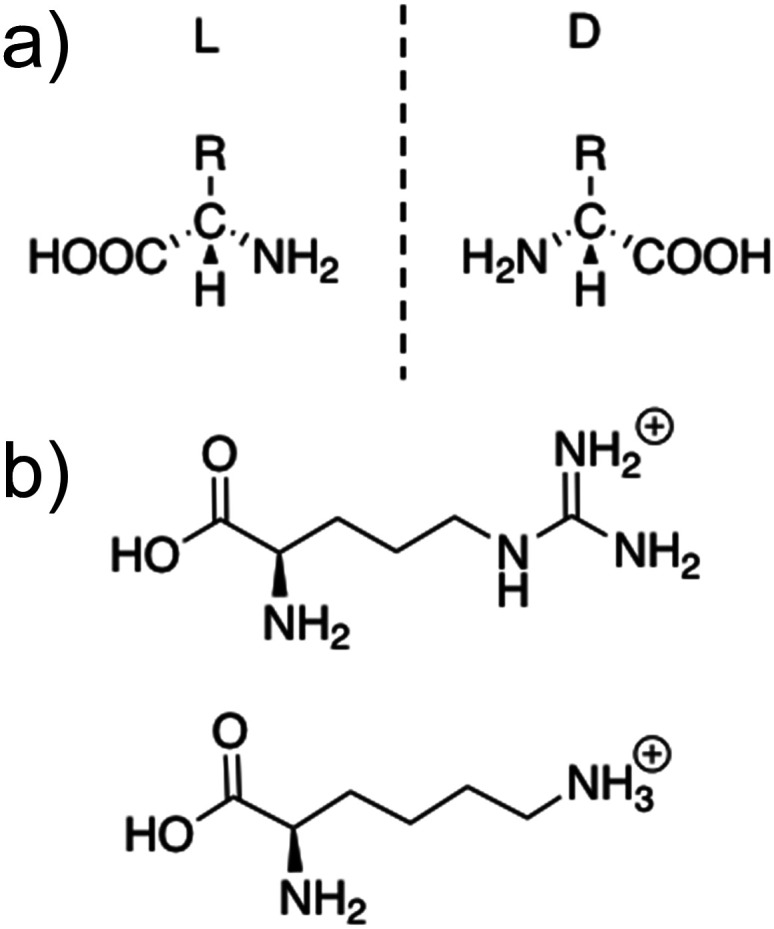
(a) The general structure of l- and d-amino acids. (b) The structures of positively charged l-arginine and l-lysine amino acids.

Naturally occurring AMPs are produced from the library of 20 natural l-amino acids, all of which are inherently chiral with the exception of glycine.^82^ The generic structure of both l- and d-amino acids is shown in Fig. 1a. AMPs are typically between 10–60 amino acids in length, with each of the amino acids contained within the peptide sequence contributing to the overall physiochemical properties of the AMP.^83^ For instance, arginine's side chain (shown in Fig. 1b) contains a guanidinium group, which acts as a base.^84^ At physiological pH (7.4), the arginine group is positively charged due to protonation of the guanidium, a property that contributes to an enhancement in AMP water solubility.^85^ The presence of positively charged amino acids such as lysine and arginine, often localised to specific regions within an AMP sequence, commonly causes this class of compound to present a net positive charge of between +2 to +13.^71,77,86,87^ Additionally, the R-groups of the different amino acids present within the AMP can form non-covalent bonds both with other amino acids within the AMP to form a secondary structure and between neighbouring AMPs to form higher order structures. These non-covalent bonds include: hydrogen bonds, van der Waals, π–π stacking, hydrophobic and electrostatic interactions.^88^

**Fig. 2:**
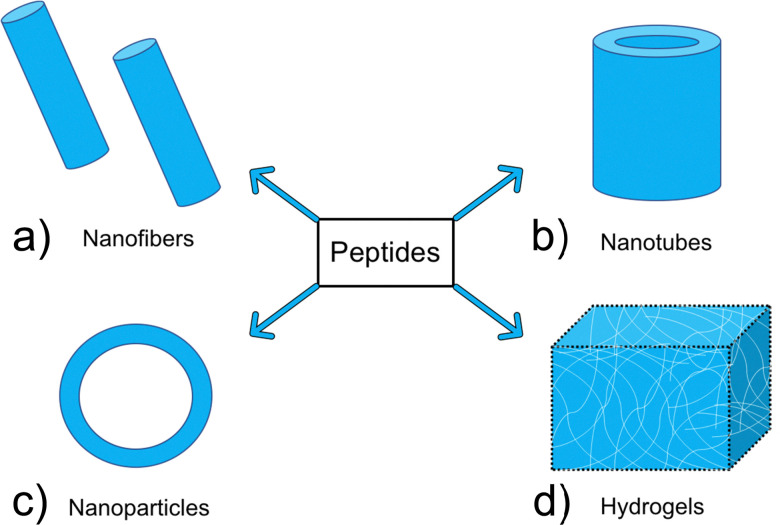
Examples of common nanostructures formed *via* AMP self-assembly: (a) nanofibers (b) nanotubes (c) nanoparticles (d) hydrogels.

Many of the self-assembling AMPs found in animals^89–92^ when extracted, initially fail to self-assemble in saline or buffer solutions at physiological pH, instead achieving assembly only when in proximity to negatively charged phospholipids within biological membranes, or in solutions with a high pH due to neutralization of charged groups within the AMP.^93,94^ Under the conditions required for self-assembly, short AMP sequences initially self-assemble into random coil structures, transitioning into α-helices, β-sheets and β-turns over time *via* increased numbers of hydrogen bonds present between the amino acids within the AMP.^95^ Following successful secondary structure formation, more complex nanostructures spontaneously self-assemble through interactions with neighbouring AMPs. The form of these larger nanostructures is dependent on the individual AMPs ability to form intermolecular bonds. In the next section we describe some of the more common nanostructures produced (Fig. 2).

#### 2.1.1. Nanofibers and nanotubes

AMP derived nanofibers, such as those shown in Fig. 2a typically have a diameter of less than 100 nm and can be formed in aqueous solutions, a key requirement for clinical applications.^96^ Of the various AMP building blocks, peptide amphiphiles terminated with an alkyl group side chain are amongst the most common to form these AMP nanofiber structures.^96^ AMP derived nanotubes, shown in Fig. 2b, differ to AMP derived nanofibers due to their hollow core, as opposed to the solid interior seen with AMP derived nanofibers. The hollow core of the AMP nanotubes enables application within the field of drug delivery, whereby encapsulation of active compounds within the core allows for transfer to target sites, with controlled release increasing the efficacy of the therapeutic payload.^97^ Cyclic peptides are the most studied building blocks for formation of AMP nanotubes; with a minor number of studies showing non-cyclic peptides that are also capable of forming AMP nanotubes.^98–100^ Both AMP nanofibers and AMP nanotubes present advantageous characteristics such as high aspect ratio. This property has been demonstrated to increase both intracellular uptake and the time a therapeutic agent may be retained in the blood stream, which is known to increase therapeutic efficacy.^101^

#### 2.1.2. Nanoparticles (NPs)

AMP derived nanoparticles (NPs), shown in Fig. 2c are defined as particles under 100 nm in size in at least one dimension.^102^ These AMP NPs include a range of different structures including micelles^103^ and solid particles.^104^ Amphiphilic oligopeptides, cyclic peptides and a range of other building blocks have been demonstrated to form AMP NPs.^83^ Similar to other nanomaterials, NPs also offer high aspect ratios, instilling multiple beneficial pharmacokinetic properties.^101,105^

#### 2.1.3. Hydrogels

Hydrogels (Fig. 2d) have attracted interest from researchers across a broad range of disciplines. These are crosslinked or entangled polymer networks which form 3D matrixes with a high water content.^106^ Hydrogels may be formed from a range of synthetic (*i.e.* poly(vinyl alcohol)^107^) polymers, or natural polymers such as polypeptides.^108^ Through alteration of the crosslinking methodology or chemical alteration of the polymer backbone, the mechanical and chemical properties of AMP based hydrogels can be tailored, and subsequent responsive functionality can be achieved.^109,110^ Using relatively simple peptide sequences, an expansive range of hydrogels have been produced using both amphiphilic AMPs^111^ and cyclic peptides.^112^

### 2.2. AMPs mechanisms of action

**Fig. 3:**
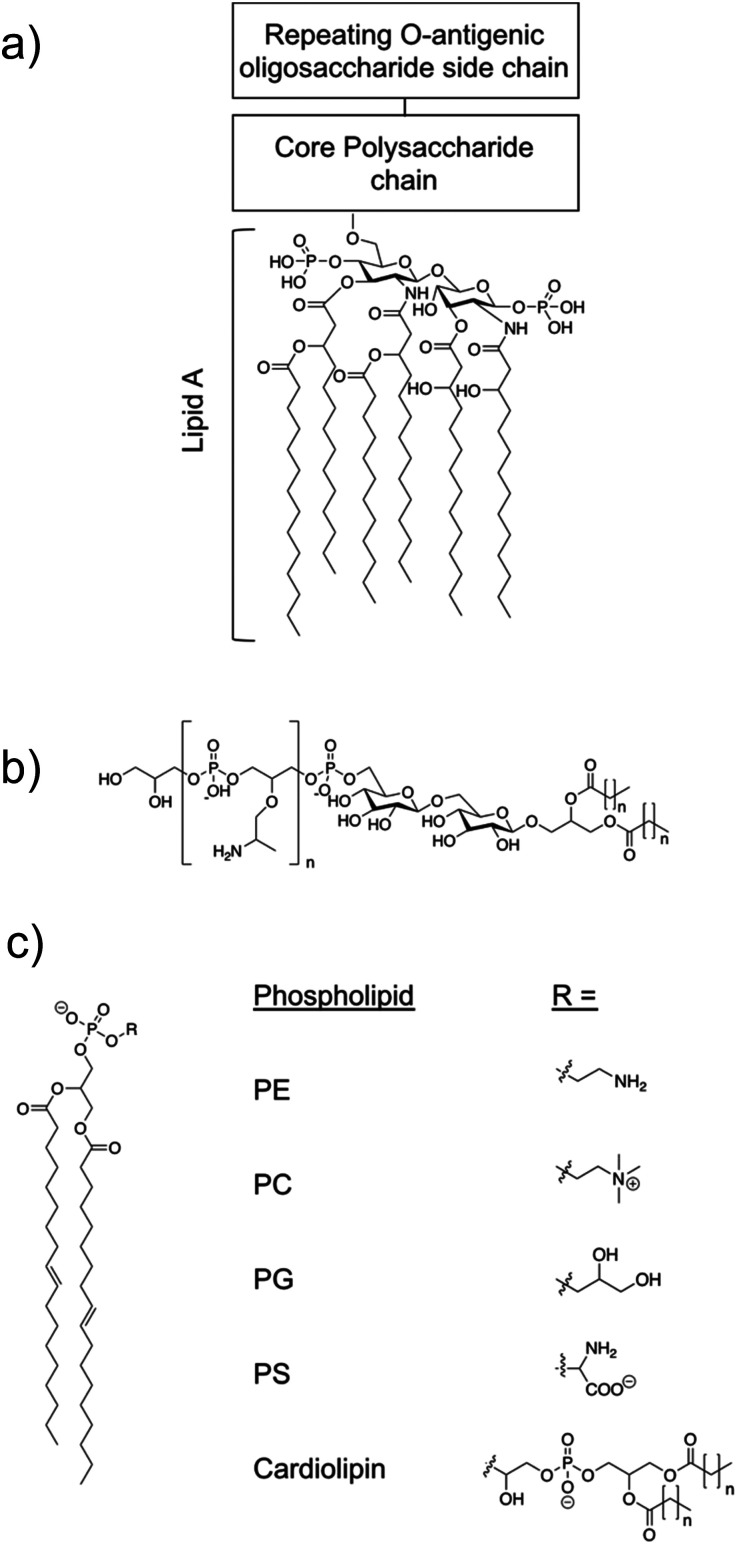
(a) The structural components of LPS.^119^ Molecular composition is known to vary between bacterial species. (b) Lipoteichoic acid structure.^115^ (c) An example of a phospholipid structure^120^ and the molecular structure of different phospholipid headgroups phosphatidylethanolamine (PE), phosphatidylcholine (PC), phosphatidylglycerol (PG), phosphatidylserine (PS) and cardiolipin.^115^

The overall cationic charge of AMPs results in adhesion, *via* complementary electrostatic interactions, to negatively charged cell surfaces, such as those of bacteria. Negative charge is bestowed on Gram-positive and Gram-negative bacterial membranes due to a significant percentage of negatively charged lipids contained within the outer leaflet of the cell surface membranes.^113^ These negatively charged lipids include phosphatidylglycerol (PG) and cardiolipin (Fig. 3c), which vary in abundance across bacterial species.^114,115^ Additionally, Gram-positive bacteria contain the anionic lipoteichoic acid (Fig. 3b) and Gram-negative bacteria contain the anionic liposaccharide (LPS) (Fig. 3a), further enhancing the overall negative charge at the cell surface.^116^ In contrast to bacterial cells, healthy mammalian cells have an overall net neutral surface charge due to the zwitterionic phosphatidylcholine (PC) (Fig. 3c) and phosphatidylethanolamine (PE) (Fig. 3c) presenting as the predominant lipids at the surface of the cells.^115,117,118^

These differences in the surface charge between mammalian cells and bacterial cells enable specific cellular interactions of AMPs with bacteria.^121,122^ When investigating the discussed specificity of an AMPs targeting to bacterial cells, mammalian red blood cells (RBCs) are frequently used as the control cell line, due to haemolysis presenting as a common side effect from treatment with AMPs.^122^ The outer membrane of mammalian RBCs is primarily composed of the zwitterionic lipids PC and sphingomyelin, bestowing them with the overall neutral membrane charge as previously stated.^123,124^ Human RBCs also contain approximately 10% negatively charged PS, however this is mostly contained within the inner leaflet of the outer cell membrane.^124,125^ Together, these differences in membrane composition enable the design of AMPs that specifically target bacterial membranes.

**Fig. 4:**
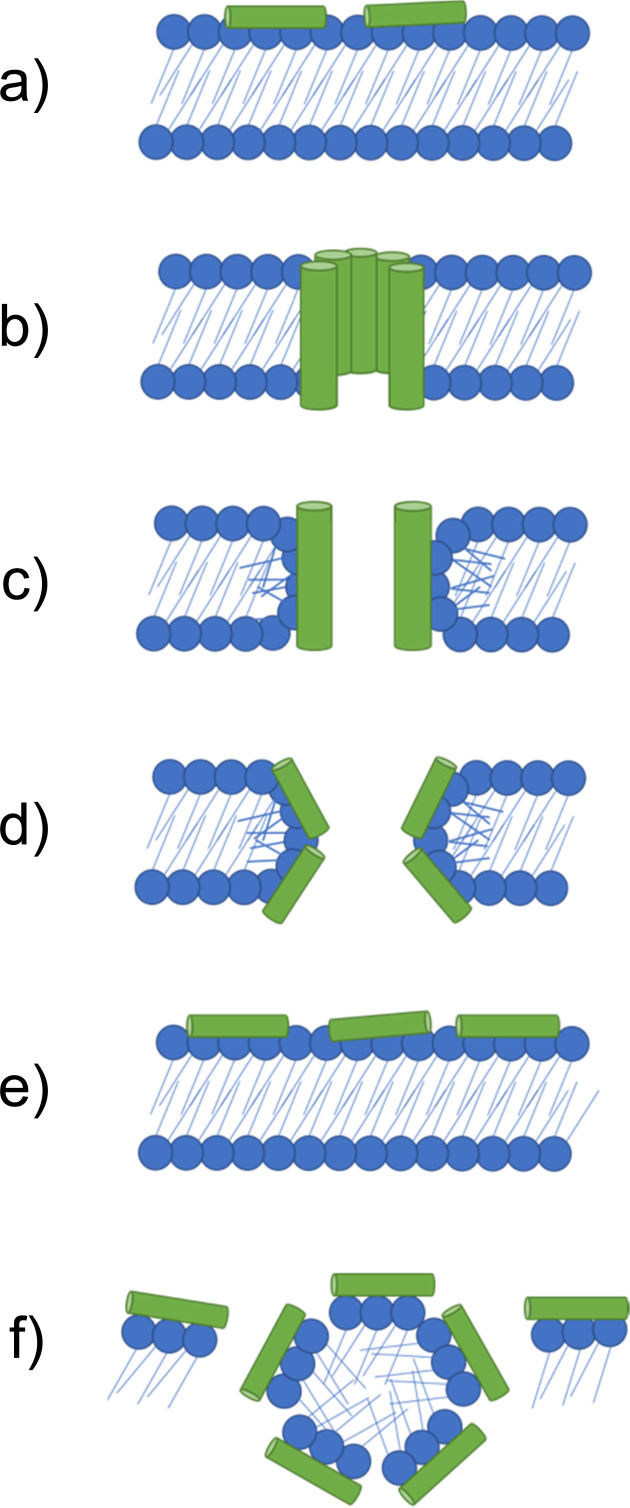
Models by which AMPs elicit antimicrobial activity. (a) If not assembled prior to arriving at the membrane, peptides initially accumulate at the bacterial membrane in a parallel orientation. (b) In the barrel-stave model a pore is formed with peptides in a transmembrane orientation. Hydrophobic amino acids face the lipid membrane and hydrophilic residues line the lumen (the inside of the pore). (c) In the traditional toroidal pore model, peptides bend lipids into the pore; this forms a continuous pore lined by both AMPs and lipid head groups. (d) Disordered toroidal pores are also lined by lipids, however, contain only one or two peptides lining the pore, often located at the edges of the self-assembled structure produced. (e) In the carpet model, AMPs continue to coat the membrane in a parallel formation, forming an extensive layer referred to as a carpet. (f) At high concentrations the carpet can cause disruption *via* detergent action.^133^

If higher order structures are not assembled in the solution state before arriving at the membrane, the initial electrostatic adhesion of AMPs to a negatively charged cell surface results in parallel alignment to the membrane.^126^ With increasing concentrations of AMPs accumulating at the negatively charged membrane surface, molecular self-association promotes the formation of higher order structures. Alternatively, Petkov *et al.* showed some AMPs are able to self-assemble in solution, arriving at the membrane in the folded form required for membrane insertion.^127^ When the critical AMP concentration is reached at the cell surface, AMPs elicit antimicrobial action through destabilisation of the cell membrane, leading to molecular permeabilization, leakage of internal cell contents and eventually cell death.^128,129^ The method by which AMPs achieve membrane permeation after the critical molecular concentration is reached at the cell surface are often prescribed to one of the following models: the barrel-stave, toroidal pore, carpet and detergent models,^87^ each of which are illustrated in Fig. 4. The properties of each AMP dictate the model through which membrane disruption is achieved.

#### 2.2.1. The barrel-stave and toroidal pore models

When the critical concentration of AMPs at the cell surface is reached, asymmetry created by the peptides bound to the surface of the bilayer result in a thermodynamically unstable membrane.^130^ In the barrel-stave and toroidal pore models, this thermodynamic instability results in the peptides distributing between the monolayer leaflets of the membrane in a perpendicular orientation, inserting into the hydrophobic centre,^117^ leading to membrane permeation.^131,132^

The barrel-stave model requires AMPs with specific amphipathic structures such as α-helices. These AMPs can form a bundle in the membrane with a central lumen (Fig. 4b).^131^ The amphiphilic structure is required due to the orientation of hydrophobic and hydrophilic residues with respect to each other. Specifically in α-helices, due to turns within the structure, hydrophilic residues can present on one face, while hydrophobic residues can align on the other.^134^ In barrel-staves, the hydrophobic residues align with the lipid membrane, while the hydrophilic residues line the lumen, creating a polar channel.^135^ Toroidal pores are formed when AMPs introduce local defects in the membrane. These defects allow peptides to bend lipid molecules into a pore, resulting in a pore lined with lipid head groups (Fig. 4c and d).^136^ Such toroidal pores can be classed as traditional or disordered, with their classification depending on the AMPs positioning within the pore.^137^ Traditional toroidal pores display transmembrane orientation of AMPs after inward bending of lipid molecules, allowing for AMPs to be positioned throughout the entire lipid lined lumen (Fig. 4c). Disordered toroidal pores only contain one or two AMPs lining the pore, with most AMPs binding the edges of the pores (Fig. 4d).^130^ Melittin, the major component of bee sting venom, is one such AMP that acts *via* this toroidal pore mechanism.^89^ Following disruption of a membrane *via* melittin, elongation of the pore causes lipids to be sequestered into the lumen, forming a continuous pore lined by membrane lipids.^130,131^ This membrane distortion results in local bending of the membrane, ultimately leading to membrane disintegration.^138^

#### 2.2.2. The carpet and detergent models

The carpet model similarly begins with AMPs that electrostatically associate with the anionic lipid headgroups, lining the membrane in a parallel orientation (Fig. 4a), resulting in a ‘carpet’ of peptides on the surface of the membrane (Fig. 4c). Through continued accumulation of peptides at the membrane, the resulting thermodynamic instability results in a detergent like disintegration of the membrane (Fig. 4f),^130^ rather than perpendicular insertion into the membrane, with additional toroidal transient holes also being formed in the membrane.^131,139^

### 2.3. Properties of AMPs associated with antimicrobial activity

The importance of the cationic charge for the mechanism of action of AMPs is exemplified by the discovered correlation between increasing positive charge and resulting increased AMP antimicrobial activity.^117,140^ This is likely due to the greater positive charge increasing the probability and strength of interaction of AMPs with the membrane resulting in higher effective concentrations, ensuring that the critical concentration for membrane disruption is being met.^141^ In addition to charge, several other factors contributing to the antimicrobial efficacy of AMPs have been identified, such as hydrophobicity. This is defined by the percentage of hydrophobic residues in the peptide (*i.e.* valine, leucine, phenylalanine and tryptophan), typically falling between 40–60% of the total amino acid residues constituting the AMP.^142,143^ Hydrophobicity is implicated in the bacterial membrane partitioning observed from AMPs, suggesting a potential reason for the observed changes in antimicrobial activity upon structural modifications leading to increased levels of molecular hydrophobicity.^142^ Alongside enhanced antimicrobial activity, high levels of hydrophobicity have also been correlated with toxicity, demonstrating the importance of careful design considerations to achieve optimal efficacy balanced with minimal cytotoxicity.^144^ Amphipathicity, also implicated in antimicrobial efficacy, is a key structural property shared by all AMPs, either through their primary or secondary sequence, or in the higher order structure formed.^87^ Amphipathic molecules contain both hydrophilic and hydrophobic portions within their chemical structure, thus AMPs act as amphiphiles either by the design of their sequence or through formation of secondary structure, creating regions with a net hydrophilic or hydrophobic propensity.^145^ Amphipathic α-helices present a common example by which peptides display as amphiphiles through secondary structure,^87^ as are β-pleated sheet and extended/flexible peptide structures.^146^

### 2.4. Advantages and disadvantages of AMPs

Antibiotics commonly target a specific bacterial function and may be rendered ineffective by the evolution of resistance mechanisms.^138^ AMPs instead target the bacterial membrane through electrostatic interactions. Resistance to AMPs therefore presents a challenge for bacteria, requiring the microorganism to balance membrane function and structural integrity against modifications to evade AMP association.^147^ Additionally, as a result of membrane targeting, AMPs often exhibit broad spectrum antimicrobial efficacy, due to the net negative surface charge common to the outer membranes of most bacteria.^148,149^ Together, these benefits, in combination with chemical versatility, tuneability in hydrophilic and amphiphilic properties, biodegradability, enhanced biocompatibility and variable immunogenicity, present a strong argument for AMPs as potential antimicrobial agents, should researchers be able to overcome the limitations identified within the scope of this review.^150–152^

Specifically, high levels of haemolytic activity are often a key issue when considering the development of AMPs as antimicrobial agents.^71^ Initial failure to address these issues are exacerbated by use of non-mammalian or non-human RBCs during screening.^122^ Greco *et al.* showed large differences between EC_50_ values of AMPs against RBSs of ruminants (cows, camels, sheep) compared to other mammals (dogs, monkeys, horses), highlighting the importance of careful experimental design when probing AMP cytotoxicity.^122^ AMP biological stability is another key issue associated with the translation of this technology into the clinic. AMPs are often subject to high levels of enzymatic degradation and exhibit poor penetration of the intestinal lining, meaning this class of compound are of limited use as oral therapeutics.^81^ Introduction to a biological system *via* direct intravenous access is also problematic due to the short half-lives associated with AMPs, again as a consequence of enzymatic degradation.^81^ Furthermore, this class of compounds remain costly to produce, limiting commercial potential.^153^ In addition, the transport and distribution of many biological therapies requires what is termed the ‘cold-chain’, that is they must remain refrigerated or frozen during each step of the physical delivery process, from the manufacturing location to the clinical administration site.^154^ This can be particularly problematic when considering restrictions associated with therapeutic distribution in low- and middle-income countries, as highlighted by the recent and on-going distribution of vaccines for COVID-19.^155^ Finally, storage and shelf life are also a major consideration for all therapeutic agents developed. AMPs used in clinic (*i.e.* colistin and polymyxin B) can be stored long-term without considerable reduction in activity, however rationally designing AMPs that are stable remains a critical design consideration.

### 2.5. Dipeptides – simple AMPs inspired by nature

**Fig. 5:**
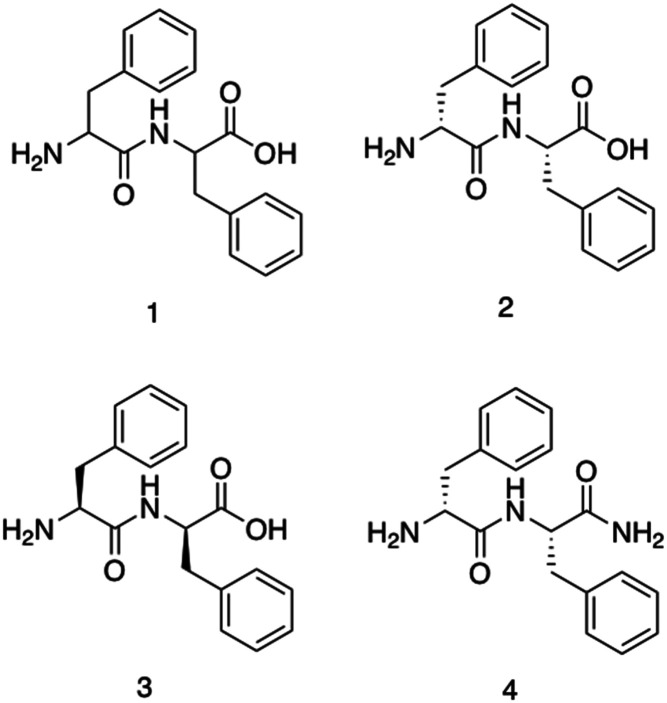
The structure of compounds 1–4 as developed by Schnaider *et al.*^156^ and Porter *et al.*^158^

Diphenylalanine, 1, is the shortest antimicrobial peptide agent reported to date, (Fig. 5).^156^ This compound is able to self-associate in solution through hydrogen bonding and π-stacking between neighbouring phenyl groups. These self-associative interactions lead to the formation of secondary β-sheet structures which are capable of further assembly into nanotubes, (Fig. 2b) spherical vesicles, nanowires and nanofibrils.^157^

Schnaider *et al.*^156^ developed the antimicrobial self-assembling peptide, 2, shown in Fig. 5. This work was inspired by Kumar *et al.*^159^ identifying the protective action of β-amyloid polypeptides against microbial infections. Attempting to understand the physiochemical properties underpinning the findings from Kumar *et al.*, Schnaider *et al.* focused on 1, the central recognition module of the β-amyloid polypeptide. Compound 1 has been identified as critical in the self-assembly of β-amyloid polypeptide, providing a logical avenue of investigation for antimicrobial action. Self-assembled unbranched nanotubes were produced by heat treating lyophilised powders of 2, followed by cooling overnight. The self-associated nanotubes of 2 displayed activity against *Escherichia coli* (*E. coli*) (ATCC 25922), achieving a MIC of 125 μg mL^−1^. The MIC refers to the concentration required to prevent visible growth of bacteria. Scanning electron microscopy (SEM) images revealed cells treated with 2 to contain ‘nicks’ and ‘tears’ in the membrane, with membrane clumping and disintegration also visible. Using the fluorescent dye 8-anilino-1-naphthalenesulfonic acid (ANS), the permeation of the membrane in response to compound 2 was confirmed through observed increases in fluorescence upon treatment with 2 compared to the controls. ANS displays increased fluorescence within hydrophobic environments, thus the increased fluorescence suggests movement of the dye into the hydrophobic core of the phospholipid bilayer due to compound 2 induced outer membrane permeation.^160^ Upregulation of stress response pathways was also demonstrated as a result of compound 2 treatment. Together these results demonstrate the self-associated compound 2 nanotubes as a bactericidal agent with activity derived *via* membrane interaction and permeation. MIC values generated against Gram-negative bacteria *Rhizobium radiobacter* (*R. radiobacter*) (ATCC 33970) (MIC 250 μg mL^−1^) and two Gram-positive bacterium, *Staphylococcus epidermidis* (*S. epidermidis*) (ATCC 12228) (MIC 250 μg mL^−1^) and *Listeria monocytogenes* (*L. monocytogenes*) (BUG 1361) (MIC 125 μg mL^−1^), also confirmed the broad-spectrum antimicrobial activity of the dipeptide.

**Fig. 6:**
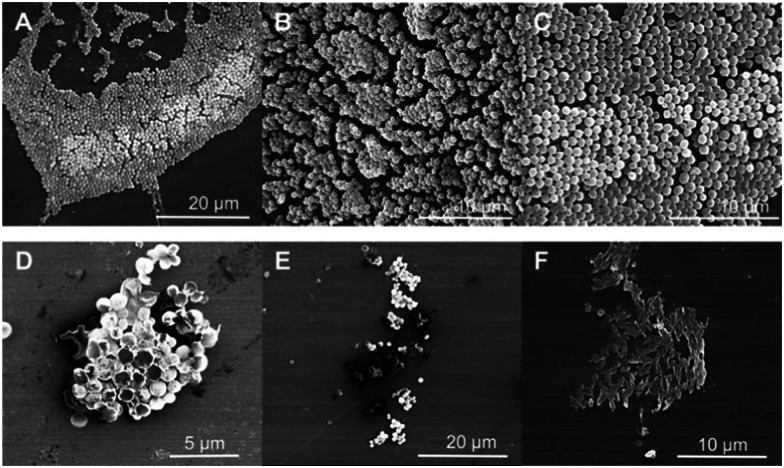
Scanning electron microscopy images of *S. aureus*. (a–c) Untreated *S. aureus* (NCTC 10788) biofilm after 24 hours. (d–f) *S. aureus* (NCTC 10788) biofilm after 24 hour treatment with increasing concentrations of 2.^158^ Reproduced with permission from Elsevier.

Furthermore, Porter *et al.* investigated the antibiofilm properties of the dipeptide 1.^158^ Three variants of 1 were tested against *S. aureus* (NCTC 10788) in both the planktonic (free living) and biofilm forms, with all three AMPs forming higher order nanotube structures. The tested AMPs consisted of 2 (NH_2_-FF-COOH), 3 (the d-enantiomeric isomer of NH_2_-FF-COOH), and 4 (NH_2_-FF-NH_2_), all shown in Fig. 5. While two of the modified FF peptides, 3 and 4, showed low levels of bactericidal activity, 2 displayed total biofilm disruption at 10 mg mL^−1^ against *S. aureus* over a 24 hour exposure. The measured antibiofilm activity was shown by SEM to be achieved through ion channel pore formation and surfactant-like activity, as shown in Fig. 6. Specific membrane association to bacterial cells was observed, despite the neutral charge of the compound 2 nanotubes. The authors highlighted that predicting favourable AMP membrane association qualities is still not well understood, and as stated previously, properties of hydrophobicity and amphipathicity are also important to AMP action. Thus, the neutral charge of the AMP may be necessary to maintain the properties of amphipathicity and hydrophobicity within effective ranges.

Specificity for bacterial membranes was demonstrated through cell viability assays, in which the peptides were shown to cause minimal toxicity to subcutaneous fibroblast cells, and no significant haemolysis compared to a negative control (phosphate buffered saline – PBS) in haemolysis assays. Together the results demonstrated this simple dipeptide system is efficacious against *S. aureus* in both its planktonic and biofilm forms. When considering Gram-negative bacteria, compound 2 showed a lack of antibacterial activity against *E. coli* (ATCC 15597), however it was able to penetrate the outer membrane of this bacteria, as shown using a fluorescent probe, that increases in fluorescent intensity when in hydrophobic environments. Whilst in this instance membrane penetration did not result in bacterial biofilm death, the observed membrane penetration may present a potential avenue for synergistic drug delivery against Gram-negative bacteria, serving like a trojan horse.

Utilising these smaller peptides as antimicrobials has many advantages. Chemical synthesis of short peptide sequences such as 1–4 offers cost effective manufacturing and purification, with high mechanical stability, good tissue penetration and decreased immunogenicity, overcoming many of the issues traditionally associated with AMPs.^161^

### 2.6. AMPs classified by building blocks

To achieve a specific self-assembled nanostructure, it is important to carefully consider the individual peptidic building blocks. These can be split into several categories. Building blocks that have been shown to act as self-assembling AMPs are listed below with some examples of their success. Due to the many benefits of shorter peptide sequences, these are the focus of this review section.

#### 2.6.1. Cyclic peptides

**Fig. 7:**
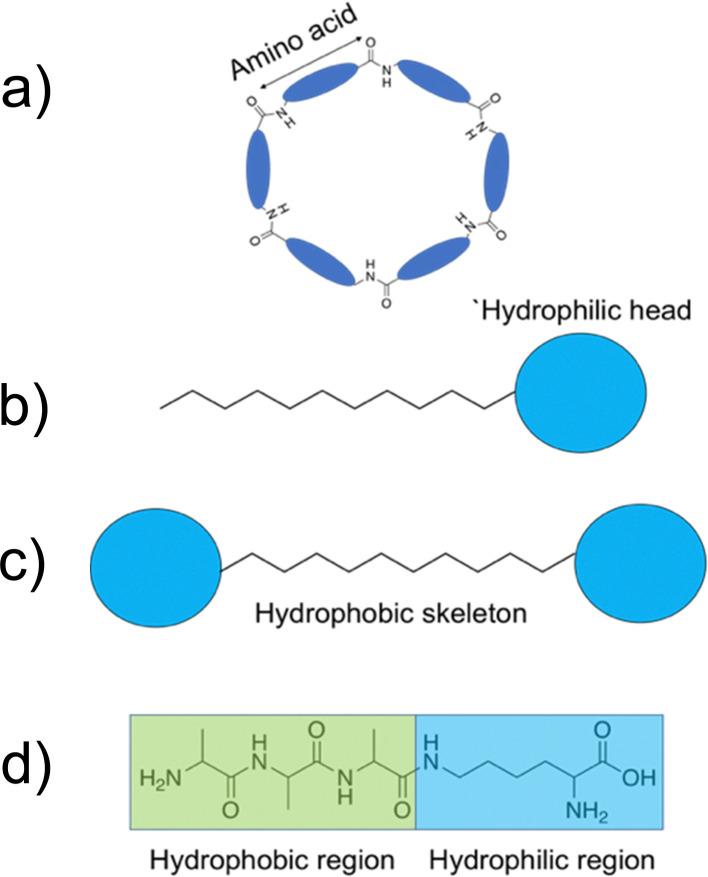
Example diagrams of common peptidic building blocks. (a) Cyclic peptides formed through amide bond formation of the terminal amino and carboxyl groups of the peptide. (b) Peptide amphiphiles. (c) Bolaamphiphiles displaying the hydrophilic head groups and hydrophobic tail/skeleton. (d) Amphiphilic surfactant-like peptides.

**Fig. 8:**
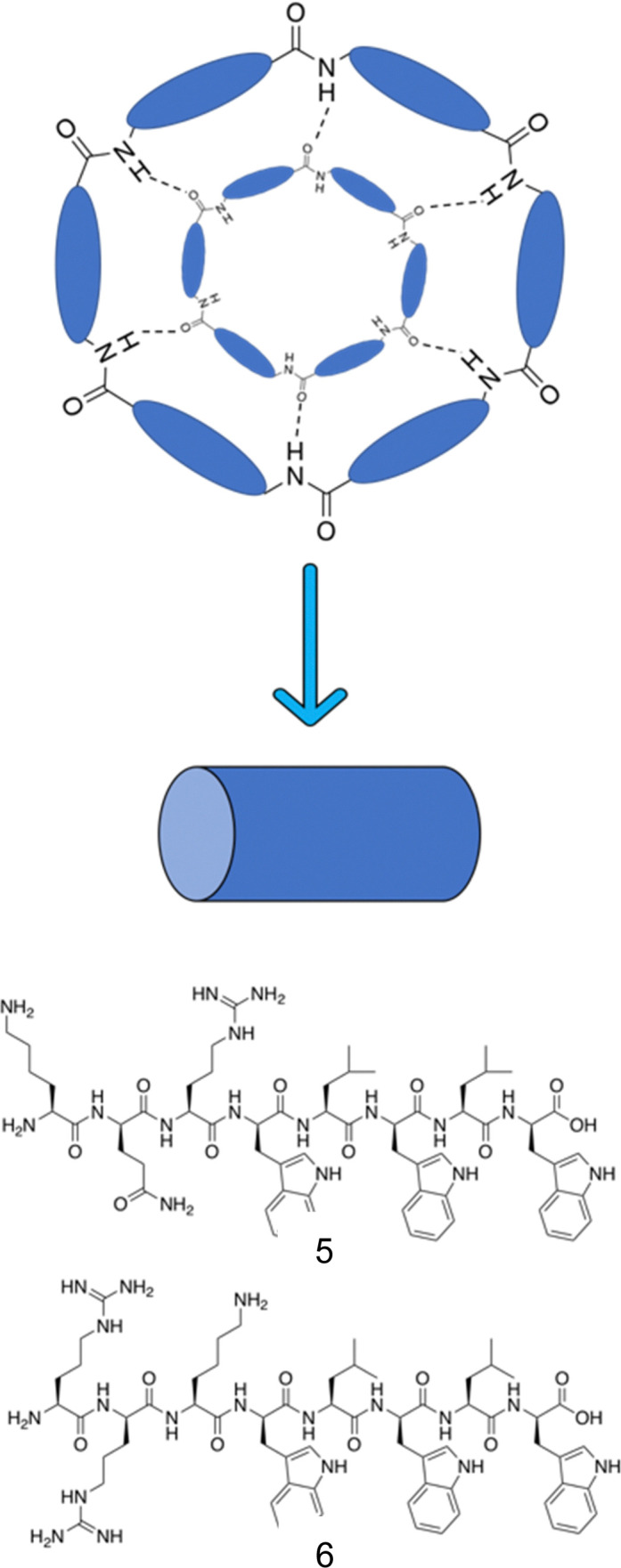
Representation of the antiparallel β-sheet formation from cyclic peptides. Alternating d- and l-amino acids displaying a cylindrical structure form antiparallel β-sheets through extensive intermolecular hydrogen bonding.^163^ Compounds 5 and 6 synthesised by Fernandez-Lopez *et al.*^166^

Cyclic peptides are polypeptide chains which form a ring structure, as exemplified in Fig. 7a. The cyclic structures are commonly produced through amide bond formation between the terminal amino and carboxyl groups of the peptide, or through formation of thioether and disulphide bonds.^162^ Cyclic peptides are capable of stacking to form cylindrical structures such as nanotubes, linked *via* intermolecular hydrogen bonds between stacked amide groups. Work by Ghadiri and colleagues demonstrated that cyclic peptides consisting of alternating d- and l-amino acids display a self-associated cylindrical structure with antiparallel β-sheets, formed through extensive intermolecular hydrogen bonding, presented in Fig. 8.^163^ Structurally, the amino acid side chains are orientated towards the outside of the cylinder, with the peptide backbone residing on the inner side.^164^ Due to the amino acid side groups residing on the outside of the structure, the functionalities are free to undergo intermolecular interactions with the external environment. As a result, it is possible to bestow a range of different physiochemical properties on the nanotubes through modifications to these side groups. Following association to lipid membranes, nanotubes formed from amphiphilic cyclic peptides commonly display a parallel orientation to the membrane plane.^165^ Acting through the carpet model, cell death is achieved through altered membrane potential, destabilising the membrane.^166^

Amphiphilic cyclic d,l-α-peptides developed by Fernandez-Lopez *et al.* demonstrated potent antimicrobial protection both *in vitro* and *in vivo* against methicillin resistant *S. aureus* (MRSA), offering promising clinical application.^166^ These peptides contain three consecutive hydrophilic residues followed by repeating alternate d-tryptophan and l-leucine amino acids. Compound 5 (Fig. 8) consisted of the sequence of KQRWLWLW (single letter amino acid code). The peptides were shown to self-assemble at the surface of synthetic membranes, forming nanotubular structures. Using multiple methods, namely: conductance measurements, vesicular ion and molecular transport pathways and Fourier transform-infrared spectroscopy (FTIR), the resulting tubular structures were shown to integrate into the membrane wall. Following integration, transport of ions and small species across the lipid bilayer was observed. A catalogue of peptides with minor alterations were developed from this sequence, ensuring at least one basic residue was present in each sequence, which when protonated under physiological conditions would enhance target specificity towards negatively charged bacterial membranes. Compound 6 (RRKWLWLW, Fig. 8) demonstrated the most promising balance between antimicrobial efficacy and cytotoxicity, with an MIC of 6 μg mL^−1^ against MRSA (ATCC 33591), 15 μg mL^−1^ against *E. coli* (JM109 (DE3)), and a haemolysis HD_50_ (concentration required to induce 50% of erythrocyte haemolysis^167^) of 50 μg mL^−1^. Quantitative measurements in oriented dimyristoyl phosphatidylcholine (DMPC) lipid multi-bilayers revealed formation of nanotubes, with their orientation consistent with the carpet-like mode of action. Investigations into proteolytic susceptibility of compound 6 revealed high levels of stability in the presence of the proteases trypsin, α-chymotrypsin, subtilisin as well as murine blood plasma. Finally, a pilot study conducted with mice to determine the efficacy of compound 6 for *in vivo* protection against MRSA and *E. coli* was conducted. Mice were infected with a lethal dose of MRSA, with either compound 6 or vehicle alone (the solution the peptide was given in) being administered intraperitoneally (into the body cavity) or subcutaneously (under the skin) 45 minutes post infection. All mice who received the control of vehicle alone died within 48 hours, while 67% of those receiving compound 6 intraperitoneally and 50% receiving the dose subcutaneously survived the course of the seven day study.^166^

#### 2.6.2. Peptide amphiphiles and bolaamphiphiles

Peptide amphiphiles consist of a polar hydrophilic region and an apolar hydrophobic aliphatic chain within their primary sequence.^95,96^ Most commonly, this is comprised of a peptide with a hydrophobic alkyl tail modification, shown in Fig. 7b. In aqueous solutions the hydrophobic tail causes the peptide building block to form three dimensional structures such as nanofibers, micelles and nanotapes.^168–170^ Bolaamphiphiles are comprised of two hydrophilic regions, linked by a hydrophobic skeleton, as shown in Fig. 7c.^171^ Similarly to peptide amphiphiles, peptide bolaamphiphiles are capable of forming a diverse array of nanostructures, some of which are shown in Fig. 2.

**Fig. 9:**
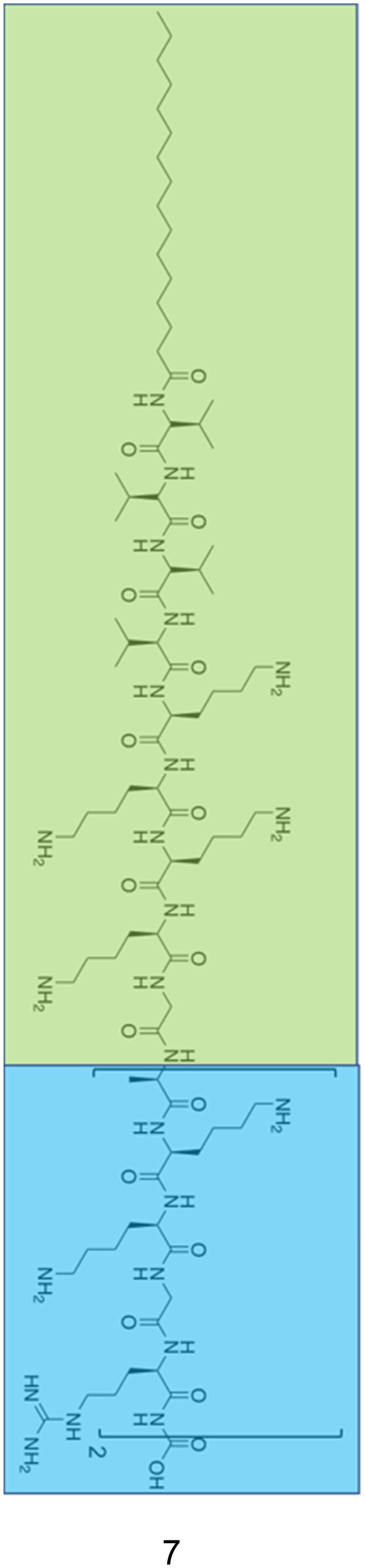
The primary structure of compound 7 synthesised by Chang *et al.*^138^ The amphiphilic portion of 7 is shown in the green box, while the heparin-binding Cardin-motif is shown in the blue box.

By functionalizing peptides with a heparin-binding cardin-motif (*e.g.* compound 7, Fig. 9), Chang *et al.*^138^ produced peptides amphiphilic in nature, with potent antibacterial activity, showing self-assembly of the peptide to be critical for antibacterial activity towards Gram-negative bacteria. The critical micelle concentration (CMC) for 7 was determined using Nile Red dye, which is solubilised in the hydrophobic core of compound 7 structures upon their formation, consequently enhancing fluorescent intensity. By observing the fluorescence intensity maxima, the CMC was determined at 50.1 μM. Concentration dependent formation of nanostructures was observed using transmission electron microscopy (TEM), with bundled and elongated nanofibers observed at 2 mg mL^−1^, and nanorods with diameters of 7–10 nm at 1 mg mL^−1^. Investigation into the antimicrobial activity for compound 7 was conducted against the Gram-positive *S. aureus* (ATCC 25923) and MRSA (ATCC 43300), and the Gram-negative *E. coli* (ATCC 25922) and MDR *E. coli* (ATCC BAA-2471). For the Gram-positive bacteria, initial experiments observed delays to bacterial exponential growth at all tested concentrations of compound 7 (20–100 μM), including those below the CMC (50.1 μM). The highest tested concentration of compound 7 (100 μM) caused a delayed time to exponential growth phase compared to the control by 14 and 9 hours for *S. aureus* (ATCC 25923) and MRSA (ATCC 43300) respectively. Viable colony counting assays revealed a step-wise concentration dependent activity against both the Gram-positive bacteria from 20–100 μM, with 80 μM inducing a 2 log reduction in colony forming units (CFU). Subsequent live/dead assays indicated significant reductions in MRSA viability after four-hour treatment with compound 7 at both 40 μM and 80 μM, while TEM imaging of MRSA treated with 40 μM of compound 7 displayed a damaged outer membrane, with a detached cytoplasmic membrane resulting in cytoplasmic leakage. In comparison, the Gram-negative bacteria displayed no significant delay in bacterial growth when treated with compound 7 below its CMC (50.1 μM), while at concentrations above 80 μM MDR *E. coli* (ATCC BAA-2471) showed complete inhibition of growth, while *E. coli* (ATCC 25922) exhibited significantly delayed growth. Viable colony counting assays further evidenced the sensitivity of Gram-negative bacteria to the higher order structures produced through the self-assembly of 7, whereby concentrations below the CMC showed no significant bactericidal effects, while at a concentration of 60 μM compound 7 displayed a 5 log reduction in CFU, with a similar result observed for 80 μM and 100 μM of compound 7 against both Gram-negative bacteria. Live/dead assays performed on Gram-negative MDR *E. coli* displayed no considerable cell mortality after treatment with 40 μM of compound 7, while drastic increases in cell mortality were observed when treated with the increased concentration of 80 μM. TEM corroborated the aforementioned results, with 40 μM MDR *E. coli* treated cells only displaying a blistered outer membrane, with an intact cytoplasmic membrane and no leakage. When increased to 80 μM, disrupted bacterial envelopes with a disconnected membranous structure were observed. Together, these results again showcase the importance of molecular self-assembly on the antibacterial activity of compound 7 against the Gram-negative bacteria tested, while a concentration dependent response was determined for the Gram-positive bacteria. To elucidate the binding capabilities of compound 7 to LPS (Fig. 3a) of *E. coli*, a BODIPY TR cadaverine (BC) fluorescent probe displacement assay was conducted. Here, the stronger binding of compound 7 to LPS competes with BC binding, causing displacement of the BC dye from the LPS, and as a consequence, increased fluorescence emission. Results confirmed strong interactions of compound 7 with LPS at concentrations from 20 μM to 100 μM, with no statistically significant variation in the binding strength across this concentration range. Finally, cytotoxicity assays performed against human dermal fibroblasts (HDFs) revealed 7 displayed significantly lower toxicity against HDFs than observed against the bacteria tested. Specifically, up to concentrations of 60 μM minimal cytotoxicity was induced, while at the highest concentration of 100 μM, 50% HDF viability was observed.

**Fig. 10:**
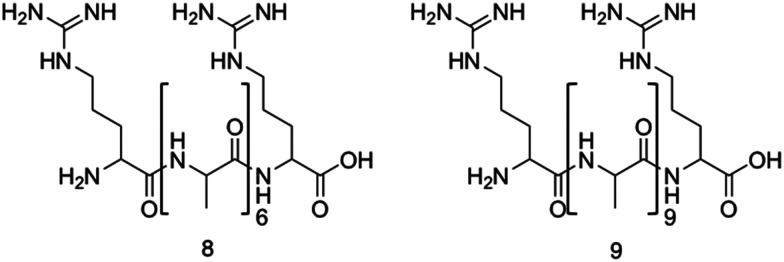
The primary structure of compounds 8 and 9. The alanine residue is displayed in brackets, while the two arginine residues are located on either side of the alanine.^172^

Two arginine capped bolaamphiphile peptides were synthesised by Edwards-Gayle *et al.* and shown to elicit broad spectrum antimicrobial activity.^172^ These peptides were developed around the generic structure of arginine–(alanine)_*x*_–arginine (Fig. 10). Following successful synthesis, self-assembling propensities were investigated. Compound 8 (Fig. 10), with 6 alanine residues, displayed no self-assembly in water, while compound 9, with 9 alanine residues (Fig. 10) formed amyloid fibres, with increasing concentrations resulting in formation of β-sheet structures. Formation of nanostructures from compound 9 were hypothesised to be a result of the hydrophobic nature of the A_9_ block. Interactions of these peptides with model dipalmitoyl-phosphatidylglycerol/dipalmitoyl-phosphatidylethanolamine (DPPG/DPPE) vesicles were investigated using differential scanning calorimetry (DSC). These vesicles were selected as these lipids constitute key components of bacterial membranes, therefore can be used to model bacterial cell surfaces.^173^ DSC results indicated lipid demixing for both 8 and 9, consistent with that expected from electrostatic interactions between the peptides and lipid membranes. Probing for structural changes resulting upon exposure to the DPPG/DPPE lipids was conducted utilising circular dichroism (CD), with results indicating no change in structure for compound 8 at the vesicle surface, while compound 9 presented a transition to a β-sheet conformation. The lack of secondary structure formation from compound 8 was proposed to be due to strong binding of the DPPG lipid head group with the AMP, while the increased peptide concentration of compound 9 occurring at the membrane was proposed to be responsible for the observed β-sheet formation of compound 9. Following membrane interaction studies, investigations into antimicrobial potency revealed potential for compound 8 as an antibacterial agent against *Pseudomonas* bacteria. Specifically, a 4 log reduction in CFU was observed against *Pseudomonas aeruginosa*^174^ (*P. aeruginosa*) at 0.1 wt% of compound 8 after 24 hours during bacterial kill assays, a concentration well tolerated by fibroblasts (∼72% viability). Additionally, a similarly high reduction of 4.5 log was observed against *Pseudomonas syringae* (*P. syringae*), also at 0.1 wt%. Compound 9 presented a small statistically insignificant reduction of *E. coli*^175^ numbers at a concentration of 0.1 wt%, while reductions in CFU of 2.6, 3.4 and 4.0 orders of magnitude were observed for *S. aureus*, *P. aeruginosa* and *P. syringae*. Together, these data showed compound 9 displayed a broad range of activity against both Gram-positive and Gram-negative bacteria, while compound 8 presented strong activity against *Pseudomonas* bacteria at concentrations displaying acceptable cytocompatibility, highlighting the potential for bolaamphiphiles as antimicrobial agents.

#### 2.6.3. Surfactant-like peptides

Surfactant-like peptides (SLP) refer to peptides that demonstrate a reductive effect on the surface tension of water.^96^ Unlike peptide amphiphiles, SLPs exclusively consist of amino acids, with no aliphatic chain modifications,^95^ as shown in Fig. 7d. SLPs are normally made of fewer than 10 amino acids and demonstrate solubility in both organic solvents and water.^95^ The short sequences commonly seen with SLPs offer advantages of cheaper and faster synthesis, key properties required for large scale pharmaceutical application. SLPs have been shown to form both nanovesicles and nanotubes.^176^

The synthesis of short aliphatic surfactant-like peptides; compound 10 (A_3_K), 11 (A_6_K), 12 (A_9_K), and the resulting properties incurred through the increasing hydrophobic alanine sequence was investigated by Chen *et al.*^177^ The structure of 10 is presented in Fig. 7d, with 11 and 12 differing by the number of alanine residues. Formation of self-assembled structures was observed for all three peptides using atomic force microscopy (AFM), with compound 10 forming loose unstable stacks, compound 11 forming long and stable nanofibers and compound 12 forming short, narrow well packed rods. These observations indicated an increased propensity for ordered self-assembly with increased hydrophobicity. In parallel with these findings, hydrophobicity was also correlated with antimicrobial efficacy against both Gram-positive and Gram-negative bacteria. Specifically, compound 10 caused no significant bacterial death up to its highest tested concentration, compound 11 caused reductions of 35% in *E. coli* (DH5α) and 45% in *S. aureus* (ATCC 25923) at 0.2 mg mL^−1^, while compound 12 caused 80% of *E. coli* and 70% of *S. aureus* to be killed after one-hour treatment at 0.1 mg mL^−1^. Importantly, compound 12 was shown to display no measurable human RBC haemolysis at this same concentration (0.1 mg mL^−1^), indicating good cytocompatibility at effective antimicrobial concentrations. Additional bactericidal investigations of compound 12 revealed a reduction of 55% of *E. coli* occurred in the first 10 minutes of application, while it took 30 minutes for the same reduction in *S. aureus*. Following the confirmed antimicrobial activity, the mechanism of action of compound 12 against the bacteria cell membranes was shown to be *via* insertion of nanorods, causing the formation of a barrel-stave or micelles (shown in Fig. 4a), leading to cell leakage and lysis confirmed by SEM and fluorescence microscopy.

### 2.7. Unique properties of antimicrobial peptides

The assembly of nanostructures can be controlled through manipulation of pH, electrolytes, biological factors and temperature.^178^ In self-assembling systems where the formation of nanostructures is vital to the mechanism of action, control of this process enables a form of ‘on/off’ switch.^179^ Chen *et al.* utilised changes in pH to selectively change the surface charge of amino acids in peptide amphiphiles, either promoting or discouraging the formation of nanofibers.^180^ Future studies may present a potential use for such switchable self-assembling AMPs to improve efficacy against bacterial cells and reduce potential toxicity.

While self-assembling AMPs have inherent antimicrobial activity, the formation of nanostructures also allows potential for drug release to be instilled within the design. Such delivery systems could allow for increased efficacy of a drug at the target site due to the localisation and bacterial selectivity of these structures, improving both the pharmacokinetic (PK) and pharmacodynamic (PD) properties.^181^ Self-assembling peptides have already been utilised for the delivery of chemotherapeutic agents in cancer, as well as gene delivery vehicles, therefore this technological approach may lend itself to future antimicrobial applications.^182,183^ Nanofibers,^184^ hydrogels^185^ and nanotubes^186^ all present opportunities for targeted drug release systems. Further benefits of stimuli responsive drug release include controlling the kinetics of drug release and manipulating the ratio of released therapeutics.^187^

## 3. Self-assembling small molecules and macromolecules as antimicrobial agents

Alongside the continued drive for the enhancement of self-assembling AMP efficacy and cytocompatibility, attention has been cast on developing fully synthetic alternatives to self-assembling AMPs.^188^ Self-assembling AMPs synthetic counterparts offer the advantage of bottom-up synthesis. With considered design, alleviation of issues often associated with self-assembling AMPs is possible;^189,190^ we direct the reader to other more focussed reviews on these topics.^191–194^ For the purposes of this review, these synthetic alternatives have been subdivided into two categories based on the building blocks used, namely; self-assembling small molecules and self-assembling macromolecules.

Macromolecules are defined as any compound over 1000 Daltons.^195^ Specifically, sizes of macromolecules that have been shown to elicit antimicrobial activity range from >10 kDa to 100 kDa.^196^ Conversely, small molecules are defined as compounds under 1000 Da.^190,197^ Although small molecules are abundant in the pharmaceutical industry, accounting for 90% of drugs on the market and totalling 166 billion in the chemical universe database, many of these small molecules focus on targeting receptor mediated mechanisms.^190,198,199^ This section of the review focuses on self-assembling small-molecules and self-assembling macromolecules that, through nanostructure assembly, have successfully achieved mechanisms of action independent of receptor mediated mechanisms, similar to self-assembling AMPs.^191,196^

### 3.1. Self-assembling small molecule antimicrobials

#### 3.1.1. Advantages and disadvantages of small molecule antimicrobials

It is important to understand that most of the benefits of small molecules lie in their general simplicity and well-established performance *in vivo*. Firstly, small molecules can offer more controlled and predictable synthesis compared to their macromolecular counterparts, accompanied with increased ease of scale up production, chemical characterisation and decreased manufacturing costs.^200^ Due to the high AMR prevalence in third world countries, considerations such as cost cannot be underestimated.^201^ Small molecules are also amenable to high-throughput screening, which enables the identification of large libraries of potential drug candidates in vastly reduced experimental time frames, further reducing development costs. Successes of high-throughput screening have led to investigations into other forms of compound, including peptides.^202–205^ Secondly, small molecules have been extensively studied with respect to their PK and PD properties, and as such, a host of tools and guidelines for achieving favourable performances *in vivo* are available.^206^ For instance, the absorption, distribution, metabolism and excretion (ADME) processes of small molecules are much better characterized than that of macromolecules,^207^ with rules such as Lipinski's rule of five criteria, offering a set of ‘druggability’ guidelines.^208^ These rules state that drugs with more than 5 hydrogen bond donors, 10 hydrogen bond acceptors, MW above 500 and log*P* value above 5 are likely to have poor absorption and permeation across biological membranes.^209,210^ For example, 39% of clinical trials were halted due to poor absorption of the therapeutic candidate in 1991. However, through the implementation of Lipinski's rule of five in 1997, the number of clinical candidates exiting clinical trials for this same reason had dropped to 8% by the year 2000.^211^ Unfortunately, macromolecules and AMPs do not fit within these guidelines.^212^ Thirdly, extensive study has been conducted into developing computational systems predicting drug-like properties. Drug-likeness, an important parameter referring to the delicate balance of molecular properties of a compound, is often used for selecting compounds for screening and computational modelling for drug development.^213^ Thus improved prediction of drug-like compounds results in more efficient lead optimisation. Cutting-edge tools including machine learning have further enhanced drug prediction models, improving lead candidate optimisation reducing the cost of drug development.^214,215^

Despite the simplicity of small molecules leading to these benefits, many of the advantages that macromolecules and AMPs offer are lost. For instance, the simplicity of small molecule drugs often render them unresponsive to negative feedback mechanisms, displaying no response to changes in the physiological environment.^216^ When optimising therapeutic agent binding in deep pockets or grooves, such as active sites of enzymes or ligand binding sites, the size of small molecules enables high affinity binding.^217^ However, when targeting biological membranes where multivalent binding is advantageous, the low number of chemical moieties within a small molecule can reduce optimal binding affinity, reducing selectivity in comparison to AMPs and macromolecules.^216,218^ As a result of reduced specificity, off site biological effects are often observed for this class of antimicrobial agents.^219^

#### 3.1.2. Current small molecule antimicrobials under investigation

**Fig. 11:**
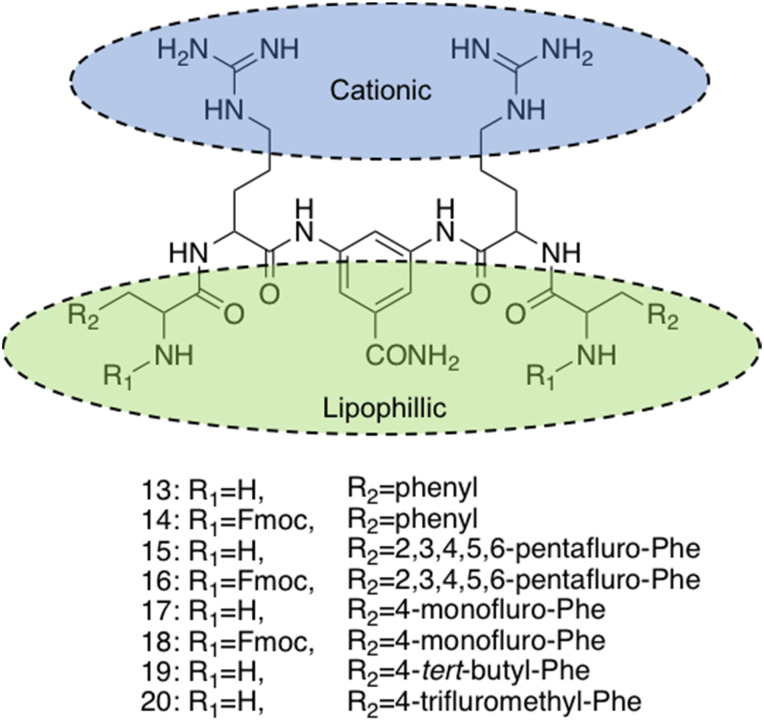
Compounds 13–20 developed by Thota *et al.*^220^

Various synthetic designs have been employed to develop small molecules mimicking the self-assembling AMP like membrane targeting mechanism of action. An investigation conducted by Thota *et al.* developed compounds 13–20, consisting of a 3,5-diaminobenzoic acid scaffold with ultrashort amino acid sequences attached onto the exocyclic amino arms (Fig. 11), achieving antimicrobial efficacy against *S. aureus* and *Micrococcus luteus* (*M. luteus*) comparable to that of vancomycin and the AMP Gramicidin.^220^ The aforementioned amino acids introduced into the exocyclic amino arms were arginine and l-phenylalanine (or its fluorinated or alkylated derivative), which when protonated under biological conditions, result in a positively charged compound. This enables effective bacterial membrane targeting (through electrostatic interactions as previously discussed in the self-assembling peptide section) and lipophilic/self-assembly properties respectively. Through the adaptation of this scaffold, a library of eight compounds were synthesised, shown in Fig. 11.

The self-assembly properties of this class of compounds (13–20) was established through the use of TEM, cryo-TEM and thioflavin T fluorescence assays (a fluorescence assay used to detect fibril formation),^221^ with the results indicating that only compounds 16 and 19 self-assembled in phosphate buffer at pH 7.4.^222^ Specifically, cryo-TEM showed compound 16 to form nanofibers with diameters of 10–28 nm, while 19 formed patched micellar nanoparticles with ≈10 nm diameter, existing in monomeric, oligomeric (through stacking) and multimeric states. The authors attributed the self-assembly of these two compounds to the bulkiness, hydrophobicity and π–π stacking of the aromatic residues in the R groups, based on the observed structure-activity relationships attained from the library of compounds. Next, the antimicrobial activity of the compounds 13–20 was investigated against both spherical (cocci) and rod-shaped (bacilli) Gram-positive bacteria, in addition to rod shaped Gram-negative bacteria. The Gram-negative bacteria included within the scope of these studies are as follows: *E. coli* (DSM 116) (bacilli), *Salmonella typhimurium* (*S. typhimurium*) (TA100) (bacili) and *P. aeruginosa* (ATCC 15442) (bacilli). The Gram-positive bacteria included within the scope of these studies included: *Bacillus subtilis* (*B. subtilis*) (DSM 10) (bacilli) and acid-fast strain *Mycobacterium phlei* (*M. phlei*) (DSM) (bacilli), *M. luteus* (DSM 1790) (cocci) and *S. aureus* (ATCC 25923) (cocci). Compounds 14, 16, 18 and 19 were all shown to display specific activity against the cocci shaped *M. luteus* and *S. aureus*. It was highlighted that 14, 16 and 18 all included an N-terminal Fmoc group, while 13, 15 and 17 all lacked this Fmoc moiety, indicating the bulky hydrophobic groups were important in the resultant antimicrobial activity observed. The two self-assembled compounds, 16 and 19 displayed the most potent antimicrobial activity, with compound 19 displaying the lowest MIC of 1.9 μg mL^−1^ and 3.9 μg mL^−1^ against *M. luteus* and *S. aureus* respectively. Compound 19 was calculated to have a theoretical net charge of +4 compared the theoretical net charge of +2 for 16, with this difference reasoned to be responsible for the increased activity of 19 over that of 16, because of enhanced electrostatic interactions with anionic bacterial membranes. Interestingly, the MIC's of 19 were almost equal to those achieved by the potent antimicrobial vancomycin (2 μg mL^−1^ against both strains) and Gramicidin. Importantly, the following cytotoxicity studies revealed no significant haemolytic activity was observed against human RBCs for compound 19 up to 100 μg mL^−1^, a concentration 50-fold higher than its MIC in *M. luteus* and 25-fold higher than its MIC in *S. aureus*. Haemolytic activity is a common issue observed with AMPs and is therefore a key focus point for synthetic alternatives to mitigate. SEM was conducted on compound 19 treated cells to elucidate the mechanism by which antimicrobial activity was achieved against *M. luteus*. The resulting images revealed a coarse appearance on the surface of compound 19 treated *M. luteus* cells, indicating bacterial cell wall rupture was responsible for the bacterial cell death observed.

**Fig. 12:**
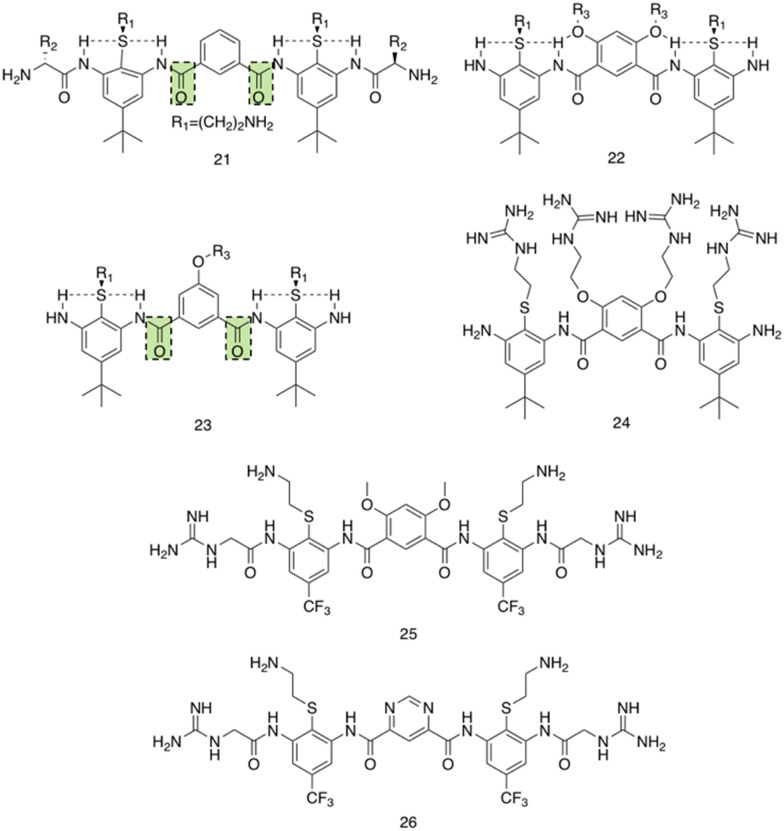
Compounds 21–26.^223^ Green boxes highlight the N–C torsional bond between the phenyl ring and amide carbon. The dotted line indicates hydrogen bonding. R groups in compounds 21–23 were varied in hydrophobicity and charge.

Utilising a different form of AMP mimetic, Choi *et al.* developed small molecule acrylamide foldamers to overcome the fall backs commonly associated with AMPs, namely; large size, stability, tissue distribution and cytotoxicity.^223^ The resulting compound, compound 26 (Fig. 12), demonstrated potent *in vivo* activity similar to that of vancomycin at its maximum tolerated dose. Previously the authors developed the acrylamide foldamer, 21 (Fig. 12), containing two 1,3-phenylene diamine units connected by an isophthalic acid.^224^ A thioether moiety provided a point of attachment to the basic groups, in addition to forming intramolecular hydrogen bonds to neighbouring amides. This structure restricted rotation about the N–C torsional bond between the phenyl ring and amide carbon (see green boxes in Fig. 12). This design was then improved upon; the second iteration, compound 22 (Fig. 12), incorporating a 4,6-dialkoxy-substituted isophthalic acid linker, increasing the rigidity of the acrylamide scaffold through formation of intramolecular OH–N hydrogen bonds. A further derivative 23, with a single alkoxyl group introduced at the 5-position of the isophthalic acid ring system, was also developed to measure the effect of conformational restraint in compound 22 on antimicrobial activity. This series of step-wise modified compounds enable the elucidation of structure activity relationships for this class of acrylamide foldamer to be determined. Specifically, the effect of the pendant functional groups marked ‘R_1_’ and ‘R_3_’ which provide variations in molecular charge and hydrophobicity.

In addition, the antimicrobial activities of compounds 21–26 have also been established against the Gram-positive *S. aureus* (ATCC 27660) and Gram-negative *E. coli* (D31). Observing the trends in the resulting MICs of the compounds revealed a concomitant increase in affinity and selectivity towards both bacteria with increased rigidity of the molecular structure. Compounds with less rigid structures required a greater number of charged groups to produce similar antibacterial activity compared to their more rigid counterparts. It was also observed that only the groups with the highest number of potential hydrogen bonded conformational restraints displayed good *in vivo* activity in a mouse thigh burden infection model. This model involved the inoculation of mouse thigh muscle with bacteria, followed by intravenous administration of the compound under investigation, with compounds 25 and 26 displaying activities similar to that of vancomycin. Specifically, both 25 and 26 could be tolerated up to a dose of 20 mg kg^−1^, with 26 showing a x10^5^ reduction in viable CFU of *S. aureus* at 2 mg kg^−1^, a result comparable to vancomycin at its maximum efficacious dose of 30 mg kg^−1^ in the same model. Furthermore, resistance studies were performed, whereby one passage consisted of *S. aureus* being exposed to compound 24, or a control (ciprofloxacin or norfloxacin) at a sublethal concentration, followed by MIC elucidation. The results from this study revealed no decrease in MIC of compound 24 against *S. aureus* after 16 passages, with the fluoroquinolones ciprofloxacin and norfloxacin presenting increased MICs after 6 passages. Thus, it is evident resistance could not easily be formed against this acrylamide foldamer. Additionally, cytotoxicity trials indicated high selectivity for both *S. aureus* and *E. coli* over mammalian cells, with HC_50_ values for compounds 25 and 26 presenting values 100-fold higher than their MICs against *S. aureus*. Together this demonstrates these acrylamide foldamers present potent activity against *S. aureus*, low susceptibility to resistance and good safety profiles, which combined indicate promising clinical developmental prospects.

##### 3.1.2.1. Antimicrobial amphiphiles

When considering small molecule amphiphiles, the hydrophobic component traditionally consists of a hydrocarbon chain, while the hydrophilic element can be either ionic or neutral but with highly polar functional groups.^225^ Anionic surfactants contain positively charged counter ions (*i.e.* sodium, potassium), while cationic surfactants often contain halide counter ions.^225^ Zwitterionic amphiphiles contain both anionic and cationic headgroups covalently bonded to the hydrocarbon chain.^226^ The thermodynamic incompatibility created between the hydrophobic and hydrophilic blocks within the molecule promotes spatial organization into ordered morphologies, with self-assembly driven by supramolecular forces such as hydrogen bonding and electrostatic interaction.^227^

**Fig. 13:**
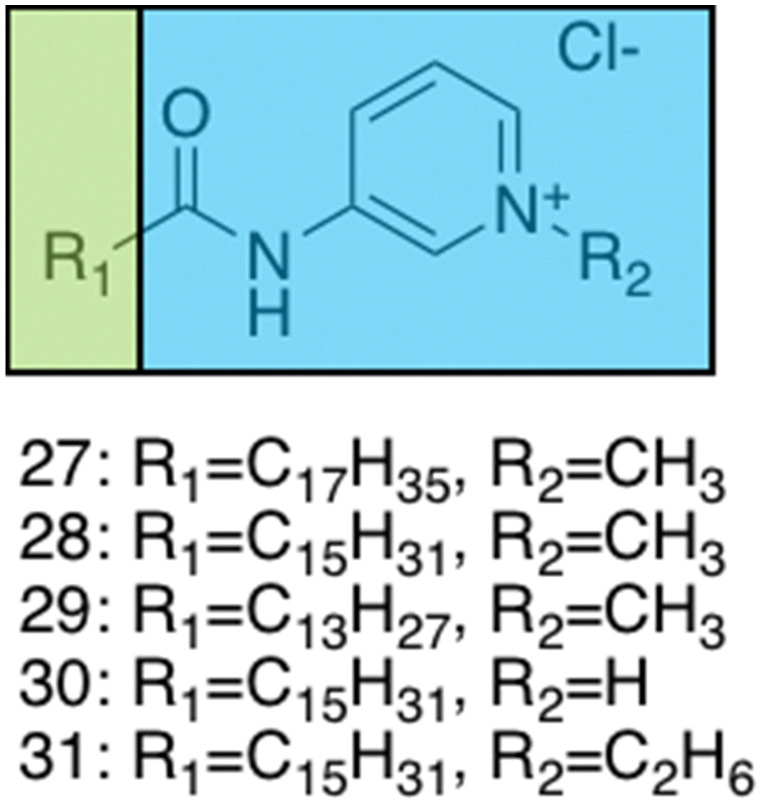
Generic structure and R groups for compounds 27–31 developed by Brahmachari *et al.*^228^ Green indicates hydrophobic tail. Blue indicates hydrophilic head.

Brahmachari *et al.* have developed several amphiphilic small molecule compounds capable of forming hydrogels, achieving potent MICs against both Gram-positive and Gram-negative bacteria.^228^ These compounds contain a polar head group consisting of a quaternary pyridinium unit, coupled to a hydrophobic alkyl chain *via* an amide bond (Fig. 13).^228^ A library of compounds, 27–31, were synthesised displaying varying hydrophobic alky chain lengths, with modifications also made to the R_2_ group on the polar head, allowing a structure property relationship to be derived with respect to molecular gelation processes. Through determination of the minimum gelation concentration (MGC) and resulting stability of the gel over time, the hydrophobic-hydrophilic balance was shown to be crucial for gel formation, with slight modifications in hydrophobic chain length preventing gelation. Specifically, only compounds 27 and 28 were shown to successfully form hydrogels, with SEM revealing an aggregated porous network for compound 27 and a thin intertwined fibrillar network for 28. By exciting increasing concentrations of compound 28 at 330 nm and measuring the resulting emission spectra between 340–550 nm, a red shift in the emission peak revealed that π–π interactions between the pyridine moieties played an important role in the resulting self-assembly.^229^ Further validation of this initial observation was obtained through the use of NMR, with 2D NOESY spectra for 28 displaying diagonal cross peaks between the signals relating to the pyridine systems and the methyl group attached to that same pyridine system, suggesting that the two groups were spatially orientated in close proximity. These spectroscopic results, in combination with X-ray diffraction and microscopic measurements of the resulting xerogel suggested formation of repeating bilayers with the molecules connected by intermolecular hydrogen bonding and complementary hydrophobic tail group interaction.

Following on from these structural studies, the antimicrobial activity of compounds 27 and 28 were investigated against both the Gram-negative *E. coli* and *Klebsiella aerogenes* (*K. aerogenes*) and Gram-positive *B. subtilis* and *M. luteus.* MICs obtained for compounds 27 and 28 were between 5.0–20.0 μg mL^−1^ against the two Gram-negative bacteria and 0.4–2.0 μg mL^−1^ against the two Gram-positive bacteria, showing potent broad-spectrum activity. The authors highlighted activity against Gram-negative bacteria as particularly interesting, with other conventional quaternary cationic amphiphiles normally presenting low activities against these species of bacteria.^228^ Both compounds were suggested to act *via* adsorption of the cationic amphiphile onto the negatively charged cell membrane through electrostatic interactions, with high entropic favourability due to the release of counter ions. Resulting penetration of the hydrophobic chain into the hydrophobic cell membrane leading to the release of cytoplasmic contents, was speculated to be the cause of cell death.^230^ Promisingly, cytotoxicity of compound 28 against fibroblast cells (NIH3T3) determined utilising an methylthiazolydiphenyltetrazolium bromide (MTT) assay (a colorimetric assay, used to determine the number of metabolically active cells *via* reduction of yellow tetrazolium salts to purple formazan crystals),^231^ indicated the viability of 96% of the fibroblast cells when treated with up to 20 μg mL^−1^ of 28, with over 50% viability up to 100 μg mL^−1^. Thus, at a therapeutic concentration this compound does not exhibit toxicity against these representative human cells.

**Fig. 14:**
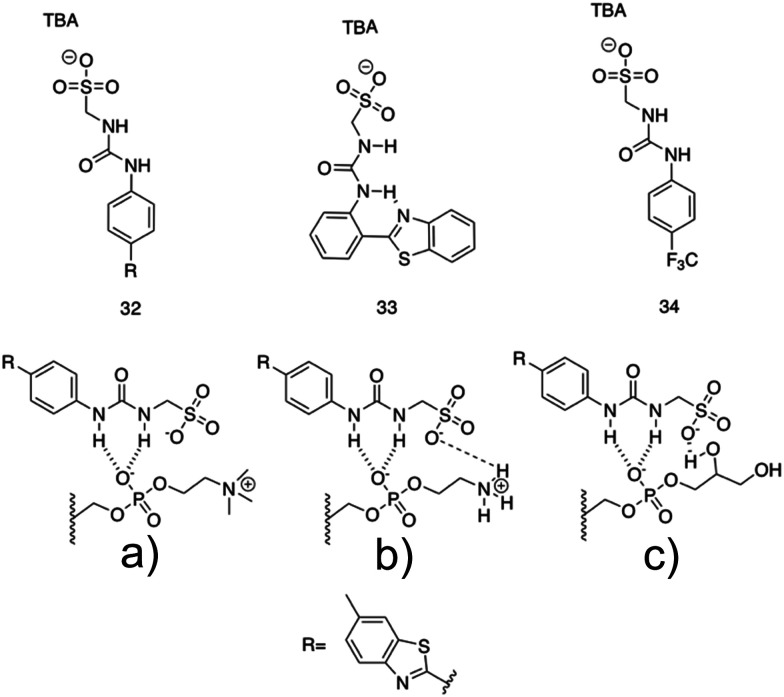
Compounds 32–34 with TBA counter cation. The possible binding modes of the SSA anionic component with (a) phosphatidylcholine (PC), (b) phosphatidylethanolamine (PE) and (c) phosphatidyl glycerol (PG) phospholipid headgroups.^232^

A novel class of supramolecular self-associating amphiphilic salts (SSAs) developed by Hiscock and co-workers have been shown to demonstrate antimicrobial activity against both MRSA and *E. coli*.^232^ Previously, a library of 50 novel SSA compounds was synthesised and screened for antimicrobial efficacy, allowing elucidation of structure-activity models from 14 physiochemical parameters simultaneously, whilst also supplying evidence for bacterial membrane binding.^233^ In a subsequent study the anionic component of the investigated SSA compounds consisted of a hydrogen bond donor-acceptor thio/urea array, and was found to adopt multiple hydrogen bonding modes simultaneously, due to the uneven number of hydrogen bond donating and accepting groups.^232^ These hydrogen bonding modes were determined to be dependent on the coordination strength of the counter cation present, with tetrabutylammonium (TBA) promoting thio/urea anion dimers, which were shown to prevail in both the gas phase and within DMSO solvent systems. When looking at this same class of SSA in aqueous solutions, spherical aggregates with a hydrodynamic diameter of 100–550 nm were shown to form. Three of these SSA compounds, 32–34, (Fig. 14) were selected for investigation into antimicrobial activity against MRSA (USA300) and *E. coli* (DH10β), due to the hypothesised preferential binding of SSAs to PE and PG (Fig. 3c) lipid head groups (Fig. 14a–c), both of which are present at the surface of bacterial cells,^113^ a hypothesis substantiated using phospholipid nanodisc assays.^234^

All three compounds, 32–34 showed antimicrobial activity, with MIC_50_ values of 0.46–0.93 mM against MRSA and 3.85–5.02 mM against *E. coli*. Furthermore, following heating, self-assembly of compound 32 into a hydrogel was observed in a range of salt solutions. The resulting fibres were imaged utilising SEM and fluorescence microscopy, made possible due to the inherently fluorescent nature of the benzothiazole unit. Investigation into the possible use for compound 32 hydrogels as a topical antimicrobial treatment were conducted, with the authors highlighting the need of such materials due to the commonly used antiseptics (triclosan and chlorhexidine) being banned by the FDA in fear of cross resistance^235,236^ and harm to human health.^237^ To elucidate the ideal salt solution for a topical treatment, several metrics were measured and compared. Results indicated NaCl as the optimum solution due to the pH falling within the physiological skin pH range (4–7).^238^ Here the material melting temperature (*T*_m_) was found to be >50 °C, enabling stability during administration and storage, the observed MGC was found to be low, NaCl presented no significant toxicity, and favourable viscoelastic properties were observed during rheological measurements. The observed elasticity under load avoids breakdown, while viscosity at rest allows large surface area coverage.^232^ Next, biological experiments were conducted to ensure antimicrobial activity was maintained by the SSA in its hydrogel form. Compound 32 hydrogels (50 mg) were applied to agar plates with a lawn of MRSA or *E. coli* and incubated overnight at 37 °C. The following day both plates presented zones of inhibition localised to the hydrogel site, confirming their antimicrobial activity. The resulting lack of diffusion from the hydrogel site was expected due to the incorporation of compound 32 into the hydrogel fibres, confirmed through fluorescence microscopy studies. Furthermore, investigation into the ability of this hydrogel to act as a drug delivery system for small molecule antibiotics was conducted. Ampicillin was incorporated at a 1 : 1 ratio with compound 32 into the SSA hydrogel and well diffusion assays were performed on the resulting material. Here, compound 32 hydrogels containing ampicillin were placed into a well in the centre of an agar plate inoculated with *E. coli* and left incubating at 37 °C overnight. The resulting zone of inhibition observed for the ampicillin containing hydrogel of compound 32 was found to be similar to that of the control plate containing only ampicillin, suggesting free diffusion of ampicillin from the hydrogel. Hiscock and co-workers also investigated their SSA compounds as enhancers of antimicrobial agents towards *E. coli* (DH10β) and *P. aeruginosa* (PAO1).^239,240^ In these studies, one such SSA displayed enhanced efficacy of cisplatin, ampicillin and octenidine, whilst several other SSAs enhanced the efficacy of novobiocin and rifampicin, confirming SSAs can also increase the activity of other drugs to achieve antimicrobial activity. Thus, the SSAs represent a new class of multifunctional antibacterial materials.

**Fig. 15:**
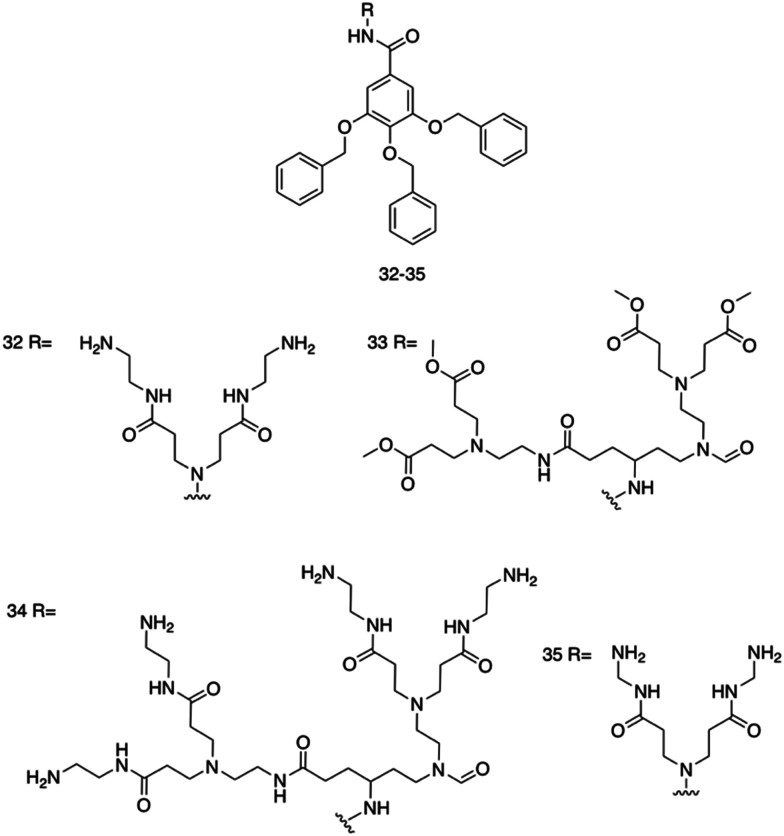
Compounds 32–35 developed by Kannan *et al.*^241^

Four poly(aryl ether) based supramolecular amphiphilic dendrimers, 35–38, (Fig. 15), were synthesized by Kannan *et al.*, and were found to exhibit both hydrogel formation properties and promising broad-spectrum activity against both *E. coli* and *S. aureus*.^241^ The poly(aryl ether) dendron-polyamidoamine (PAMAM) compounds 35 and 36 were synthesised, and were hypothesised to present antimicrobial activity due to their surface charge and amphipathicity. The next-generation compound 37 was then developed to elucidate structural characteristics contributing to the antimicrobial activity. Finally, compound 38 was synthesised, terminating in hydrazide groups to understand the effects of terminal amine group protonation on material and antimicrobial properties.

MGCs were first established for each compound at room temperature in DMSO : water mixtures (2 : 8), revealing MGCs of 6.5 mg mL^−1^ for 35, 0.9 mg mL^−1^ for 36, no gelation was observed for 37 and 5.0 mg mL^−1^ for 38.^242^ Aggregation occurring below the MGC was observed using UV-Vis studies, whereby water was added to DMSO solutions of the compounds, with the resulting blue shifts occurring at the absorbance region assigned to π–π transitions (≈280 nm) monitored. These blue shifts, or hypsochromic shifts, occur due to the different alignment of the transition dipole moments upon π–π interactions, and can therefore be used to confirm π–π interactions.^243^ Blue shifts between 6–18 nm were observed for all four compounds, suggesting π–π interactions involved in the self-assembly process, indicative of H-aggregates. Following this, SEM and dynamic light scattering (DLS) experiments in DMSO : water (1 : 9 v/v) mixtures, revealed spherical aggregates, with hydrodynamic diameters between 200–300 nm. Additionally, aggregate stability in both PBS and biological media (DMEN with 15% FBS) was monitored. Compounds 35, 36 and 37 displayed minimal aggregation over 72 hours, while compound 38 formed large visible aggregates. In biological media, the aggregate size of compounds 35 and 36 was monitored over time, with no significant deviation from their aggregation behaviour in PBS, indicating no specific protein interactions. Zeta potential measurements at physiological pH (7.4) revealed positive surface potentials for compounds 35 and 37, while 36 and 38 presented negative surface potentials, with the authors attributing these differences to the different protonation states of the amines and hydrazides at physiological pH. The structural features of each compound, specifically the lipophilicity and p*K*_a_, were then calculated at pH 7.4 using AlogPs and Marvin sketch software (18.3.0). Calculated p*K*_a_ values corresponded to the observed findings in the zeta potential investigations, while the calculated log*P* (*c*log*P*) value of the molecules revealed a decrease in the amine functional groups between compound 37 (1.07) and 35 (3.05) resulted in higher *c*log*P* values. Compounds 36 (4.29) and 38 (3.99) both displayed higher *c*log*P* values than 35 and 37. Log*P* is used as a measure of hydrophobicity of a molecule, and has been correlated with membrane integration and permeability, and is therefore an important parameter to consider when designing antimicrobials.

The antimicrobial activity of compounds 35–38 was established against *E. coli* and *S. aureus* using a modified broth microdilution method. Resulting MIC and minimum bactericidal concentration (MBC^244^) measurements revealed compound 35 to display the most potent antimicrobial activity, with MICs and MBCs of 0.062 mg mL^−1^ and 0.125 mg mL^−1^ obtained respectively against *E. coli* and a value of 0.031 mg mL^−1^ obtained for both the MIC and MBC against *S. aureus*. Zeta potential measurements of both *E. coli* (−20 mV) and *S. aureus* (−12 mV) before and after increasing additions of compound 35 revealed a decline in zeta potential with increasing concentrations of 35 (From −20 mV to −5 mV for *E. coli* and −12 mV to 0 mV for *S. aureus*), suggesting strong electrostatic interaction with both membranes. To further validate bacterial membrane targeting, the carboxyfluorescein diacetate succinimidyl ester (cFDA-SE) leakage assay (increasing fluorescence correlates with membrane disruption) and propidium iodide (PI) uptake assay (only stains dead cells)^245^ were performed, both of which showed increased fluorescence intensity upon addition of 35, confirming membrane targeting. An additional membrane depolarization assay was conducted using 3,3′-dipropylthiadicarbocyanine iodide (DiSC_3_(5)), a membrane potential dye that shows no fluorescence when accumulated at the membrane of energized cells. After treatment with compound 35, increased fluorescence was observed compared to the non-treated control cells for both *E. coli* and *S. aureus*, revealing depolarization of the membrane induced by compound 35. SEM images of both *E. coli* and *S. aureus* after treatment with compound 35 at its MIC displayed spherical aggregates surrounding the cells, attributed to the self-assembled spherical aggregates previously shown to form, with disintegrated bacteria presenting distinct changes in morphology after 12 hours. Cumulatively, these results clearly indicate compound 35 effectively targets the bacterial membranes, inducing membrane disruption. Finally, cytotoxicity studies conducted on fibroblast cells (NIH/3T3) revealed no significant cytotoxicity of 35 up to a concentration four times that of the MIC.

### 3.2. Self-assembling macromolecular antimicrobials

#### 3.2.1. Advantages and disadvantages of macromolecules

Macromolecules unify two key properties of AMPs and small molecules. Macromolecules, by definition, entail large structures, enabling sizes to be achieved similar to AMPs. These larger structures enable variability in charge, amphipathicity and hydrophobicity to be achieved through simple alterations to the chemical structure,^246^ all of which are fundamental to self-assembling AMPs bacterial targeting mechanism of action as described in the self-assembling peptide section. The PK and biodistribution of macromolecules can also be controlled through molecular size and shape.^247^ Taken together, a great degree of tailorability of both the membrane targeting of macromolecules as well as their key therapeutic properties is possible through simple changes to the macromoleculer construct, as a consequence of molecular size. Furthermore, through modification of the active moiety (such as charged groups) density per chain, control of macromolecule repulsive or attractive interactions are possible at nanometre distances.^246^ Additionally, unlike small molecules, macromolecules display non-volatilization, an inability to permeate through the skin, as well as longer circulatory times and reduced residual toxicity to the environment.^248^

While the size of macromolecules enables several of the benefits also associated with AMPs to be incorporated, the synthetic element of macromolecules also instils multiple benefits that are more commonly associated with small molecules. Firstly, AMPs often require expensive methods of extraction and synthesis, costing between $50–400 per gram,^249,250^ while macromolecule synthesis can often be achieved using fewer steps, requiring significantly lower costs.^189^ Commodity polymers for example offer massive scale production with over 100 million tonnes of polyethylene produced annually,^251^ while also retaining small cost per unit volumes compared to similar property containing materials.^252^ AMPs are also plagued by proteolytic degradation, low bioavailability and toxicity issues.^253^ Macromolecules can also avoid incorporation of peptide bonds, the target of proteases, preventing proteolytic degradation.^254^

Despite the promise self-assembling macromolecules have demonstrated as antimicrobial agents, clear shortcomings are still present which prevent such technologies achieving their pharmaceutical potential. To date, synthetic antimicrobial polymeric materials often present higher cytotoxic levels compared to natural AMPs.^189^ This remains a barrier to current clinical utility. Secondly, the increased size of these molecules removes these compounds from well-established drug-like molecule guidelines.^255,256^ Lead-optimization processes are not as thoroughly investigated in macromolecular systems as they are with small molecule systems, and as such may require greater time and financial investments for successful drug candidate development. Processes such as transport within a system need considerably more investigation, with design processes generally presenting a decrease in high-throughput methodology.^257^

#### 3.2.2. Current self-assembling macromolecules under investigation

##### 3.2.2.1. Synthetic monomeric units

**Fig. 16:**
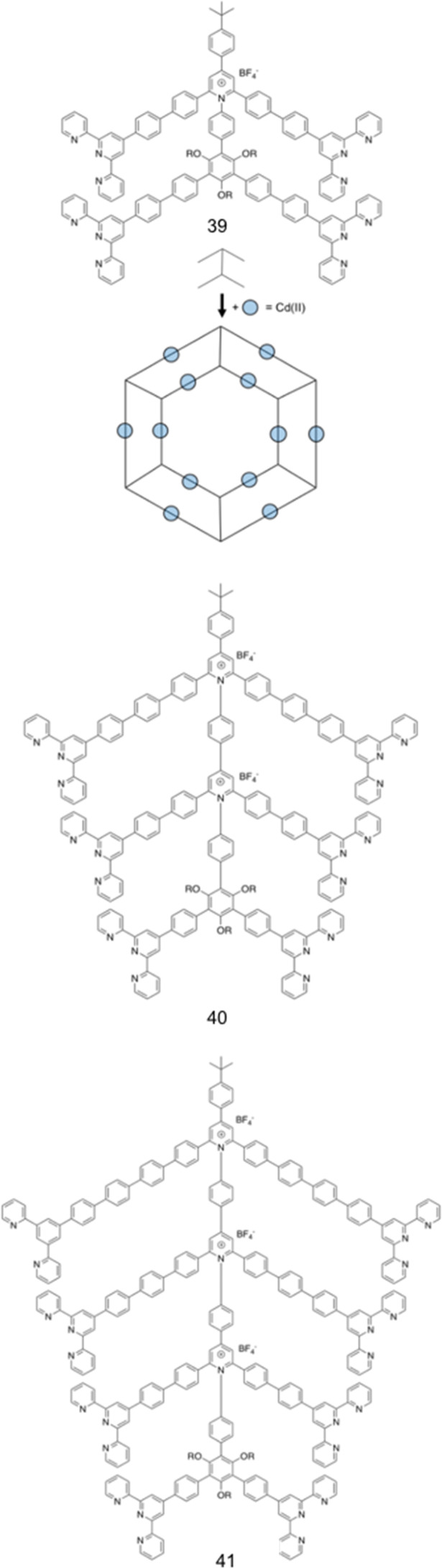
Compounds 39–41 and the resulting Kandinsky circle formed from compound 39 with Cd(ii).^258^

Supramolecular Kandinsky circles consist of giant nested hexagonal structures, illustrated in Fig. 16. Here, three generations of 2D multi-layered concentric, coordination driven Kandinsky circles demonstrating antibacterial activity comparable to daptomycin against MRSA were developed by Wang *et al.*^258^ The multitpic terpyridine (tpy) ligands, compounds 39–41 (Fig. 16) were synthesised through sequential condensation and deprotection of isolated ditopic tpy pyrylium salts. Self-assembly of 39–41 was achieved through metal ion coordination with Cd(ii) at 1 : 2 (39 : Cd(ii)), 1 : 3 (40 : Cd(ii)) and 1 : 4 (41 : Cd(ii)) ratios, resulting in the formation of each compounds respective supramolecular Kandinsky circle. Compound 39 and its corresponding suprastructure is shown in Fig. 16. After successful formation of the suprastructures, electrospray ionisation (ESI) mass spectrometry (MS) was used to identify the hexamers with molecular weight of 17, 28 and 38 kDa for the Kandinsky circles of compounds 39–41 respectively. Travelling wave ion mobility-mass spectrometry (TWIM-MS) confirmed only one conformer existed for each structure. Proton NMR performed on the Kandisky circle formed from compound 39 indicated the formation of highly symmetrical structures, and 2D-DOSY NMR experiments in combination with TEM images corroborated the diameters of the three superstructures for compounds 39–41 calculated through molecular modelling (8.0 nm, 10.2 nm and 11.4 nm for 39–41 respectively). TEM further confirmed the presence of uniform structures, while scanning tunnelling microscopy (STM), a powerful technique for imaging the topographic structure of surfaces, observed nanoribbon-like structures occurring through hierarchical self-assembly of the nested hexagons.^259^ Slow diffusion of diisopropyl ether into DMF solutions of compound 39–41 Kandinsky circles induced nanofiber formation, captured with TEM imaging, achieving lengths up to several μm and diameters of ≈5 nm.

The antimicrobial activity of self-assembled compound 39–41 Kandinsky circles was proposed for two reasons: firstly, the authors theorised the resulting high-charge of the nested hexagonal suprastructures would electrostatically interact with anionic glycopolymers, such as teichoic acids and lipoteichoic acids (Fig. 3b), present within bacterial membranes.^260,261^ Secondly, the incorporation of pyridinium polymers and metal complexes were both hypothesised to instil antimicrobial properties upon membrane binding.^262,263^ Initial experiments established the MIC of compound 39–41 Kandinsky circles against MRSA (ATCC 33591) and *E. coli*. The MIC values obtained for compound 39–41 Kandinsky circles against MRSA were 3 μg mL^−1^, 0.5 μg mL^−1^ and 0.5 μg mL^−1^ respectively. Interestingly, these values are comparable to that of daptomycin, the first clinically approved lipopeptide antibiotic identified to act against MRSA.^264^ However, no antimicrobial activity was observed for these same compounds against *E. coli*, which the authors suggested was due to the presence of both the inner and outer membranes of the Gram-negative *E. coli* blocking penetration of the antimicrobial agent. Following the observed antimicrobial activity, haemolysis was probed with negligible toxicity observed against RBCs. To elucidate the mechanism of action by which antimicrobial activity was achieved, three experiments were performed. Firstly, conductance measurements using planar lipid bilayers consisting of the two most abundant phospholipids in bacterial membranes, DPPG/DPPE, were recorded, with the results suggesting the nested hexagons could form transmembrane channels. Secondly, the subcellular localization of compound 39–41 assembled Kandinsky circles was monitored using 3D deconvolution fluorescence microscopy. This was achieved using FM4-64 dye to visualise bacterial membranes, which emits at 640 nm, and capturing the 39 and 40 Kandinsky circles intrinsic emission maxima of 510 nm and 525 nm respectively. The resulting fluorescence revealed compound 39 and 40 on the surface of Gram-positive *S. aureus* but not on the Gram-negative *E. coli*. Thirdly, TEM imaging on compound 39–41 Kandinsky circles treated MRSA showed cell envelope damage and cell lysis. The resulting images also showed an empty space in the cytosol in addition to cytoplasmic leakage. These findings led authors to hypothesise that the cationic supramolecules absorbed onto the anionic glycopolymers, with resultant cellular damage occurring due to the stacking of the supramolecules into transmembrane channels within the inner lipid membrane. This would occur *via* π–π interaction of the backbone, van der Waals interactions and hydrophobic interactions between the supramolecules and lipids within the membrane.

**Fig. 17:**
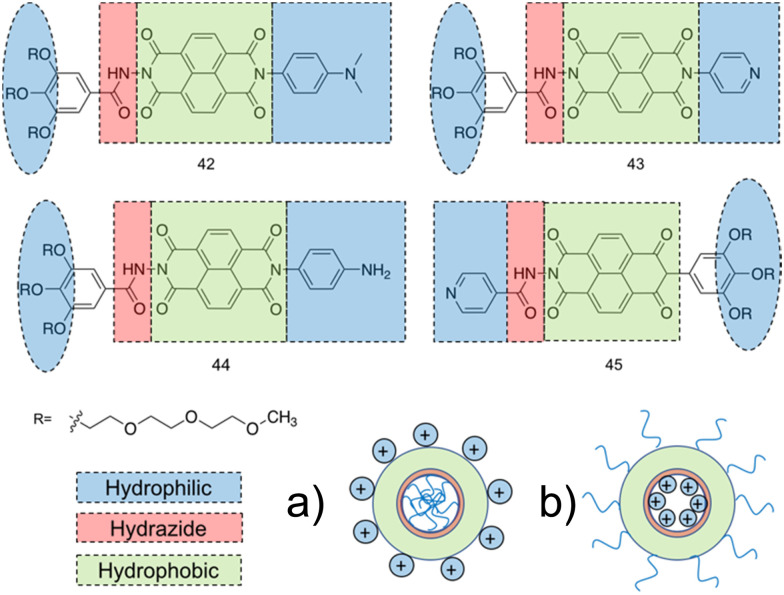
Compounds 42–45 synthesised by Sikder *et al.*^267^ The two hydrophilic ends of the molecule (blue), the hydrazide group (red) and the hydrophobic π-conjugated core (green). (a) The resulting polymersome from compounds 42–44. (b) The resulting polymersome from compound 45.

Amphipathicity is a key component of AMPs required for eliciting antimicrobial action, as discussed in the self-assembling peptide section. In the following study, π-amphiphilic macromolecules, (compounds containing a hydrophobic π-conjugated core) were synthesised and shown to elicit potent broad spectrum antibacterial activity. Sikder *et al.* developed a series of π-amphiphiles containing the π-conjugated hydrophobic naphthalene diimide (NDI) centred chromophores at their core, with varying hydrophilic wedge/functional groups attached to opposite ends of the molecular construct (Fig. 17).^265,266^ The resulting π-amphiphilic compounds, 42–45, were shown to self-assemble into polymersomes. Here, a single hydrazide group was introduced to the molecular construct causing unilateral π-stacking, fixed through hydrogen bonding between the neighbouring hydrazide groups (Fig. 17). For compounds 42, 43 and 44 (Fig. 17), placing the amine group on the opposing hydrophilic side of the molecule to the hydrazide functionality allowed these amine groups to position at the surface of the resulting polymersomes. The amine incorporated into the hydrophilic moiety was varied for each compound in order to determine which design presented the optimum antimicrobial activity (Fig. 17).^267^ The aforementioned structure was reasoned to enable strong multivalent interaction of the polymersome with the anionic bacterial membranes due to the unilateral presentation of positively charged amine groups at its surface, while the hydrophobic NDI stack would lead to further membrane disruption as previously demonstrated for antimicrobial polymers.^268^ In compound 45 (Fig. 17) the amine group (the same amine present within compound 43) was introduced into the same hydrophilic portion of the molecular structure as the hydrazide functionality, which resulted in the amine groups positioning on the inner surface of the polymersome. The self-assembly of the polymersomes for each compound was confirmed with TEM imaging, observing hollow spherical structures, while UV-Vis supported aromatic interaction of the NDI centred chromophores, confirming π-stacking within the molecular self-assembly processes. DLS experiments showed hydrodynamic diameter averages to vary between 110 nm and 460 nm for the resulting polymersomes of compounds 42–45. Critical aggregation concentrations (CACs), the minimum concentration at which aggregation occurs, were measured for the four compounds, with compound 42 giving the lowest CAC (0.007 mM). Compounds 43 and 45 differed in CAC from each other by 2 orders of magnitude, revealing 43 presents a much greater propensity to undergo self-association processes. Furthermore, compound 43 registered a higher zeta-potential of +12.5 mV compared to 45, which exhibited a zeta potential of 0 mV, inferring a greater degree of aggregate stability at the concentration studied. The instability of compound 45 polymersomes suggested pyridine groups were displayed in the inner walls of the polymersomes as hypothesised, resulting in electrostatic repulsion between the head groups due to their close proximity.

To investigate the ability of compounds 42–45 to interact with bacteria, isothermal titration calorimetry (ITC) was conducted on the four compounds against DPPG/DPPE (88 : 12) and DPPC lipid vesicles, allowing for the thermodynamic properties of any interactions to be quantified.^269^ Previous experiments established DPPG/DPPE (88 : 12) and DPPC lipid vesicles as good models for bacteria and mammalian cell membranes respectively.^270^ Within the scope of these experiments, the synthetic vesicles were injected into a solution of compounds 42–45 at a concentration above the CAC. Compounds 42–44 all presented high specificity towards the bacterial membrane model, with compound 42 displaying the strongest interaction, while no interaction was observed with the mammalian membrane model. Compound 45 displayed no interaction with either membrane, confirming the positioning of amine groups on the inside of the polymersomes directed by the hydrogen bonding of the hydrazide directly influenced the multivalent binding to bacterial membranes. Following this, the antimicrobial properties of the supramolecular assemblies were screened against *E. coli* (ATCC-25922) and *S. aureus* (ATCC-25923). MIC values were obtained for the four compounds, with 42 displaying the most potent MIC values of 62.0 μg mL^−1^ and 15.8 μg mL^−1^ for *E. coli* and *S. aureus* respectively. These results correlated with the liposome binding studies, in which compound 42 displayed the strongest binding affinity among compounds 42–45, suggesting a correlation between thermodynamics of binding and antimicrobial activity. The resulting lower MIC value for the Gram-negative *E. coli* was of particular interest due to the documented increased difficulty in killing Gram-negative as opposed to Gram-positive bacteria. This broad-spectrum activity was hypothesised to be due to the enhanced hydrophobicity of compound 42 compared to compounds 43–45 due to the headgroup of 42 containing two methyl groups. Alongside the enhanced efficacy of 42, 43 also displayed a low MIC value of 29.4 μg mL^−1^ against *S. aureus*. Time kill assays conducted against *S. aureus*, showed 42–44 caused a 5 log reduction in *S. aureus* within 250 minutes of treatment. The mechanism by which compound 42 achieved its antimicrobial activity was investigated utilising a live/dead assay. Here, the green dye SYTO 9 which can internalize within both live and dead cells, and a red emitting PI dye which can only cross damaged membranes were used to determine the integrity of the bacterial membrane after treatment with compound 42 using fluorescence microscopy.^271^ After treatment with compound 42 both *S. aureus* and *E. coli* presented prominent red channel emission, suggesting 42 follows the membrane disruption pathway. Finally, haemolysis studies using RBC cells showed high selectivity of compounds 42, 43 and 45 towards bacteria, whereas 44 exhibited high haemolytic activity. Together, these data indicated compound 42 as the lead candidate towards antimicrobial development, demonstrating a selectivity of >40 and >157 fold for *S. aureus* and *E. coli* respectively when compared to human RBCs.

##### 3.2.2.2. Polysaccharides

Chitosan is a widely investigated natural antimicrobial agent found in the shells of crustaceans.^272^ The interest in this polymer centres around its broad spectrum antimicrobial effects and high commercial potential as a result of its versitility.^273^ Studies have been conducted to improve chitosan's antimicrobial activity through increasing the number of positively charged groups present within the molecular structure, highlighting how careful design can enhance this polysaccharides antimicrobial action.^274^ These derivatives include carboxymethyl chitosan and chitosan-*N*-arginine, which also offer increased water solubility, an important factor when considering the clinical application of these polymers.^275^

**Fig. 18:**
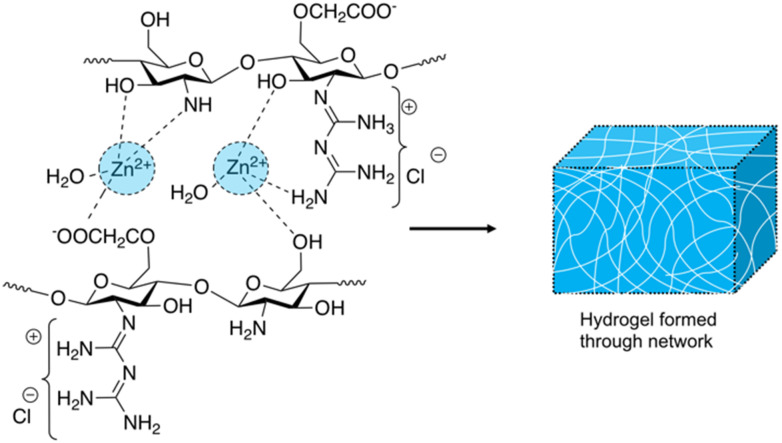
The proposed network formation produced through Zn^2+^ crosslinked compound 47 resulting in hydrogel formation.^276^

One such chitosan derivative incorporated into a supramolecular structure was prepared by Salama *et al.*^276^ Here the authors hoped to improve upon the antimicrobial properties of *O*-carboxymethyl chitosan, 46, through guanidinylation, producing *O*-carboxymethyl chitosan biguanidine hydrochloride, 47. Encapsulation of 47 into a hydrogel was achieved through zinc crosslinking, using 2% (2Zn), 4% (4Zn) and 6% (6Zn) w/v Zn^2+^ ions, introduced as a zinc nitrate solution. Compound 46/2Zn was also utilised as a control. The resulting zinc-47 complex is shown in Fig. 18. Successful zinc crosslinking was confirmed using FTIR; band intensity at 1595 cm^−1^ (–NH_2_ bending) was observed to decrease, whilst the band at 3444 cm^−1^ was shown to broaden in 47/6Zn, compared to that of compound 47 alone. These changes were hypothesised to be due to the chelation between Zn^2+^ and the –OH and –NH_2_ groups (Fig. 18). SEM images of the compound 47 hydrogel showed a porous surface, with the increase in Zn^2+^ causing a decrease in pore size, hypothesised to be due to increased crosslinking. Interestingly, X-ray diffraction for the compound 47/6Zn hydrogel presented an additional diffraction peak compared to 46 and 47 controls, potentially due to the formation of ordered regions resulting from chains of 47 and Zn^2+^ ions.^277^ Thermal stability testing also showed enhanced thermal stability with increasing concentration of Zn^2+^ ions.

The antimicrobial activity of hydrogels formed from compound 47/Zn was established through zone of inhibition studies against *E. coli*, *B. subtilis* and *Streptococcus pneumonia* (*S. pneumonia*). The antimicrobial activity of the chitosan hydrogels were compared against ampicillin and gentamicin standards. Firstly, the effect of guanidinylation of compound 46 was elucidated *via* comparison of 46/2Zn to 47/2Zn. The resulting zones of inhibition confirmed 47/2Zn significantly enhanced the antimicrobial effects compared to the non- guanidylated sample, 46/2Zn. Authors attributed the enhanced efficacy to the high number of positive charges provided by the presence of biguanidinium groups. These biguanidinium groups allow interaction with, and disruption of, the passage of negatively charged components such as proteins, phospholipids and fatty acids through the bacteria's cell wall, accelerating cell death.^278,279^ Furthermore, the antimicrobial activity of the hydrogels formed from compound 47/Zn increased with the increasing concentration of zinc ions. This activity culminated in the compound 47/6Zn hydrogels achieving zones of inhibition within ≈1 mm of ampicillin against *B. Subtilis* and *S. pneumonia* and, gentamicin against *E. coli*. The increased concentration of zinc ions pertained within the hydrogel can chelate to the negatively charged components of the membrane, causing leakage and a loss of proton motive force, a process that creates an electrochemical gradient in the cell and is a vital requirement for ATP production. Additionally, Zn^2+^ is known to generate reactive oxygen species (ROS) leading to cell wall damage.^280–282^ Cytotoxicity tests revealed any toxicity witnessed from the hydrogels resulted from the zinc ions, with compound 47 giving 100% cell viability at all of the concentrations used for zone of inhibition trials. IC_50_ values for the MCF-7 breast cancer cell line for solutions of zinc nitrate was calculated to be 244 μg mL^−1^, whilst Zn^2+^ concentrations released by the 47/Zn hydrogels were 57, 90 and 110 μg mL^−1^ for 47/2Zn, 47/4Zn and 47/6Zn respectively. Therefore, even in the 47/6Zn hydrogels, ∼90% cell viability was observed.

**Fig. 19:**
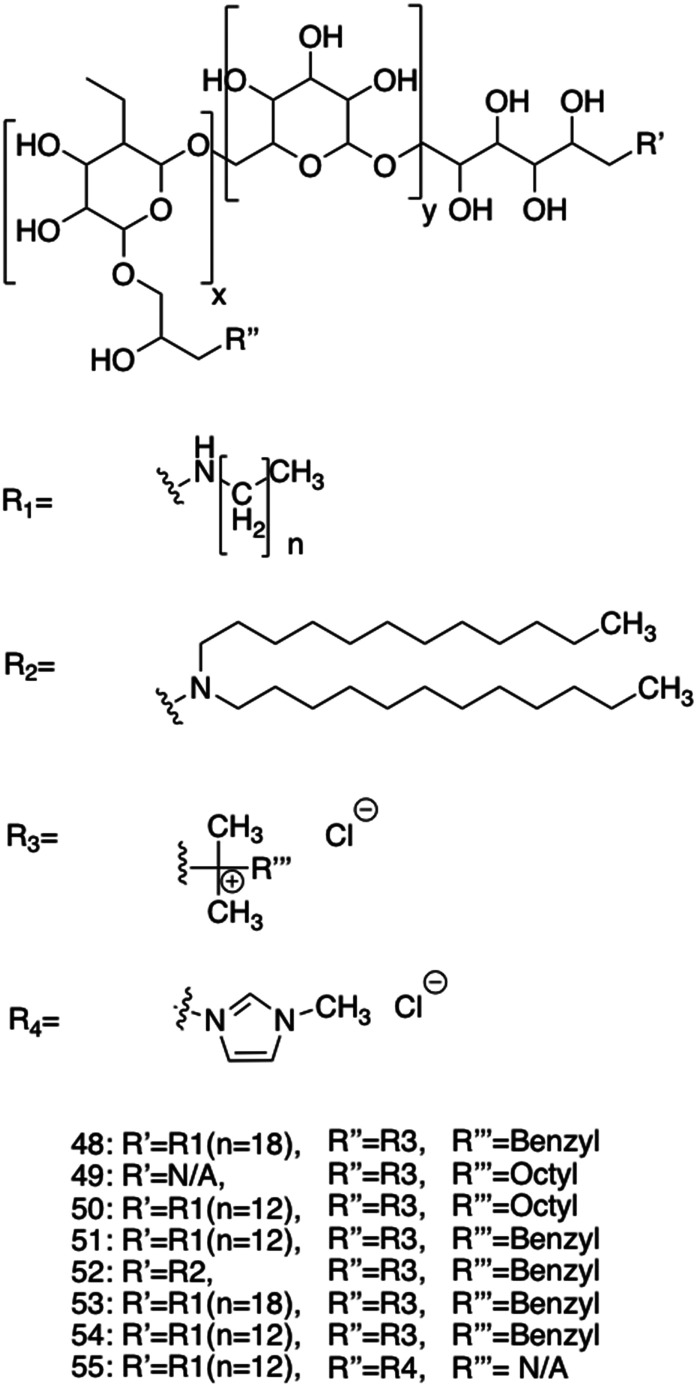
The structure of compounds 48–55.^286^ The first R_1_ group was varied between C_12_ and C_18_. The R_3_ group on the first quaternary ammonium R_2_ group was varied between benzyl and octyl groups.

Dextran, a glucose derived natural biodegradable polysaccharide, has been extensively utilised to create a range of antimicrobial materials.^283–285^ An investigation conducted by Tuchilus *et al.* used dextran (Either 8000 or 4500 average molar masses) as a backbone to create a self-assembling amphiphile achieving potent antimicrobial activity against MRSA.^286^ In this study quaternary pendant ammonium groups were attached to the dextran backbones, while varied length alkyl chains were incorporated at the reductive end of the polysaccharide, producing a series of dextran derivatives, 48–55 (Fig. 19). The aforementioned polymer was designed to encompass the greatest charge density attainable, whilst still preserving the amphiphilic nature to allow for self-assembly in aqueous solution.^287^ This compromise was achieved by keeping the content of quaternary pendant ammonium groups at 30 mol%. A library of compounds based on this design, 48–55 were synthesised (Fig. 19). Benzyl and octyl groups were chosen for attachment at the R_3_ position due to their moderate hydrophobicity, with previous studies utilising longer or shorter alkyl chains presenting no antibacterial properties.^288,289^ Investigation into the self-assembly of the resulting compounds, revealed CACs between 0.83–3.00 mg mL^−1^ for 48–55, with all resulting aggregate structures measuring zeta potentials of +26 mV or higher, indicating good stability. TEM images showed formation of spherical micelles ranging between 10–20 nm in diameter in the dry state, while DLS obtained larger measurements of 150–300 nm in the solution state, due to extension of the charged dextran chains by intramolecular electrostatic repulsion in the hydrated state.

Zone of inhibition studies were used to probe for antimicrobial activity, with the diameters of the zone of inhibitions used to compare activity between the compounds. Seven species of bacteria or fungi were used: *S. aureus* (ATCC 25923), *Micrococcus lutea* (*S. lutea*) (ATCC 9341), *E. coli* (ATCC 25922), *P. aeruginosa* (ATCC 27853), *Candida albicans* (*C. albicans*) (ATCC 90028), *Candida galbrata* (*C. galbrata*) (ATCC MYA 2950) and *Candida parapsilosis* (*C. parapsilosis*) (ATCC 22019). In these zone of inhibition studies, the full library of compounds 48–55 displayed the greatest antimicrobial activity towards the fungus *C. parapsilosis* and the bacteria *S. aureus*, whilst the lowest efficacy was observed against *E. coli*, with only compounds 49, 50 and 54 displaying any activity against this bacteria. The authors noted similar patterns are observed among analogous quaternary ammonium compounds, with Gram-positive organisms displaying a greater degree of susceptibility to this class of compound.^290^

From the resulting zone of inhibition diameters, it was deduced that the ratio between hydrophilic and hydrophobic parts of the dextran molecule, altered *via* dextran's molecular mass (8000 molar masses with 2–3% wt% content or 4500 molar masses with 3.6–5.3 wt% in the hydrophobic block), had a decisive effect on the activity of the compound. Specifically, a higher percentage weight in the hydrophobic block corresponded to an increase in activity. Additionally, compounds in which R_3_ was a benzyl group displayed the greatest activity, while the length of the alkyl chains had no significant effect on the activity of the compounds (48–52 and 53–54) with the same dextran sample. Following these zone of inhibition studies, MICs and MBCs were obtained for compounds 48–54 against *S. aureus* (ATCC 25923) and *C. albicans* (ATCC 90028) and compared against control drugs of ciprofloxacin and fluconazol. For *S. aureus*, compounds 53 and 54 presented the lowest MIC and MBC values (0.06 mg mL^−1^ and 1.25 mg mL^−1^ respectively), with these MIC and MBC values lower than that of the ciprofloxacin control (1 mg mL^−1^ and 2 mg mL^−1^ respectively). However, due to the MBC equalling 20 times that of the MIC it was suggested these compounds displayed mainly bacteriostatic activity, with the bactericidal activity cut off traditionally set as less than four times the MIC.^291^ Similarly, compounds 53 and 54 also presented the lowest MIC and MBC values against *C. albicans* (1.25 mg mL^−1^ and 2.5 mg mL^−1^ respectively), once again lower than the fluconazole control (8 mg mL^−1^ and 16 mg mL^−1^ respectively). Interestingly compounds 48–52 also presented MIC and MBC values lower than that of the control against *C. albicans* (2.5 mg mL^−1^ and 5 mg mL^−1^ respectively). It was noted that compounds 48–52 MICs were similar to the CAC value, suggesting self-assembly may be important to the antimicrobial activity, whilst for compounds 53 and 54 MICs much lower than the CAC were observed. Further MIC and MBC values were then obtained against clinical pathogenic strains of *S. aureus*, this time revealing MIC values for compound 53 of 1.25 mg mL^−1^ against *S. aureus* (65) and 2.5 mg mL^−1^ against *S. aureus* (100), and an MIC of 1.25 mg mL^−1^ for compound 53 against both *S. aureus* (65) and *S. aureus* (100). In these instances, the MBC values obtained for both compounds 53 and 54 against both *S. aureus* strains were either equal to or twice that of the MIC, suggesting a bactericidal mode of action. Together these results show potential for these dextran derivatives as antimicrobials against a broad range of microorganisms.

## 4. Nanoparticles (NPs) and nanopatterned materials as antimicrobial agents

Here we highlight those nanomaterials which have been developed as antimicrobial alternatives, with a focus on NPs and nanopatterned materials (NPMs). Such NPs and NPMs are of great interest for development within the field of antimicrobial development due to an inherently large surface area and ability to reach high local concentrations, while exhibiting low systemic concentrations. This effectively enhances a desired biological activity in comparison to the corresponding free molecule or larger condensed aggregated species.^105,292^ These advantages have led to a vast amount of interest in this research area, and in turn the publication of a number of reviews.^293–295^

### 4.1. NPs

NPs are defined, for the purpose of this review, as solid particles under 100 nm in size, in all dimensions.^296,297^ Depending on the molecular constituents used to form the NPs, examples can be further classified as either organic or inorganic.^298^ In addition, different NPs often display a combination of unique properties both, physically (*e.g.* mechanically),^299^ chemically (*e.g.* electronic)^300^ and biologically (enhanced antimicrobial efficacy),^301^ compared to that of the bulk material.^302,303^ Broad spectrum antibacterial properties have been demonstrated against both Gram-positive and Gram-negative bacteria, with varied mechanisms of action including the utilisation of oxidative stress and metal ion release from the NP itself.^294,304^ Antimicrobial efficacy for this class of materials can also be further tailored through alteration of the NPs physical size and shape.^301,305^

#### 4.1.1. NPs mechanisms of action

Described below are two generic categories (oxidative and non-oxidative/metal ion release mechanisms) by which NPs have been shown to elicit antimicrobial efficacy.

##### 4.1.1.1. Oxidative stress

Oxidative stress refers to a state in which a cell's antioxidant defence mechanisms can no longer neutralise the increased concentrations of intracellular ROS.^306^ This results in a cascade of intracellular damage, including the breakage of DNA, peroxidation of lipids and protein modifications.^307,308^ NP induced oxidative stress has been well documented in a multitude of studies. Both organic and inorganic NPs display this mechanism. For example, Akhtar *et al.* demonstrated ROS-mediated cytotoxicity from organic silica NPs,^309^ while Quinteros *et al.* showed inorganic silver NPs generated oxidative stress in *S. aureus*, *E. coli* and *P. aeruginosa*, with the increase in ROS correlating with increased antimicrobial activity.^309,310^ Three chemical factors responsible for the induction of oxidative stress within bacteria are: the presence of reactive surface pro-oxidant functional groups, active redox cycling from transition metal-based NPs and physical NP:cell interactions.^311^ When considering the incorporation of reactive surface functional groups, several successful methods have been identified.^311^ These include the incorporation of reactive particle surface groups, such as surface bound radicals, including silicon oxide and silicon dioxide, to the surface of quartz scaffolds resulting in NPs that act as active centres with the ability to produce ROS.^312^ These surface radicals subsequently react with water and oxygen to generate hydroxyl radicals which elicit the antimicrobial effect.^313^ In a further example, adsorption of ozone and nitrogen dioxide onto the surface of NPs has also been shown to result in ROS generation due to the strong oxidant nature of these gases.^314,315^ This process forms a film of the absorbate onto the NP which enables these surface bound oxidants to induce oxidative damage to the microbes.^105^ The second chemical factor involves the utilisation of transition metal redox cycling processes as exemplified by the copper oxide based NPs developed by Meghana *et al.*^316^ Here, when bacteria are exposed to copper based NPs, copper(ii) is reduced to copper(i) *via* thiol groups found in the biological molecules present within the bacteria.^316^ This subsequently produces superoxide species, leading to oxidative stress and the halting of the cell cycle. Particle cell interactions, the final chemical factor identified, refers to interactions of the NP with components of the bacterial cell itself. Interactions that are often observed include NP induced ribosomal damage, and modifications of proteins/enzymes/DNA.^317^ Importantly, whilst ROS are effective at stopping bacterial growth, they can also be undesirable if non-selectively induced, incurring cytotoxicity in human cells, as observed with silver NPs.^318^ When the production of ROS are induced in human cells this can also initiate oxidative stress, leading to lipid peroxidation, mitochondrial toxicity, protein damage (including damage to enzymes) and DNA/RNA damage.^319–322^

##### 4.1.1.2. Non-oxidative mechanisms and metal-ion release

The following section highlights several other key mechanisms by which NPs induce antimicrobial activity, that do not fit into the category of oxidative stress.

Destabilization of the membrane is one such alternative mechanism. This is often achieved through electrostatic binding of NPs to bacterial cell walls and biological membranes in a similar manner to that discussed for AMPs, causing a change in membrane potential, commonly resulting in membrane depolarization.^323^ Membrane potential refers to the electrochemical gradient created between the internal and external environments of the cell due to the difference in ion concentration across the membrane.^324^ This membrane potential is vital for the cell to perform many of its functions.^325^ Therefore, through the controlled manipulation of NP:cell binding events, the resultant bacterial membrane destabilisation can result in compromised membrane transport events enabling an uncontrolled influx of molecules,^326^ compromised respiration and energy transduction,^327^ as well as cellular osmotic damage.^328^ Each of these effects can lead to cell death.

Another possible mechanism of action, exclusive to inorganic NPs, is through metal oxide NPs releasing metal ions that can penetrate bacterial cell membranes.^329^ Upon entering the bacterial cell cytoplasm, metal ions can interact with various functional groups contained within biological macromolecules such as proteins and DNA, resulting in bactericidal activity through multiple modes of action.^329,330^

One example which combines both of these mechanisms of antibacterial action, produced by Lueng *et al.*, investigated the use of magnesium oxide NPs as antimicrobial agents against *E. coli*. During this investigation, electron microscopy and proteomics data supported the mechanism of antimicrobial action to include bacterial membrane damage. Evidence gained through attenuated total reflectance (ATR) FTIR revealed NP-bacteria interaction, with the authors suggesting this NP-bacteria interaction in addition to magnesium ion release and potential pH changes to be the cause of the observed membrane damage, which resulted in cell death.^331^

#### 4.1.2. Current research in inorganic NPs

##### 4.1.2.1. Silver NPs

Silver NPs are well established as effective antimicrobials.^332^ Of all inorganic NPs available, silver NPs frequently present amongst the most effective antibacterial NPs reported,^333^ although this is often paired with undesirable toxicity and bio-accumulation.^330^ Furthermore, the development of bacterial resistance to silver NPs has been reported, highlighting the importance for reserving this technology to focussed areas such as healthcare settings over alternative industrial and household applications.^334,335^ Although not fully elucidated, multiple mechanisms of action have been proposed for silver NPs. These include the previously covered metal ion release mechanism, by which silver ions release is hypothesised to result in the increased permeability of the cell wall and membrane leading to disruption, in addition to metal ion interference with biological machinery, DNA replication and ATP synthesis. Furthermore, silver ions are also known to produce ROS, while the NPs themselves are thought to be able to perforate the bacterial cell membranes, resulting in leakage.^336^

One such silver NP investigated by Loo *et al.* was found to be effective against a variety of foodborne pathogens, which impact ≈30% of industrialised country populations each year such as through contraction of foodborne illness.^337,338^ The spherical silver NPs produced were determined to have a diameter of 4.06 nm by TEM. Elucidation of antimicrobial efficacy was achieved through determination of the relevant MIC values against four different species of Gram-negative bacteria, all known to be foodborne pathogens. Here an MIC of 3.9 μg mL^−1^ was established for *Klebsiella pneumoniae* (*K. pneumoniae*) (ATCC 13773), *S. typhimurium* (ATCC 14028) and *Salmonella enteritidis* (*S. enteritidis*) (ATCC 13076), while *E. coli* (ATCC 25922) exhibited an MIC of 7.8 μg mL^−1^. The MBC was then obtained by plating the MIC cultures onto Mueller–Hinton agar and observing the growth of any bacterial colonies after 24 hours incubation at 37 °C. The MBC was found to equal the MIC for *K. pneumoniae*, *E. coli* and *S. enteritidis* however, the MBC obtained for *S. typhimurium* was determined to be twice that of the MIC (7.8 μg mL^−1^ and 3.9 μg mL^−1^ respectively). Time kill assays confirmed that the bactericidal end point for *E. coli* was reached after a two-hour incubation with a NP concentration at four times the MIC. *K. pneumoniae* and *S. enteritidis* both required incubation for two hours at a NP concentration of two times the MIC, while *S. typhimurium* only required incubating for one hour at a NP concentration four times the MIC.

Another investigation, this time into a variant of silver NPs containing lignin polymers, was conducted by Slavin *et al.*, and these were found to be effective against MDR bacteria.^339^ Here lignin was utilised as an environmentally friendly reducing agent in the silver NP synthesis, whilst also offering a second benefit of capping the NP surface with lignin, which offers inherent antimicrobial activity.^340^ By decreasing the w/v solutions of lignin, silver lignin NPs of increasing size were generated.^339^ Increasing the concentration of lignin to 1.0% w/v was found to optimise the formulation, lowering the associated polydispersity index (PDI). Furthermore, the corresponding zeta potential value obtained of this 1.0% w/v NP formulation (−34.2 mV) showed the species formed to be stable in solution. High resolution TEM determined the diameter of these spherical silver lignin NPs to be ≈20 nm. To reveal the resulting structure of the synthesised NPs, initially energy dispersive X-ray was used to confirm the presence of silver. Further investigation using FTIR spectroscopy, indicated the presence of a lignin shell on the outer surface of the silver NPs. MIC values were obtained against a panel of both MDR bacterial strains and reference strains, focusing on a broad range of bacteria including: *S. aureus* (ATCC 700788, ATCC 700788 or MDR strain with resistance to: ciprofloxacin, clindamycin, erythromycin, oxacillin, and penicillin), *S. epidermidis* (MDR strain with resistance to: penicillin, gentamicin, and oxacillin), *P. aeruginosa* (ATCC 33354 or MDR strain with resistance to: amikacin, ceftazidime, ceftizoxime, piperacillin-tazobactam, and tobramycin), *K. pneumoniae* (MDR strain with resistance to: ertapenem and meropenem), *Acinetobacter baumannii* (*A. baumannii*) (ATCC 17961 or MDR strain with resistance to: meropenem) and *E. coli* (ATCC 25922). Silver lignin NPs displayed MIC values between 5–25 μg mL^−1^ against all MDR tested strains, while control silver NPs were only comparably effective against *S. aureus*, *S. epidermidis* and *A. baumannii*, with the other MDR strains needing a concentration over 5-fold higher. Overall silver lignin NPs also displayed a greater degree of efficacy against the MDR strains than the reference strains. Lignin alone was also measured as a control, producing no antimicrobial effects up to 50 μg mL^−1^. These results confirmed that lignin alone, at the concentrations tested, was not responsible for the increased antimicrobial activity over control silver NPs. To elucidate the silver lignin NPs mechanism of antimicrobial action against the bacteria strains, interactions with model membranes were probed. The results indicated the surface activity of lignin influences the PE monolayer, correlating with a cell membrane disruption mechanism of action. Despite the promising aspects of these silver lignin NPs, RNA sequencing revealed the upregulation of several metal efflux pumps, offering the possibility that silver lignin NPs entering bacterial cells could be removed through this mechanism. Thus, resistance may be gained through the upregulation of these efflux pumps.^341^

As previously mentioned, cytotoxicity is one of the primary issues with silver NPs; this study investigated cytotoxicity against three cell lines: THP-1, A549 and fibroblast cells. Both THP-1 and A549 displayed cytotoxicity at a concentration of 25 μg mL^−1^, above these silver NPs MIC against several bacterial species, therefore showing potential for these silver lignin NPs to be used in clinical application. However, fibroblast cells displayed cytotoxicity at a concentration at 1 μg mL^−1^, indicating that careful consideration to the area of application of the treatment would be necessary. Interestingly, the authors observed an increased expression of the anti-inflammatory cytokine IL-10 upon exposure to silver lignin NPs, suggesting potential anti-inflammatory benefits. IL-10 is involved in reducing inflammation during acute infections and tissue injury.^342^ This is important, as many severe complications in infections result from excessive immune activation.^343^ Increased IL-10 activation could therefore act as a secondary potential beneficial property of these antimicrobial silver lignin NPs.

##### 4.1.2.2. Gold NPs

**Fig. 20:**
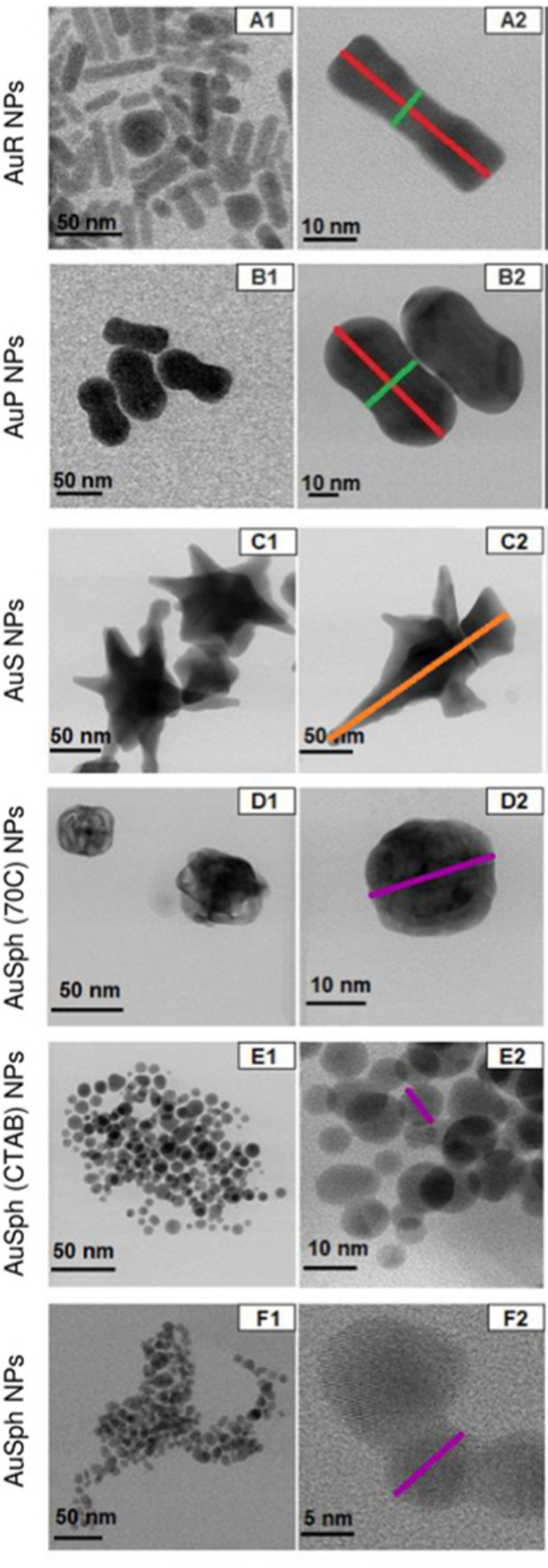
Bright field scanning TEM images of the obtained gold NPs synthesised by Piktel *et al.*, which are described by their shape (rod/peanut/star or sphere) and reaction condition (temperature and/or the addition of centrimonium bromide (CTAB).^301^ (a) AuR NPs–gold rods, (b) AuP NPs–gold peanuts, (c) AuS NPs–gold stars, (d) AuSph (70C) NPs–gold spherical (70C), (e) AuSph (CTAB) NPs–gold spherical, (f) AuSPph NPs–gold spherical NP. This image has been reproduced with the permission of Nature Scientific Reports.

While silver NPs are generally considered to exhibit high levels of antimicrobial efficacy,^333^ gold NPs offer benefits including reduced cytotoxicity whilst still maintaining high levels of antimicrobial activity.^344^ Piktel *et al.* have successfully developed gold NPs of different shapes displaying antimicrobial efficacy at nanogram per millilitre concentrations.^301^ The effective killing concentrations measured in this study were a thousand-fold lower than those reported in previous studies investigating gold NPs.^301,345,346^ This enhancement in activity was achieved through the use of cetrimonium bromide (CTAB) to direct NP shape. Through varying the synthesis reaction time and temperature, four shapes of gold NPs were generated; rods, peanuts, stars and porous spheres. These shapes were identified using scanning TEM and are depicted in Fig. 20. In addition to the porous spherical NP produced *via* these reaction conditions at 70 °C (henceforth referred to as gold spherical 70C), two other spherical control NPs were also synthesised. These control NPs included an unfunctionalized gold spherical NP (no CTAB), and a functionalized gold spherical CTAB NP containing no pores. Selected area electron diffraction revealed all the obtained NPs to be crystalline in structure. Each structure was found to be positively charged between pH 3.5–12.5 however, zeta potential values, and thus NP stability, were found to decrease with increasing pH. Antimicrobial activity for all six gold NPs (rod, peanut, star, porous sphere and two control spheres) were determined against representative isolates of Gram-negative bacteria *E. coli* and *P. aeruginosa*, in addition to the Gram-positive bacteria *S. aureus*. MBC values of 0.078 to 0.625 ng mL^−1^ were obtained for the gold rod, peanut, star and porous sphere (excluding the two control spheres) against *E. coli* and *S. aureus* respectively, while *P. aeruginosa* exhibited a higher MBC 20–40 ng mL^−1^ for those same four gold NPs. Both spherical control NPs were ineffective at doses below 20 μg mL^−1^ against all bacteria tested, showing the major influence shape can have on the antimicrobial efficacy of NPs. Further to this, the use of control gold-spherical CTAB NPs, showed that CTAB was not a contributing factor for the increased antimicrobial efficacy of the gold-spherical 70C NPs. However, ROS levels quantified for those bacteria treated with non-spherical gold NPs compared to untreated microbes did reveal rapid ROS augmentation. Furthermore, this rapid ROS burst was correlated with increased membrane permeability as evidenced using the hydrophobic *N*-phenyl-1-napthylamine (NPN) and cell-impermeable PI dyes. Increases in NPN fluorescence intensity indicated the entrance of the dye into the periplasmic space between the outer and inner membrane of Gram-negative bacteria, a process only possible in the presence of a disturbed microbial outer membrane. These bacterial membrane disturbances were observed for all gold NP shapes except the two spherical gold NP controls. AFM studies further supported the presence of compromised membranes after exposure to these four gold NP shapes, revealing changes in morphology to both *E. coli* and *P. aeruginosa* for the non-spherical controls. These results led to the authors concluding that ROS production leading to disruption of membranes was the likely mechanism of action. A second hypothesis proposed by the authors, suggested the increased efficiency over other ROS inducing gold NPs might be due to the increased rugged shape of the NPs, paired with the higher aspect ratios, rupturing membranes in a similar method to that reported for nanopatterned materials.^347^ A report by Nasser *et al.* found a similar phenomenon with positively charged gold nanorods promoting ROS formation.^348^ Highlighting avenues for clinical applications, the gold NPs tested in this study were found to remain effective in the presence of urine, offering potential use as a urinary catheter coating to eliminate urinary tract infections.

#### 4.1.3. Current research in organic NPs

**Fig. 21:**
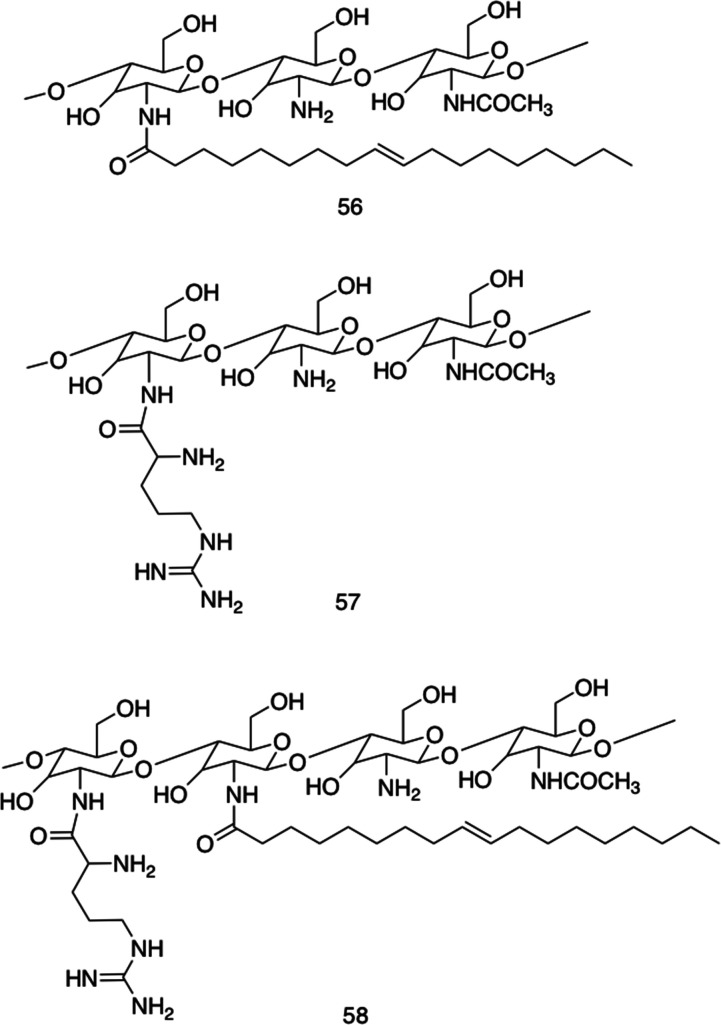
The three synthetically altered chitosan polymers 56–58 synthesised by Ahmad *et al.*^349^

Organic NPs encompassing a diverse range of structures have been developed from materials including natural or synthetic polymers, lipids and proteins. One example of this is the polymer chitosan, which offers benefits associated with biodegradability, biocompatibility and multiple functional groups to enable chemical modification.^275^ Ahmad *et al.* developed the three synthetically altered chitosan polymeric materials shown in Fig. 21, capturing the amphiphilic and membranolytic propensities commonly seen with antimicrobial peptides within a synthetically altered chitosan scaffold. Membranolytic propensities refer to a compounds ability to disrupt the membrane of its target; these were discussed earlier in the self-assembling peptide section. The synthetically altered chitosan polymers assembled into NPs in aqueous solution with diameters ranging from 202–287 nm.^349^ Of the three synthetically altered chitosan polymeric NPs produced (Fig. 21), compound 56 contained lipophilic oleic acid conjugated onto the monomeric units, 57 contained arginine conjugated onto the monomeric units and 58 contained a mixture of the two different conjugated monomeric units (Fig. 21). Those chitosan polymers containing lipophilic oleic acid chains (56 and 58) formed smaller NPs, with increased uniformity, compared with the 57 polymer. Zeta potential measurements identified compound 58 NPs as the most stable structures (31.3 ± 0.5 mV), with compound 56 NPs and 57 NPs both presenting values of ≈20 mV. The resulting NPs formed from these materials were tested against a range of Gram-positive: *B. subtilis* (ATCC 6631), *Bacillus cereus* (*B. cereus*) (ATCC 6633), *S. aureus* (ATCC 25923), methicillin sensitive *S. aureus* (MSSA) (ATCC 43300), fusidic acid-resistance *S. aureus*, coagulase negative *staphylococcus* (CONS), and Gram-negative bacteria: *E. coli* (ATCC 25922), *A. baumnaii*, *P. aeruginosa* (ATCC 27853), and compared against the commonly used antibiotics, vancomycin and gentamicin as controls. All three chitosan NPs displayed broad spectrum antimicrobial activity, with MIC values between 0.125–0.5 mg mL^−1^. Importantly, compound 56 and 58 NPs gave MIC values comparable to that of vancomycin against MRSA, while compound 57 NPs presented an MIC two-fold less potent than 56 NPs and 58 NPs. Therefore, the addition of the lipophilic chain correlates with an increase in antimicrobial efficacy against all bacteria tested. Overall, compound 58 NPs displayed the greatest antimicrobial activity against the Gram-negative bacteria tested (*E. coli*, *A. baumnanii* and *P. aeruginosa*). In order to gain insight into the mode of antimicrobial action, TEM was utilised to investigate the morphology of *P. aeruginosa* after treatment with compound 58 NP. Images of treated cells revealed ruptured structures, indicating leakage of the cytoplasm. The degree of cytoplasmic leakage was quantified through the monitoring of increased UV-Vis absorbance at 260 nm, corresponding to the concentration of nucleic acid free in solution. Presented with these data, the authors proposed membrane targeting as the primary mechanism of action.^350^ To investigate cytotoxicity, human RBCs were selected for testing, with the 100% lysis value determined using Triton X-100.^351^ All three polymer NPs reported less than 5% lysis of RBCs at polymer concentrations of 1 mg mL^−1^. Further cytotoxic investigation was then conducted using HEK293 (embryonic human kidney cells) and HepG2 (human liver hepatocellular carcinoma cells) utilising an MTT assay. Compound 58 NPs, the best performing antimicrobial, displayed no cytotoxicity up to a concentration of 0.5 mg mL^−1^ for both cell lines, while at 1 mg mL^−1^ 40% of cells remained viable, however this value is approximately 10-fold of its MIC.

**Fig. 22:**
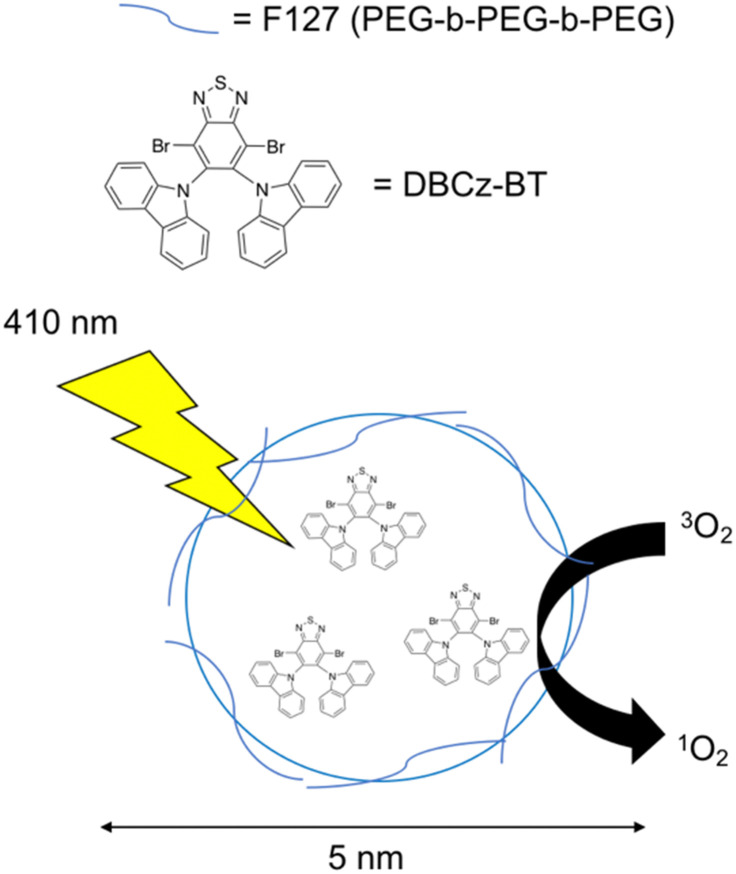
A cartoon illustrating the triblock copolymer F127 encapsulating (4,7-dibromo-5,6-di(9*H*-carbazol-9-yl)benzo[*c*][1,2,5] thiadiazole (DBCz-BT), forming nanoparticles. When irradiated at 410 nm, the encapsulated DBCz-BT can generate active singlet oxygen species.^353^

One disadvantage associated with organic NPs is the reduced ability to achieve desired optical, electronic and magnetic properties commonly associated with inorganic NPs.^352^ Wang *et al.* focussed on overcoming this issue by successfully synthesising water-soluble metal-free phosphorescent NPs capable of generating active singlet oxygen upon photoexcitation, as illustrated in Fig. 22.^353^ The phosphorescent NPs were synthesised using (4,7-dibromo-5,6-di(9*H*-carbazol-9-yl)benzo[*c*][1,2,5] thiadiazole (DBCz-BT) powder encapsulated with an amphiphilic triblock co-polymer (F127) capable of aggregating in aqueous solution (Fig. 22). TEM studies showed the NPs produced to be less than 5 nm, with investigations into the excitation spectrum of the phosphorescent NPs revealing extension of excitation up to 520 nm, enabling visible light excitation. Singlet oxygen generation, the proposed mechanism by which these NPs would achieve antimicrobial activity, was measured by tracking the formation of this species using the chemical scavenger 2,2′-(anthracene-9,10-diylbis(methylene)) dimalonic acid (ADMA), the results of which reported the rapid generation of singlet oxygen in the presence of the phosphorescent NP upon radiation.^354^ The antimicrobial efficacy of this NP was first measured *in vitro* against *E. coli* (ATCC 25922) and MRSA (ATCC BAA40) by incubating the bacteria with phosphorescent NPs for one hour, followed by irradiation at 410 nm, and a subsequent two hour incubation of the bacterial culture in the absence of light. Here a NP loading of 0.8 mg mL^−1^ reported almost 100% bactericidal action after 10 minutes irradiation for *E. coli* and 5 minutes irradiation for MRSA. NP cytotoxicity was then investigated against a mouse C2C12 myoblast cell line *via* a live/dead cell viability assay. The cells were inoculated on a tissue culture polystyrene (TCPS) plate and allowed to adhere before application of phosphorescent NPs. Compared to the TCPS control, cell viability was over 95% even after 5 days of incubation with the phosphorescent NPs at 0.8 mg mL^−1^, showing these NPs to be biocompatible under these experimental conditions. Further to this, the therapeutic effects of the phosphorescent NPs against MRSA, using the rat MRSA skin burn infection model were investigated. This model was chosen due to the high incidence of death resulting from sepsis in burn victims, often triggered by infection.^355,356^ A burn wound was inflicted on the back skin of mice, followed by inoculation with MRSA, a common coloniser of burn wounds. The treatment group received phosphorescent NPs, while the control group did not, with both groups receiving irradiation with visible light. Treatment with the phosphorescent NPs achieved a 1 log reduction in bacterial CFU after one day compared to the untreated control, increasing to a 2 log reduction after three days. SEM imaging also revealed a visual reduction in the concentration of bacterial cells. Finally histopathological samples of the untreated group revealed breakage and inflammation of the epidermis, while the phosphorescent NP treated group showed epidermis integrity comparable to that of healthy skin. Together, phosphorescent NPs displayed effective *in vivo* treatment of MRSA, while displaying no skin irritation or cytotoxicity.

#### 4.1.4. Benefits and drawbacks of NPs

The inherent property of high aspect ratio present with all NPs (independent of inorganic or organic classification) is an important property to consider when comparing antimicrobial materials such as those discussed within this review. This property is both beneficial to achieving high antimicrobial efficacies through increased surface for reactions with microbes,^357^ whilst also introducing the drawbacks of accumulation and delayed clearance from cells.^358,359^ In addition, relatively little is known about the dosing of NPs due to the few clinical trials conducted in this area, an important topic that must be addressed before widespread clinical translation is achieved.^359,360^ Together, this trade-off highlights the importance of the material shape to the resulting mechanisms of action and off target effects. In addition to the overall shape and size of the material, the properties of the building blocks that constitute the final material play an important role in the antimicrobial efficacy and cytotoxicity of the NPs. Below the key benefits and drawbacks incurred as a result of inorganic or organic building blocks making up the NPs are compared.

##### 4.1.4.1. Inorganic NPs

Inorganic NPs have several advantages over their organic counterparts including facile preparation and readily accessible surface conjugation chemistry.^361^ Inorganic NPs also offer a range of desirable and tailorable optical, electronic and magnetic properties due to the presence of various metals within their structure.^102^ For example, gold NPs demonstrate intrinsic light scattering and photothermal properties.^362,363^ These characteristics can be easily controlled through modification of NP size, shape, structure and composition.^363^ In addition, this class of NPs are often chemically inert, stabile under biological conditions and have potential for chemical functionalization.^292^

However, inorganic NPs also have several inherent drawbacks for development for biomedical applications. Inorganic NPs (for example NPs containing silver and copper) have been shown to exhibit cytotoxic,^364^ genotoxic^365^ and carcinogenic^366^ properties.^327^ The cytotoxicity observed has been attributed to a variety of effects, including off-target ROS generation, cytoskeletal defects (as observed with titanium dioxide NPs inhibiting tubulin polymerisation),^367^ alteration of intracellular signalling pathways and, intracellular NP degradation.^367,368^ In addition, several studies have witnessed ROS burst oxidative modification of biomacromolecules including proteins and nucleic acids.^369,370^ This is where a large concentration of ROS such as superoxide and hydrogen peroxide are produced and react with biomolecules, disrupting their proper functioning.^371^ NP induced genotoxicity results as a consequence of detrimental alterations to genetic material and machinery. Carcinogenicity is another drawback associated with inorganic NPs that has been observed in several *in vivo* studies investigating heavy metal NPs, including cobalt and nickel-based NPs.^372,373^ However, it is important to note that heavy metal NPs (silver, manganese, nickel, iron and cerium) are more commonly associated with all of the above toxicities, whilst lighter metal NPs (magnesium, sodium) generally display comparatively low toxicity.^374^

##### 4.1.4.2. Organic NPs

In comparison to inorganic NPs, organic NPs generally present high levels of biocompatibility.^375^ Furthermore, organic NPs also display a wide range of surface and core chemistry due to the larger range and size of compounds that comprise the NP, which offer more functional groups available for modification than their metal counterparts, exemplified by the discussed chitosan NP developed by Ahmed *et al.*^349^ High levels of biodegradability and effective endocytosis at the biological target, and high payload loading efficiency, exemplified by the amphiphilic co-polymer poly(lactic-*co*-glycolic-acid) are also observed.^376^ Furthermore, high levels of structural diversity are achievable with organic NPs, which include the production of micelles, liposomes, nanogels and dendrimers.^377^

One of the primary drawbacks observed with organic NPs is the reduction in the additional properties that can be incorporated through the inclusion of metals.^102^ Optical, electronic and magnetic properties, are less easily instilled into purely organic NPs due to the absence of localised surface plasmonic resonance incurred at the interface of metallic structures.^378^ However, researchers have managed to incorporate unique functionalities such as NPs capable of oxygen generation *via* photoexcitation, through the encapsulation of a metal-free organic phosphor as discussed previously.^353^ Furthermore, the stability of organic NPs is often reduced compared to inorganic NPs. Many inorganic NPs, such as gold NPs, are widely accepted as robust and inert, with little degradation observed,^379^ whilst organic NPs require greater considerations in their preparation and storage conditions to maintain long term stability, as demonstrated in a study of various polymer NPs by Lemoine *et al.*^380,381^

### 4.2. Nanopatterned materials (NPMs)

The potential to increase a materials antimicrobial activity through optimization of its surface properties cannot be underestimated. Specifically, when considering the aforementioned antimicrobial agents, including self-assembling peptides, small molecules and macromolecules, initial binding events rely on antimicrobial surface interactions with microbial membranes.^141^ Furthermore, the mechanism of action for a multitude of antimicrobial agents is through membrane disruption *via* bacterial surface binding events, highlighting the importance of bactericidal membrane interactions.^220,258^ The field of nanopatterning looks to pattern materials on a nanometre scale, in order to enhance performance and instil new functionalities such as antimicrobial activity.^382^ Such surface modifications are commonly introduced through photolithography,^383,384^ electron beam lithography,^385^ reactive-ion etching and nanoimprint lithography.^383–386^ However, these techniques are technically demanding and costly.^382^ Nanopatterning utilising molecular self-assembly is an emerging field of interest, mitigating the complex processes of lithography, although the development of this mechanism is still in its infancy in the field of antimicrobials.

**Fig. 23:**
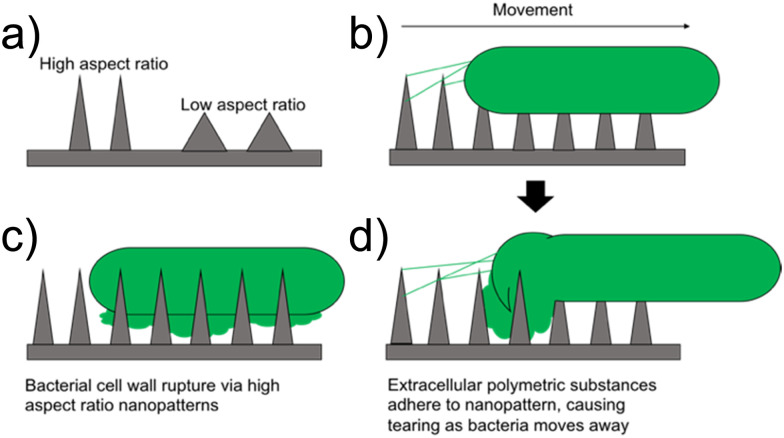
Aspect ratio is an essential feature of antibacterial nanopatterned materials. (a) The difference between high and low aspect ratio nanopatterns. (b) One proposed mechanism of antibacterial action where the 3D patterns rupture the cell wall. (c and d) A second proposed mechanism whereby the bacteria firstly adhere to the nanopattern (c) which results in the tearing of the bacterial cell wall (d).

An ever increasing number of studies have identified NPMs that kill bacteria without the use of antibiotics, presenting a promising avenue for the development of antimicrobial implantable medical devices.^387,388^ The development of antimicrobial materials that can still facilitate human cell adhesion and proliferation are also of great interest.^386^ NPM shapes that have been utilised in this field include nanopillars, nanogrooves, nanopits, nanowires and nanospikes, all of which can be depth, height, width and overall aspect ratio modified.^389–391^ In particular, aspect ratio has been identified as an important factor which influences the antimicrobial action of NPMs.^388^ The difference between high and low aspect ratio nanopatterns are shown in Fig. 23a. Mechanical deformation is the primary mechanism by which bactericidal antimicrobial activity is achieved, whether that be *via* cell wall rupture from high aspect ratio nanopatterns, or extracellular polymeric substances attachment followed by tearing upon attempted cell movement away from the surface, or a combination of the two, remains a topic of debate (Fig. 23b–d).^388^ Either way, the unique mechanisms arising from the use of physical structures represents an innovative method for overcoming AMR. A comprehensive systematic review into NPMs from Modaresifar *et al.* identified silicon and titanium oxide as the most widely investigated antimicrobial NPMs at present.^388^ However, black silicon has also been shown to exhibit bactericidal properties and is amenable to various nanopatterning techniques.^392^ Titanium on the other hand presents as a clinically relevant material for nanopatterning due to its large load bearing properties, and is therefore often used in various medical applications.^393^

#### 4.2.1. Self-assembled NPMs

Physical methods of nanopatterning are only applicable to certain surfaces, over a small surface area and are limited in resolution, for example electron beam lithography is generally limited to the production of structures above 30 nm.^394,395^ Recent research has shown that great versatility can be instilled through self-assembling mechanisms, which will be explored in this section of the review.

**Fig. 24:**
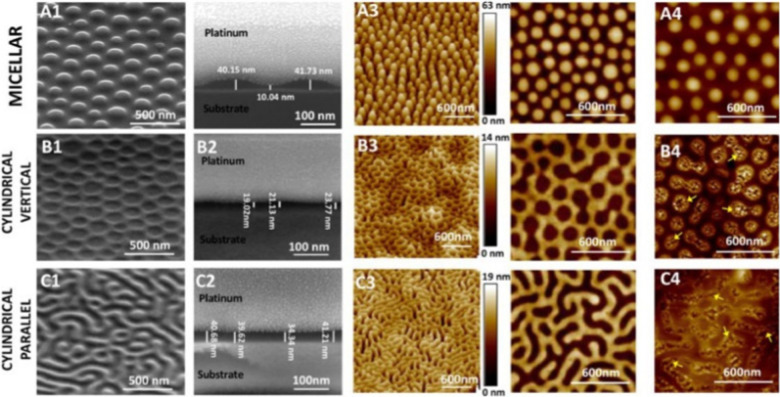
Scanning electron microscopy (1–2) and atomic force microscopy (3–4) images of the (a) micellular (b) cylindrical vertical (c) cylindrical parallel, nanopatterns from Fontelo *et al.*^394^ This image has been reproduced with the permission of Elsevier.

Although self-assembling nanopatterns are currently scarce in the literature, one such example was reported by Fontelo *et al.* who developed bactericidal nanopatterns with three different morphologies *via* block copolymer self-assembly.^394^ Polystyrene-*block*-poly(2-vinylpyridine) (PS-*b*-P2VP) was used to obtain three nanotopographies described as micellular, cylindrical vertical and cylindrical parallel, confirmed *via* both SEM and AFM as shown in Fig. 24. These topographies were achieved by varying processing and annealing conditions. Micelles were synthesised using toluene as the solvent during a solvent vapour annealing process, whilst using chloroform as the solvent yielded cylinders. These small changes show the potential versatility of the self-assembly methodology. The resulting nanopatterns were tested against *E. coli* (ATCC 25922) and *S. aureus* (ATCC 25923), representing Gram-negative and Gram-positive bacterial species respectively. The bacterial species were exposed to the three nanopatterns for 30 minutes and 90 minutes. These times were selected due to their significance as important time points in the race for the surface between bacteria and mammalian cells. This race for survival refers to the theory postulated by Gristina,^396,397^ whereby the presence of a foreign surface, such as an implant, leads to a race between the host cells and bacteria for colonisation of the surface. A live/dead assay was used to determine bactericidal activity, and SEM was utilised to probe the resulting bacterial morphologies. The results for *E. coli* revealed both vertical and parallel cylindrical nanopatterns displayed contact killing properties against *E. coli* after only 30 minutes, with over 70% of adhered cells dead, while the micellular nanopattern displayed bactericidal activity only after 90 minutes, with approximately 50% of adhered cells dead. Images obtained *via* SEM revealed deformed and bent bacterial membranes, suggesting disruption of the cell wall as its mechanism of action. Conversely, none of the three nanopatterns displayed any significant bactericidal action against *S. aureus* after 30 minutes, although parallel cylinders did show 20% bactericidal activity after 90 minutes. SEM of the cells showed no change in the morphology of the membranes, even in the 90-minute parallel cylinder sample, which was effective against *E. coli*. Due to the small height, low roughness and lack of observed piercing of membranes from the tested nanopatterns, the authors suggested that stretching upon adhesion to the nanopattern, followed by rupture, was the likely mechanism of action, a biophysical method recently proposed by Wu *et al.*^398^ This is further supported by indications of *E. coli* undergoing division, seen in captured SEM images, which results in increased fragility of the cell wall, increasing susceptibility to breaking under the proposed stretching force. The lack of rupture observed in *S. aureus* could therefore be due to its thicker and more rigid cell wall, common to Gram-positive bacteria. This is much thicker than the single peptidoglycan layer that constitutes the cell wall of *E. coli* and other Gram-negative bacteria.^399^ Finally, due to the most relevant application of these nanopatterns being in implantable devices, testing mammalian cells adhesion and viability was important. Live/dead assays confirmed that none of the three nanopatterns displayed any cytotoxicity towards mammalian cells at the 30 and 90 minute time points.

#### 4.2.2. Nanopatterns in nature

Alongside self-assembled nanopatterns, there are many interesting examples of NPMs obtained *via* other techniques. Interestingly, nanopatterns displaying antimicrobial properties are rife in nature and have been observed on insect wings, plant surfaces and the skin of sharks.^394^ With breakthrough technological innovation, research has begun both mimicking and improving upon these patterns to take advantage of their antimicrobial properties in the midst of the evolving AMR crisis.

**Fig. 25:**
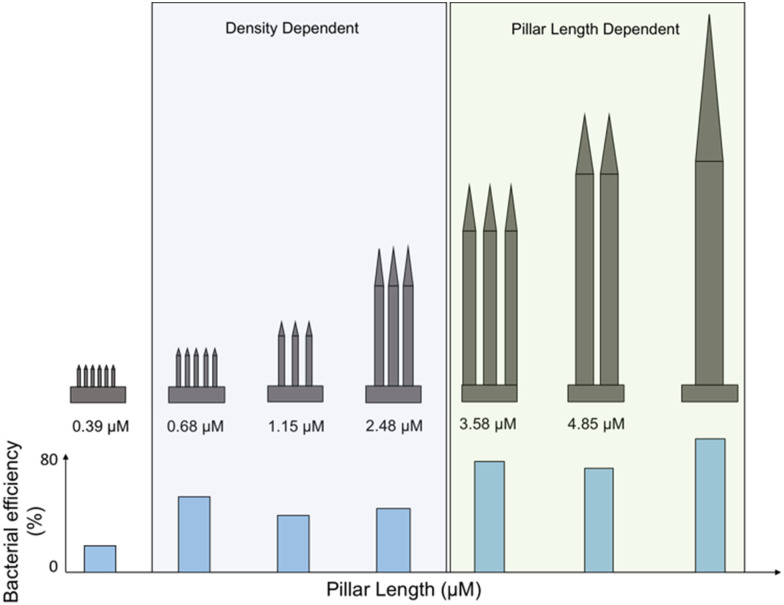
The antibacterial efficacy against *E. coli* DH5α, shown as percentage bacterial efficiency, in response to the different nanopattern pillar lengths, widths and densities from the Michalska *et al.* study.^400^

Inspired by the waxy protrusions observed in nature, on plants and insect wings, Michalska *et al.* developed a black silicon (bSi) based nanopillar covered surface.^400,401^ Nanopillars with a range of nanotopographies were investigated against multiple bacterial species, in order to elucidate the interplay between the nanopatterns dimensions and the spectrum of activity. Broad-spectrum activity is fundamental for application in medical implants.^402^ Without broad spectrum activity, opportunistic pathogenic bacteria may be allowed to flourish, resulting in the surgical removal of the implant due to infection. In this study, the length, tip shape and spacing of the nanomaterial was varied. Reactive ion etching allowed for creation of the bSi nanopillars, with different nanotopographies achieved *via* alteration of the etching time. Increasing the etching times from 1.5 to 30 minutes increased the pillar length up to a maximum of 7 μm and increased the sharpness, while also decreasing the density of the pillars covering the surface. Initial bactericidal screenings were performed *via* deposition of *E. coli* (DH5α) cell suspensions onto the nanopatterned surface, incubating to allow for bacterial growth, and then plating the retrieved cells and comparing the resulting colonies. A smooth, non-etched control surface was also included as a control. A significantly reduced number of colonies was observed on the etched surfaces compared to the control, with the shortest nanopillars (the most similar to naturally found waxy protrusions) showing the lowest antibacterial activity. Of the etched surfaces, for nanopillars between 0.7 μm to 2.5 μm, nanopillar density became the key factor in the bactericidal properties, with bactericidal activity decreasing concomitantly with density, independent of the nanopillar length. However, above 2.5 μm, pillar length became the controlling factor for bactericidal activity, with increased length offering increased bacterial cell death, resulting in the greatest overall activity with the longest/sharpest pillar (length of pillar = 7 μm). The results and pillar formats are summarised in Fig. 25. These initial studies were then repeated with *Rhodobacter capsulatus* (*R. capsulatus*) (U43[pBBR1MCS-2]), *Pseudomonas fluorescens* (*P. fluorescens*) (SBW25), and *B. subtilis* (NCBI 3610). *R. capsulatus* was highly sensitive to the bSi nanopillars, with bactericidal activity proving to be independent of nanotopography, while *P. fluorescens*, and *B. subtilis* presented similar patterns to that of *E. coli* with a lower sensitivity. Gram-positive *B. subtilis*, although containing a thicker peptidoglycan layer than that of the Gram-negative bacteria tested, was more sensitive to the NPM than the Gram-negative *E. coli* and *P. fluorescens*. This suggests the peptidoglycan thickness does not play a key role in protecting the bacteria against the nanopatterned material in this instance. Further investigation into the difference in sensitivity between *R. capsulatus* and *E. coli* revealed *R. capsulatus* killing kinetics were almost an order of magnitude faster than that of *E. coli.* Microscopy images from live/dead staining revealed a similarly high frequency of dead *R. capsulatus* on both the short/blunt and long/sharp nanopillars, confirming fast death rates and lack of variation in death response to the nanopillar morphology. SEM images confirmed the well-established mechanism of cell rupture *via* adhesion of *E. coli* to nanopillars followed by stretching or piercing of the membrane, with blunt pillars presenting no cell rupture. Interestingly, in the case of *R. capsulatus* the shorter blunt pillars appeared to cause stretching and tearing of the membrane *via* multifaceted cellular interactions, such as interaction of bacterial flagella and fimbriae with the NMP. Together these results showed the broad-spectrum activity of nanopillars, in addition to potential selectivity *via* tuning patterning based on the morphology of the bacteria targeted. Furthermore, a greater understanding of the characteristics dictating the antimicrobial effectiveness of the nanopillars was gained, even improving upon the killing efficiency of the nanopattern most closely related to those observed in nature.

Another investigation to gain further insight into the bactericidal mechanisms observed by NMPs inspired by nature was conducted by Jenkins *et al.*^403^ In this study, titanium dioxide nanopillars were created on a titanium surface using a thermal oxidation technique, resulting in a nanotopography mimicking that of the nanoprotrusions on dragon fly wings.^403^ Thermal oxidation is a technique using temperature and oxidation to modify the structure of a nanopattern, often utilising a high temperature (800–1000 °C) during the oxidation stage.^404,405^ Once again the mechanism of contact killing was demonstrated to be multifactorial, although in this instance no resulting mechanical lysis or cell rupture was observed. Deformation of membranes, in addition to penetration of the membrane, was observed utilising TEM to view bacterial cross sections however, the cells present did not show evidence of lysis. Penetration and deformation appeared to be most prevalent when *E. coli* (K12, TOP10) and *K. pneumoniae* (clinical isolate provided by M. Avison) were exposed to the nanopatterned surfaces, both of which display thin peptidoglycan layers. The thicker membrane of *S. aureus* (Newman, SH1000) displayed an observed lower frequency of penetration. Despite no cell lysis being observed, *E. coli*, *K. pneumoniae* and *S. aureus* all presented decreased cell viability. Proteomic analysis seeking to identify differentially expressed proteins (DEPs) in *E. coli* and *S. aureus* was performed, based on the theory that a physiological response to the nanopillars was occurring, resulting in decreased bacterial viability. DEPs are any proteins that show a difference in expression levels over a set threshold between the two samples,^406^ assumed to be differentially expressed as a result of the antimicrobial agent. The resulting DEPs were confirmed to be biologically connected using the STRING application within cytoscape.^407^ This application provides analysis and visual representations of protein networks including those from experimentally determined physical interactions, automatic text mining and prediction based methods.^408^ Within *S. aureus* several markers of oxidative stress were observed, including that of superoxide dismutase, showing a two-fold increase over the non-nanopillar treated sample. *E. coli* displayed a similar change, with the chaperone protein subunit A of the ATP-dependent protease, another commonly inactivated protein from oxidative stress, showing a significant reduction. Thus, it was hypothesised that these nanopatterned surfaces also induce oxidative stress. Although some of the oxidative stress could be attributed to the presence of the titanium dioxide itself,^409^ the authors stated other research has made similar connections between ROS mediated bacterial cell death upon nanostructure contact.^410^ The nanopillar induced bacterial impedance witnessed presents hope for a possible medical implant coating, with several novel observations helping to develop our understanding as to how these nanopatterns achieve their bactericidal activity.

## 5. Nanoscale triggered release of antimicrobial agents

**Fig. 26:**
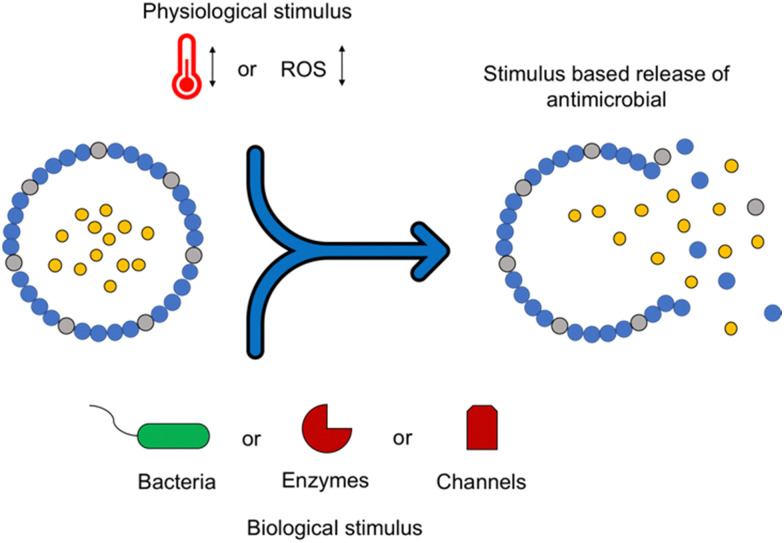
Stimulus based drug release of an antimicrobial agent from a ‘smart material’. ROS = reactive oxygen species.

Over the past decade a great deal of interest has been directed towards the development of smart materials, including nanomaterials and nanocarriers, capable of activating in response to a specific stimulus to release a payload,^411^ as exemplified by numerous recent reviews.^412–415^ With the improvements in smart material technology, utilisation of these strategies as controlled release mechanisms for the delivery of antibiotics has become another avenue of investigation in the AMR crisis (Fig. 26). We direct the reader to several detailed reviews in this area with specific focuses on: smart materials that respond to endogenous stimulus;^411,416^ enzyme and pH based release of antimicrobial agents;^417^ wound healing materials;^418^ antimicrobial surface/implant coatings;^419–421^ antimicrobial hydrogels;^422,423^ metallic nanoparticle delivery systems and;^424,425^ graphene oxide-based smart materials.^426^ Herein, we summarize key examples within the field of triggered release materials that exemplify stimuli responsive nanoscale drug delivery systems that increase the efficiency of antibiotics, stimulate the immune system, overcome resistance, are activated selectively by specific pathogenic bacteria or act as antimicrobial coatings.

**Fig. 27:**
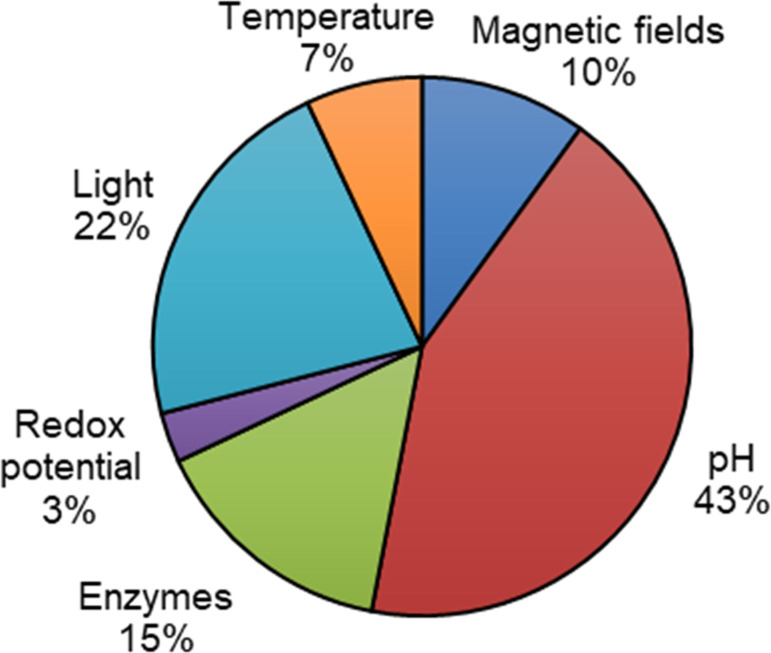
Articles published on stimuli responsive drug delivery for antibacterial treatment in last 10 years.^416^

Stimuli that have been investigated for triggering release of antimicrobial drugs include changes in pH, the presence of specific enzymes, redox potential, and the presence of bacterial exotoxins all of which are summarized in Table 2. Examples of the design strategies that have achieved stimulus-based drug delivery are covered in Table 3. Furthermore, Fig. 27 categorises by stimulus used, the relative percentage of triggered release drug delivery systems developed over the past ten years.^416^

**Table 2:** Summary of the changes in physiological factors that occur in response to bacterial infection

Physiological factor	Change that occurs
Temperature	Changes in local temperature^427^
pH	Decrease in pH of tissue microenvironment^428^
Redox	Increase in GSH levels^411^
	Increase in ROS production due to immune response to bacteria^429^
Enzyme	Production of enzyme by bacteria *e.g.* β lactamse^430^ thrombin like enzyme^431^
Bacterial exotoxin	Alpha toxin, C3bot, streptolysin O, Shiga toxin^432^

**Table 3:** Design strategy for various infection associated stimuli

Stimulus	Design strategy	Material	Active agent
pH	Protonation or charge shifting	Poly(d,l-lactic-*co*-glycolic acid)-*b*-poly(l-histidine)-*b*-poly(ethylene glycol)^433^	Vancomycin
pH	Acid liable linkage	SA-3M solid lipid nanoparticle^434^	Vancomycin
Enzyme	Enzyme cleavable linker in structure	Copolymer of poly(ethylene glycol) and poly(ε-caprolactone)^435^	Vancomycin and ciprofloxacin
Enzyme	Enzyme cleavable linker in structure	PEG-*b*-PP and PEG-*b*-PC^430^	Vancomycin and gentamicin
Redox	Incorporation of reduction\oxidation sensitive bond	Mesoporous silica nanoparticle^436^	Chlorhexidine and silver ions
Redox	Incorporation of reduction\oxidation sensitive bond	RBC membrane coated nanogels (polyacrylamide and cysteine dimethylacrylate^437^	Vancomycin
Cold atmospheric plasma	Oxidative cleavage	Polyacrylamide hydrogel^10^	Alizarin red S

### 5.1. Triggered release of antimicrobial agents in research classified by stimulus

#### 5.1.1. Enzyme and exotoxin triggered systems

Various pathogenic bacteria produce exotoxins and enzymes that can be exploited as a stimulus to enable bacteria specific drug delivery.^438,439^ These exotoxins, released by pathogens, act on host eukaryotic cells causing post-translational modifications of host proteins, often resulting in the manipulation of cellular signalling cascades and inflammatory responses.^432,440^ These enzymes expressed by bacteria can be involved in multiple functions including the development of AMR.^438^ The use of materials sensitive to cleavage or hydrolysis by enzymes, such as those summarized in Table 3, have been shown to effectively act as nanocarriers for drug delivery, releasing their cargo *via* reactions catalysed in the presence of these enzymes.

**Fig. 28:**
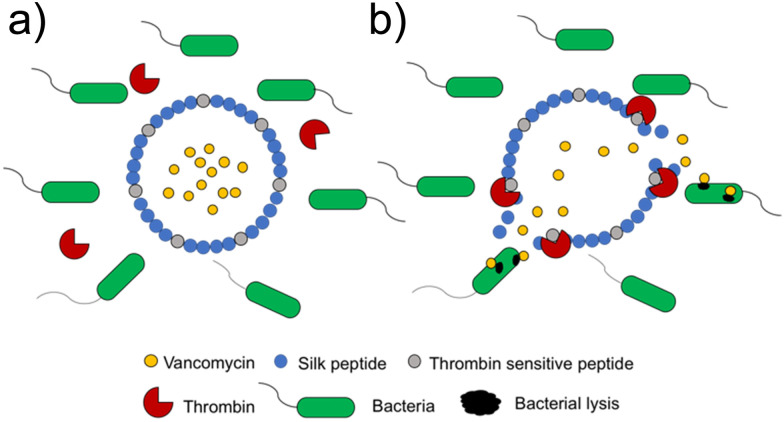
(a) Thrombin sensitive peptide conjugated silk peptide nanosphere. (b) Activation of nanosphere due to thrombin.^441^

Mulinti *et al.*, used a thrombin sensitive peptide (TSP) to link spider silk proteins, creating a delivery vehicle with a hydrophobic cavity capable of encapsulating the antimicrobial vancomycin (Fig. 28).^441^ Successful production of TSP-spider silk nanospheres was confirmed *via* DSC, and the TSP linker was shown to be selectively cleaved in the presence of *S. aureus* (ATCC 49230), with no cleavage witnessed in the presence of water or human thrombin. In *S. aureus* infections, through the release of staphylocoagulase and von Willebrand factor-binding protein, the production of staphylothrombin complexes is possible upon cleavage of the human protein prothrombin.^431,442^ This thrombin (an enzyme present in blood plasma^443^) is produced through the bacterial enzyme cleavage of prothrombin and has been found to display different downstream properties to thrombin produced through human enzymatic cleavage, including the inability to directly activate human platelets. The TSP-spider silk nanospheres created by Mulinti *et al.* capitalised on these differences, gaining selective cleavage from the staphylothrombin over human thrombin. The CAC of the resulting nanospheres was determined to be 53.7 μM, with the average diameter of 184 nm ± 12 nm and a zeta potential of −16 mV suggesting the presence of stable nanospheres. Antimicrobial activity was first investigated against *S. aureus* (ATCC 49230), with an MIC of 16 μg mL^−1^ obtained. Vancomycin alone achieved an MIC of 2 μg mL^−1^, while the shell material alone exhibited no detectible antimicrobial activity. *In vitro* studies were then performed in the presence and absence of *S. aureus* to determine the release profile of vancomycin. In the presence of *S. aureus* 84.4% of vancomycin was released, while for the same nanocarriers in the absence of *S. aureus* only 18.9% of the vancomycin was released. Furthermore, when tested against *S. epidermidis*, 20.8% vancomycin was released, only slightly higher than the control, suggesting selective release against *S. aureus.* Moving forward, *in vivo* studies were performed on a septic arthritic rat model. Here, *S. aureus* at a concentration of 10^2^ CFU mL^−1^ was inoculated into the knee joint, with treatment starting two days post inoculation. To elucidate the treatments effectiveness, cultures from the treated and untreated groups were taken and the CFU mL^−1^ was determined. Bacteria culture taken from the group treated with the nanocarriers displayed an average of 40 CFU mL^−1^, while cultures of the non-treated groups presented an average of 810 CFU mL^−1^, confirming the *in vivo* infection responsive release of vancomycin.

**Fig. 29:**
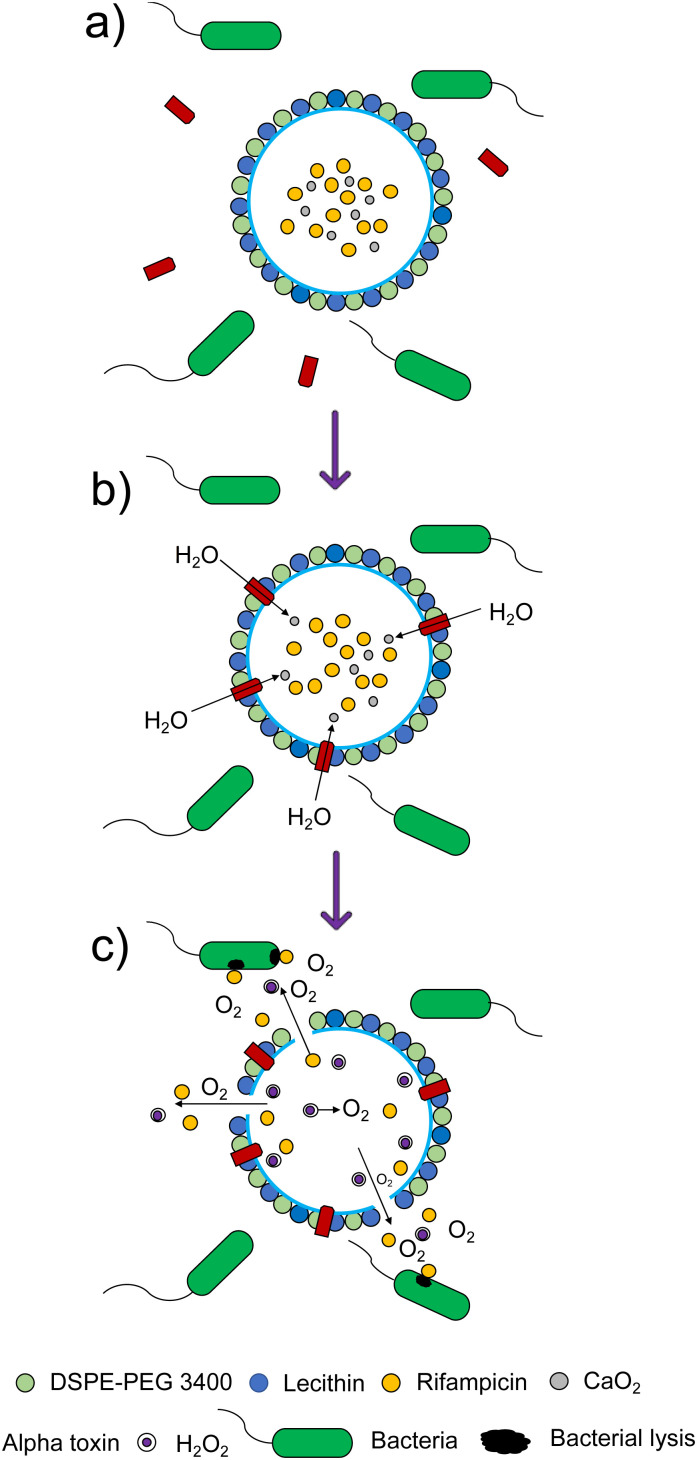
Activation and action of liposome based Nanoreactor.^43^ (a) DSPE-PEG3400, lecithin, lauric acid and stearic acid liposome containing calcium peroxide and rifampicin surrounded by bacteria and alpha toxin; (b) alpha toxin integrates into the membrane, forming a pore through which water enters; (c) water reacts with calcium peroxide to form hydrogen peroxide which decomposes to oxygen gas. The evolution of this gas promotes the release of rifampicin from the liposome.

An exotoxin produced by *S. aureus*, known as alpha toxin (a pore forming toxin)^444^ was targeted through liposome based nanoreactors synthesized by Wu *et al.*,^43^ which were shown to neutralize the toxin whilst also triggering rifampicin delivery. Liposomes were synthesized using lauric acid and stearic acid, which in turn were coated with Lecithin and 1,2-distearoyl-*sn-glycero*-3-phosphoethanolamine polyethylene glycol 3400 (DSPE-PEG3400). The antibiotic rifampicin, in addition to calcium peroxide were then encapsulated within the liposome, producing the nanoreactor. This design allows stimulus triggered delivery due to encounters with alpha toxin, which integrates into the nanoreactor and forms a pore in its membrane (Fig. 29). These pores cause an influx of water, which react with the encapsulated calcium peroxide to form hydrogen peroxide. Hydrogen peroxide decomposes into oxygen which in turn leads to rifampicin release from the nanoreactor.^445^ This mechanism is summarised in Fig. 29. SEM and TEM were used to confirm the presence of nanoreactors, displaying spherical structures with uniform sizes in the range of 150–200 nm. The capture of alpha toxin was investigated utilizing immunogold tagged alpha toxin and TEM visualization, confirming efficient capture of alpha toxin by the nanoreactor without compromising structural integrity. Specifically, 100 μg of nanoreactor was found to capture 4 μg of toxin. Pore formation as a result of toxin integration was confirmed with SEM, while fluorescence microscopy with 8-aminonaphthalene-1,3,6-trisulfonic acid disodium salt (ANTS) and *p*-xylene-bis-pyridinium bromide (DPX) as a pair of fluorophore/quencher^446,447^ molecules loaded into the nanoreactors confirmed, through increased fluorescence, the successful release of rifampicin in the presence of MRSA (clinical isolate). When repeated in the presence of *B. subtilis* (AB 90008) (which does not produce alpha toxin) and PBS, no increase in fluorescence was witnessed, suggesting no rifampicin was released in these control conditions, due to insignificant volumes of dye able to exit the nanoreactor. Further affirmation was gained through a hydrogen peroxide assay, whereby hydrogen peroxide concentrations were measured in the presence and absence of alpha toxin. In the presence of alpha toxin, hydrogen peroxide was shown to increase overtime, reaching a maximum of 2.09 mmol L^−1^ at 120 minutes (79.15% of theoretical production), followed by a large reduction when tested beyond 120 minutes due to subsequent hydrogen peroxide decomposition. When treated with water, the maximum hydrogen peroxide concentration reached was 0.32 mmol L^−1^, therefore showing alpha toxin allowed water influx to react with the encapsulated calcium peroxide, producing hydrogen peroxide. Antimicrobial studies were first performed *in vitro* against MRSA and *B. subtilis*, revealing potent activity against MRSA when treated at 100 μg mL^−1^. Specifically, 98.19% of MRSA was inhibited by the nanoreactors, while only 22.64% of *B. subtilis* was inhibited at the same concentration, rising to 96.71% when alpha toxin was added to the nanoreactor in the presence of *B. subtilis*. Cytotoxicity was probed with an MTT assay, with >90% cell viability of Vero cells (monkey kidney epithelial cells)^448^ observed after 24 hours of nanoreactor treatment up to 500 μg mL^−1^. Histological analysis verified these results, revealing no significant difference in tissue damage, inflammation or lesions between control mice and nanoreactor treated mice. Additionally, treatment with these nanoreactors caused a decrease in haemolytic rate compared to the positive control group, due to the capture and neutralization of alpha toxin. Finally, *in vivo* antibacterial activity against MRSA was studied using a mouse skin infection model. The importance of this model was expressed by authors, highlighting that MRSA represents one of the most common causes of skin infections in hospitals.^449^ Each mouse was inoculated with 100 μL of 10^6^ CFU mL^−1^ of MRSA, followed by treatment with 20 μL of 1 mg mL^−1^ of nanoreactor or control applied onto the wound 24 hours post inoculation. The treatment was performed for three days in total post inoculation. The wound tissue and organs were excised at 4, 6, 8 and 10 days post treatment with day 0 excised as a control. The group treated with nanoreactors displayed a significantly higher wound healing rate than the control groups (PBS, shell alone, shell with calcium peroxide only and shell with rifampicin only), in addition to presenting higher inhibition efficiency of the MRSA than the control groups and reduced numbers of inflammatory cells. Furthermore, the control groups all presented unrepaired collagen, whilst the nanoreactor treated group presented well established collagen. Additionally, it was shown the nanoreactors increase toxin neutralization, significantly reduced haemolysis and boosted immune response to alpha toxin.

**Fig. 30:**
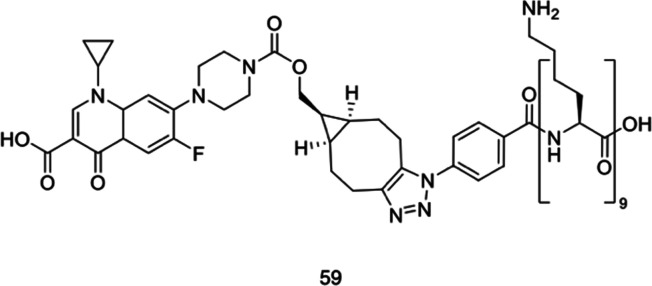
Structure of compound 59.^453^

Infections associated with biomedical implants are a major cause of failure for prosthetic devices.^450^ These infections can develop months after the surgery, and can be initially asymptomatic rendering them hard to detect.^451^ Microbial contamination on implants often leads to removal of the implant due to infection.^452^ One strategy used to combat these implant infections is to nano-coat the implant with a material that prevents bacterial adhesion and/or releases an antimicrobial agent in response to either the presence of the bacteria or the secondary immune responses triggered. One such coating material was developed by Bouragat *et al.* utilizing enzymes for triggered antibiotic release.^453^ Here, by using a poly-l-lysine (PLL) conjugated with ciprofloxacin, NPs were synthesized through ionotropic gelation with alginate. PLL was conjugated with ciprofloxacin *via* copper free azide-alkyne 1,3-dipolar cycloaddition with the PLL chain producing 59 (Fig. 30). Mixing of 59 and alginate in aqueous solutions at different ratios led to NP formation through ionic interactions, ranging from ≈200 to ≈400 nm in size. The final ratio of 1 : 3 59 : alginate was selected to maximise cost effectiveness whilst keeping a low PDI (0.225). NPs proved stable at 37 °C for 50 hours in PBS, and UV-Vis measurements at 278 nm of NPs incubated with both 2 μg mL^−1^ and 5 μg mL^−1^ trypsin showed increased release of ciprofloxacin with respect to time. To investigate the NP use for prosthetic device coating, the NPs were coated on a model polyethyleneimine coated titanium surface by spray coating the surface with a NP dispersion. To demonstrate proof of concept for enzymatic release of ciprofloxacin from this surface, a sample was incubated with the proteolytic enzyme trypsin (5 μg mL^−1^) in PBS solution at pH 7.4, 37 °C. In the absence of trypsin, no change in the nanocoating thickness was observed, while the trypsin treated material resulted in a 45% decrease in the thickness of the nanocoating over a 109 hour time period. This result indicated the successful enzymatic based degradation of the coating. Antimicrobial studies were performed against *S. aureus* (DSM 799) chosen due to its common role in orthopaedic implant-associated infections. Ciprofloxacin conjugated with PLL inhibited 51% of *S. aureus* at a concentration of 12.5 μg mL^−1^. However, the conjugation process was found to reduce the efficacy of ciprofloxacin itself, with the voluminous groups of the linker residue remaining on the ciprofloxacin following cleavage from 59 found to be responsible for this reduction in antimicrobial activity.^453^

#### 5.1.2. pH and ROS triggered systems

**Fig. 31:**
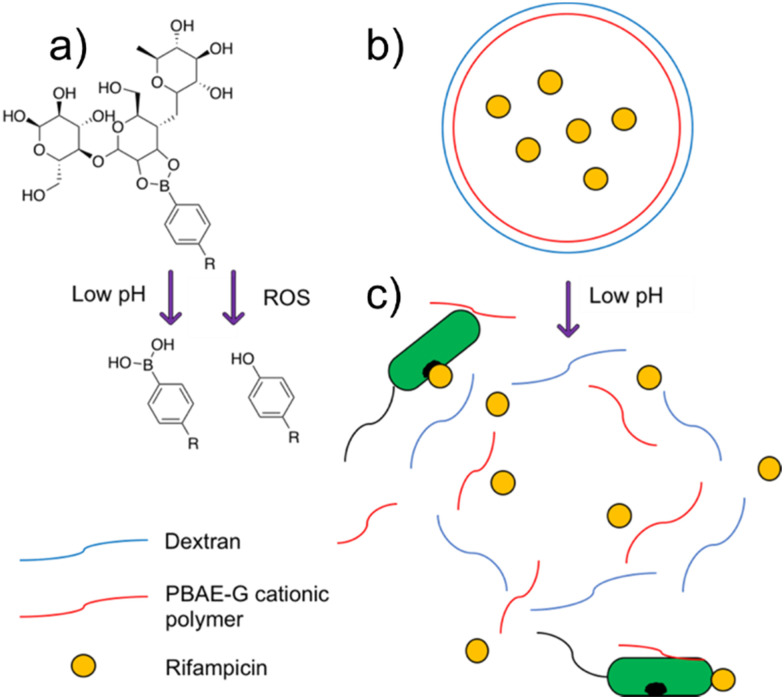
Poly(β-amino ester)-guanidine-phenylboronic acid (PBAE-G-B) cationic polymer based nanoparticle activation.^454^ (a) Dextran is cleaved from PBAE-G-B in a low pH environment. (b) Intact nanoparticle in physiological pH environment. (c) Dextran cleavage at low pH causing release of rifampicin and PBAE-G cationic polymer. ROS = reactive oxygen species.

As a result of inherent immune responses, the microenvironment of infected tissues differs to that of healthy tissue. Infected tissues present a lower pH due to tissue acidosis (reaching a pH of 5.5–7.0),^428,454^ and a higher level of ROS from chemotaxis of pro-inflammatory immune cells.^429^ A multifunctional pH and high ROS responsive NP was constructed by Ye *et al.*, using a dextran shell and a poly(β-amino ester)-guanidine-phenylboronic acid (PBAE-G-B) core to encapsulate the antibiotic rifampicin.^454^ PBAE-G-B is a cationic polymer with a p*K*_a_ value of 6.1; at physiological pH (pH 7.4) PBAE-G-B is hydrophobic, however below pH 6.1 it protonates, becoming hydrophilic. On exposure to low pH (6.0) the PBAE-G-B component dissociates from the dextran shell due to hydrolysis of the phenylboronic ester linkage formed between the boronic acid and the *cis*-diols present in dextran.^455^ A similar result occurs in response to ROS; as the boronic acid is oxidized by the ROS to form the corresponding phenol, it too dissociates from the dextran shell. Both of these effects cause the destabilization and subsequent release of the encapsulated rifampicin (Fig. 31). Initial cytotoxicity studies on the NP indicated high levels of biocompatibility, with an MTT assay against RAW 264.7 murine macrophage cells revealing an IC_50_ of 1297 μg mL^−1^, 6–7 times higher than that of free rifampicin. *In vitro* activity was confirmed against several pathogenic bacteria, with MICs shown in Table 4. Interestingly, the NP proved eight times more effective than rifampicin alone against the rifampicin resistant *Mycobacterium smegmatis* (*M. smegmatis*) (MC^2^155) when administered under oxidizing conditions. To elucidate these findings, a series of experiments involving confocal laser scanning microscopy (CLSM), SEM and an acridine orange/propidium iodide (AO/PI) assay were conducted. In the CLSM experiment, fluorescently labelled rifampicin, bacteria and dextran were used to identify localization to a rifampicin resistant strain of *P. aeruginosa* (ATCC 27853). Results indicated little free rifampicin localized to the membrane, while both free dextran and the NPs synthesized with the labelled dextran showed strong affinity to the bacterial membrane. Furthermore, NP treated bacteria under high ROS or low pH conditions displayed aggregation forming bacterial clusters, a result that was also achieved using labelled PBAE-G-B polymer, suggesting this cationic PBAE-G-B polymer was released and induced bacterial aggregation. Under physiological conditions, SEM presented NP attachment to the bacterial membrane, while under high ROS or low pH conditions membrane disruption and deformation was observed. This membrane deformation was further investigated utilizing the AO/PI staining assay, revealing neither rifampicin nor inactivated NP enhanced bacterial membrane permeability. Conversely, under high ROS or low pH conditions significant fluorescence signal was observed, indicating increased membrane permeability. Therefore, together these results indicated activation of the NP led to cationic polymer release, disrupting the bacterial membrane, allowing small molecules such as PI or rifampicin to enter the cell, hence explaining the increased efficacy of the NP over rifampicin itself against *M. smegmatis.* Development of AMR was investigated against *E. coli*, whereby *E. coli* was incubated with either rifampicin or the NP at a sub-lethal dose for a total of 20 passages. Rifampicin treated *E. coli* displayed a 40-fold increase in MIC by the 20th passage, while the NP only exhibited a two-fold increase in MIC after 16 passages, suggesting low risk of AMR development. Finally, *in vivo* studies were conducted using an MRSA thigh infection mouse model. Here, MRSA was injected into the mouse thigh, followed by intravenous injection of the NP 12 hours post inoculation. NP accumulation was found in the infected thigh one-hour post injection, with cationic polymer release and subsequent agglomeration of the microbes. Excised organs also revealed that the mice thighs showed the highest concentration of the NP of all the organs, besides the liver, (the organ utilized for detoxification), indicating good infection targeting abilities. Additional *in vivo* tests were evaluated against *P. aeruginosa* pneumonia and MRSA induced peritonitis. The importance of the aforementioned models was highlighted by authors, with each of the infection models induced by ESKAPE pathogens, regarded as serious threats by the U.S. CDC. For the pneumonia model, mice were inoculated with 5 × 10^8^ CFU *P. aeruginosa* and subsequently treated with 5 mg kg^−1^ rifampicin or the equivalent dose of NP. The NP treated group displayed a more than four-fold reduction in CFU, while the rifampicin alone group showed very limited efficacy. The peritonitis model involved treatment administration one-hour post infection, with CFU in the organs determined 12 hours post treatment. Treatment with loaded NPs displayed significantly enhanced antimicrobial activity to rifampicin and NP shell alone, with the loaded NPs leading to more than 99.9% elimination of pathogens in all organs tested, whilst a panel of biochemical factors monitored in healthy mice over a three-day course of loaded NP revealed good biocompatibility.

**Table 4:** MIC (μg mL^−1^) value of rifampicin and NP at different conditions of pH and ROS level. Numbers in brackets are fractional inhibitory concentration of rifampicin in μg mL^−1^ ^454^

Bacteria	Rifampicin	NP pH 6.0	NP pH 6.0 + 150 × 10^−6^ M H_2_O_2_
*E. coli* (DH5 alpha)	16	32 (2.7)	16 (1.3)
*E. coli* (ATCC 25922)	16	32 (2.7)	16 (1.3)
*S. aureus* (Newman)	0.016	0.25 (0.021)	0.25 (0.021)
*S. aureus* (MRSA) (ATCC33591)	0.016	0.25 (0.021)	0.25 (0.021)
*P. aeruginosa* (ATCC 27853) (rifampicin resistance)	32	64 (5.4)	32 (2.7)
*M. smegmatis* (MC^2^155) (rifampicin resistance)	128	32 (2.7)	16 (1.3)

The pH of healthy skin presents in the range of 4–6, lower than that of physiological tissue.^456^ In the presence of an infection, skin pH can become elevated; the pH of a normally healing wound ranges between 6.5 to 8.5, whilst that of a chronic wound can reach 7.2 to 8.9.^457^ Such chronic wounds are commonly associated with bacterial biofilms.^457^ Therefore, these pH changes impart the opportunity for pH responsive materials to be utilized in wound healing.^458^ A pH responsive hydrogel dressing was created by Haidari *et al.* using methacrylic acid, acrylamide and *N*,*N*′-methylenebisacrylamide loaded with silver NPs.^459^ The resulting hydrogel showed an increased rate of release of silver NPs at alkaline pH (7.2–10) when compared to acidic pH (4.0). This was attributed to the change in state of the hydrogel from a collapsed state at pH 4, to a hydrated state at alkaline pH. The collapsed state restricted diffusion due to protonation of the carboxyl group of the methacrylic acid, while the hydrated state facilitated expansion and increased diffusion due to deprotonation of the carboxyl group, imparting negative charge and consequently electrostatic repulsion between molecular chains. The antimicrobial activity of the silver NP loaded hydrogels was tested against both the Gram-negative *P. aeruginosa* (PAO1) and Gram-positive *S. epidermidis* (ATCC 35984), chosen due to their prevalence in infected wounds.^460^ Initial zone of inhibition studies were conducted against both bacteria; clear zones were formed around the silver NP loaded hydrogels, with non-loaded hydrogels presenting no zones after 18 hours. These results showed that the hydrogel itself did not contribute to the antimicrobial properties. Subsequent live/dead assays were performed to probe bacterial viability, with subsequent fluorescence microscopy imaging revealing no dead bacterial cells for either *P. aeruginosa* or *S. epidermidis* with the control material, while the silver NP loaded hydrogels showed no viable cells, demonstrating potent bacterial killing against both species of bacteria. Due to the desired downstream application as a wound healing agent, cytotoxicity was probed against human fibroblasts, one of the most important cell types in the wound healing process.^461^ Using resazurin, a compound that can be reduced by live cells to produce intrinsically fluorescent resorufin,^462^ the silver loaded hydrogel was determined to be non-toxic to human fibroblasts, with no difference in the metabolic activity witnessed compared to the blank control.

## 6. Biofilm inhibition through nanoscale materials and self-assembly

**Fig. 32:**
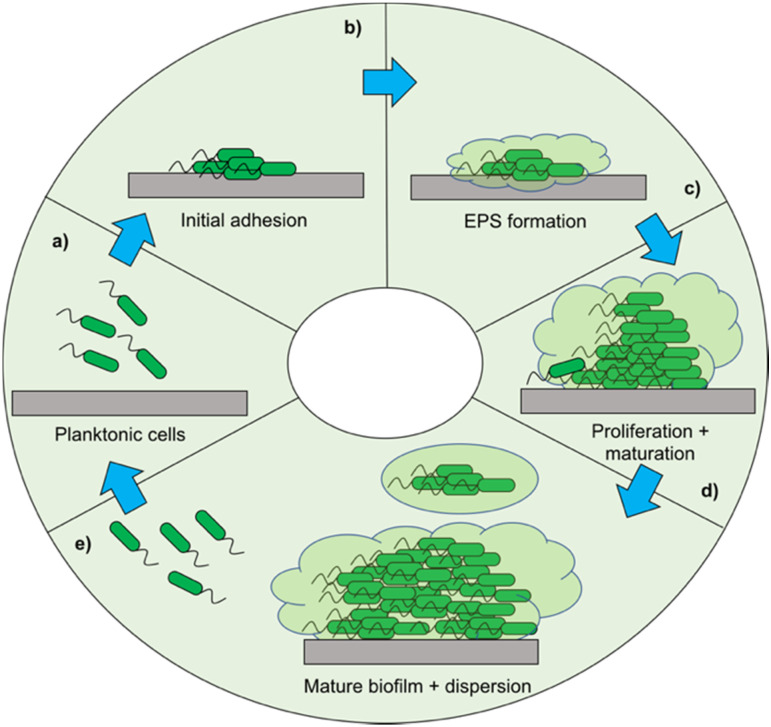
Lifecycle of a biofilm (a) free planktonic cells, (b) bacterial cells reversibly adhere to surface, (c) Irreversible attachment occurs with extracellular polymeric substance (EPS) formation, (d) bacterial cells proliferate and biofilm undergoes maturation, (e) mature biofilm is formed with channels for nutrient transportation and removal of metabolic waste. Planktonic cells are also released from biofilm and can go on to restart the cycle.

**Fig. 33:**
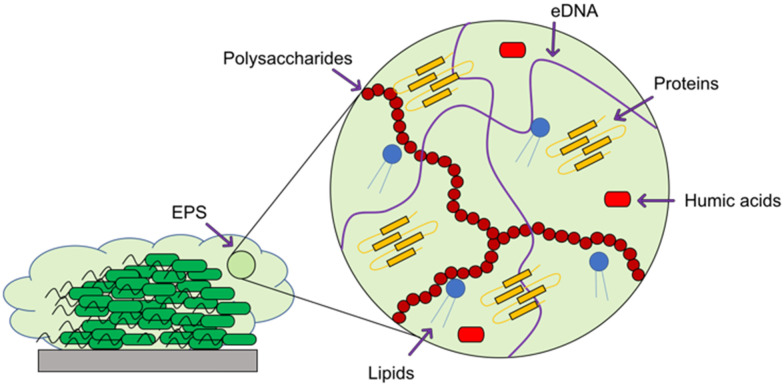
Schematic representation of the composition of biofilm extracellular polymeric substance, including polysaccharides, extracellular DNA, proteins, humic acids and lipids.

Biofilms are defined by Vert as surface-associated aggregations of bacterial cells that are surrounded by an extracellular polymeric substance (EPS).^463^ They can act as pseudo-multicellular organisms,^464^ offering increased fitness and resistance to classic antimicrobial treatments.^464–466^ Methods by which to treat these complex infections have been the subject of a multitude of reviews, to which we direct the reader.^414,467–470^ The formation of the biofilm is dynamic and consists of multiple stages (Fig. 32). Initially, planktonic bacteria reversibly adhere to a surface; individual cells then secrete adhesins, surface proteins that allow the bacteria to specifically attach to a surface,^471,472^ before the EPS causes irreversible attachment, anchoring the aggregation of cells.^473,474^ The EPS is comprised of proteins, polysaccharides, lipids, humic acids and extracellular DNA (eDNA) (Fig. 33).^474,475^

The process by which the bacteria begin to proliferate within the biofilm is termed maturation. Here, the bacteria adhere to each other, increasing the stability of the biofilm, and channels are formed which are used to transport nutrients and remove metabolic waste from the cells contained within.^476,477^ As the bacterial life-cycle continues, planktonic cells may be released from the biofilm to colonise other surfaces.^477,478^ Whole sections of biofilm may also be released, which form secondary biofilms within a much shorter time frame than when seeded by individual planktonic bacteria.^463^ The cell densities of a biofilm can range from 10^8^–10^11^ cells per gram,^479,480^ and can be formed on almost any surface, including but not limited to, dead tissue,^481^ bone,^482^ medical devices/implants (titanium and nickel alloys, polymers and ceramics),^483^ and living tissue (endocarditis).^475,484^

Biofilm formation is triggered by a range of conditions, including, temperature, pH, hydrodynamic forces, gravitational forces, signalling molecules and local conditions,^485,486^ and can cause stage specific expression of genes and proteins.^487,488^ The change in the expression of genes causes phenotypic differentiation of the bacteria as the individual cells develop different attributes, allowing them to carry out different functions.^489–492^ This differentiation is one of the reasons that planktonic bacterial cells are poor models of their biofilm associated counterparts.^493,494^ Comparisons between planktonic bacteria and those within a biofilm are further complicated by the chemical signalling that can occur between individual cells within the biofilm, termed quorum sensing (QS).^495–497^ This intercellular signalling enables the bacteria within the biofilm to act as a community, whether or not they are formed by the same or multiple different species,^498,499^ and communicate information that improves their survival fitness such as nutrient uptake, protein synthesis and EPS production.^500–502^ For example, co-infection of *S. aureus* and *P. aeruginosa* acts as a better *in vitro* model for chronic wounds.^503^ DeLeon *et al.* suggested that these two species have a synergistic relationship as the polymicrobial biofilm has enhanced virulence, persistence and antimicrobial tolerance.^498,503^ The rate of spread of antimicrobial resistance genes is increased within this type of environment *via* horizontal gene transfer (HGT).^475,504^ HGT is the transfer of genetic material between individuals in the population without reproduction (termed vertical transfer).^505^ The matrix formed by the EPS, combined with high cell densities, provides an optimal environment for cell-to-cell contact, a mechanism that is required for some forms of HGT.^475,506^ The EPS also acts as a barrier to the environment,^507^ offering the bacterial community a level of protection from the host's immune system, whilst still allowing for the exchange of nutrients and waste with the external environment *via* pores in the overall structure.^490,508^ The size and nature of these pores however, is such that antimicrobials are not able to easily penetrate biofilms, and are further hindered by adsorption to the EPS matrix and pH gradients within the biofillm.^490,508^

However, unlike their planktonic counterparts, biofilms are commonly less metabolically active, allowing the bacteria within to survive in a dormant state.^509,510^ This dormancy also reduces the efficacy of antimicrobial treatments that target metabolic processes.^511^ The MIC for planktonic bacteria is the lowest concentration of an antimicrobial that will inhibit the growth of a micro-organism.^512^ The measurement of biofilm inhibition, MBEC, differs to that of planktonic bacteria and is defined as the minimum concentration of an antimicrobial required to eradicate a biofilm.^513,514^ In an *E. coli* biofilm the MBEC for five common antibiotics (ampicillin, ciprofloxacin, cefazolin, cefotaxime and trimethoprim-sulfametoxazole) was 1000 times the MIC for the same planktonic bacteria.^473,513,515,516^ Penetrating the EPS is one of the biggest challenges for the treatment of biofilms, as it presents a barrier to diffusion that must be overcome before reaching the bacterial cells.^517,518^ The biofilm is essentially able to act as a reservoir of planktonic bacteria. Only as these cells are released are symptoms expressed, and thus an infection identified, and antibiotics subsequently administered. Due to the inherent resistance of biofilms to these treatments, this often eradicates the planktonic bacteria, but not the biofilm, treating the symptoms but not the cause of the infection,^519,520^ resulting in repeat infection.^521^ To prevent this, either the biofilm itself, and thus the surface it is associated to (*i.e.* a medical device), must be removed from the body, usually through an invasive surgical procedure.^522^

In addition, as previously mentioned, biofilms are able to respond to the environment, including the presence of antimicrobials, and employ protective stress responses.^523^ Biofilms can also detect both direct (one individual harms another) and indirect (resource) competition and respond accordingly.^524^ These responses include, but are not limited to; increasing the production of enzymes that deactivate antibiotics,^525–527^ production of ROS^528^ or increasing the synthesis of matrix polysaccharides to improve the protection offered by the EPS.^527^ Finally, within a biofilm there is usually a small population of tolerant ‘persister’ cells. These cells are inherently resistant to antibiotics and account for 0.001–1% of the population.^529,530^ Persister cells are found deep within the biofilm,^531^ and have the potential to survive antimicrobial treatment, enabling re-infection despite eradication of the rest of the biofilm.^466,532^

### 6.1. Self-assembling molecules and nanoscale materials for biofilm inhibition in research

#### 6.1.1. Thermally responsive nanocoating

**Fig. 34:**
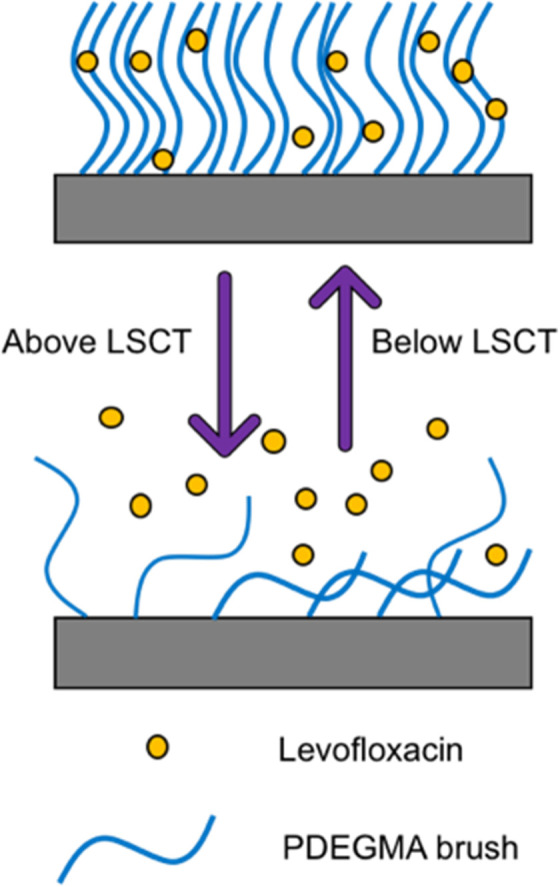
Changes to poly(di(ethylene glycol)methyl ether methacrylate) (PDEGMA) brush on titanium implant above and below the lower critical solution temperature (LCST).^533^ Below the LSCT levofloxacin remains bound to the PDEGMA brushes. Above the LSCT levofloxacin is released from the PDEGMA brushes.

A thermally responsive nanocoating for use on titanium implants was created by Choi *et al.* using poly(di(ethylene glycol)methyl ether methacrylate) (PDEGMA) loaded with the antibiotic levofloxacin.^533^ Significant temperature increases are observed at the site of an infection,^427^ allowing these changes to be utilised as a stimulus for triggered release. These PDEGMA brushes were shown to have a dry thickness up to 400 nm. The antibiotic was confirmed to be released when the local temperature of the implant increased above the lower critical solution temperature (LCST) of the PDEGMA (32 °C) (Fig. 34). When incubated at 37 °C, the levofloxacin release showed initial burst in concentration in the first 5 minutes, followed by progressive release over a period of 4 hours, with an increase in the rate of drug release observed with increasing temperature above the LCST. MIC and MBC values required for levofloxacin against *S. aureus* (ATCC 13709) were achieved within a 1 mm distance of the PDEGMA brush surface at 37 °C, with the released concentration of levofloxacin at the first time point collected high enough to inhibit the growth of *S. aureus*. The *in vitro* antifouling of the PDEGMA coated titanium plate was tested against *S. aureus* (ATCC 29213) with a bare titanium plate used as a control. After 72 hours SEM revealed the bare titanium plate showed biofilm development while the PDEGMA titanium plates were biofilm free. Furthermore, after 24 hours of incubation with a highly concentrated *S. aureus* solution (10^9^ CFU mL^−1^) the PDEGMA brush coated titanium surface showed a 90% reduction in living bacteria. *In vivo* tests were conducted using the back of rats as the infection model, infected with *S. aureus* (Xen29). As with the *in vitro* studies, bare titanium implants were used as the control. Results from the *in vivo* imaging systems (IVIS) camera system indicated that the bioluminescence was on average 20 times lower on the PDEGMA titanium implant devices compared to the bare titanium control plates, with eight out of nine control plates showing distinct biofilm formation, while most of the PDEGMA plates showed no biofilm to be formed. SEM images of the implanted plates following seven days of implantation also confirmed significantly higher *S. aureus* adhesion to the control titanium plate surfaces, with clear surfaces observed on PDEGMA brush coated titanium plates. Therefore, the PDEGMA coated surface showed antifouling and antibacterial activity. However histological analysis did reveal the PDEGMA brush surface was unable to prevent bacterial infection in soft tissue located a few millimeters away. For future investigation authors recommended using a thicker coating of brush, or combining this with other methods such as covalently linking an antimicrobial to the polymer brushes.^533^

#### 6.1.2. Supramolecular hydrogel using peptide self-assembly

As previously discussed within this review, peptides can be used to form biomaterials, including hydrogels;^534^ peptidic hydrogels utilise the inherent self-assembling nature of peptides, and are rapidly being developed towards a myriad of applications in biomedicine.^535–537^ However, the peptide itself can also act as an antimicrobial agent in its single molecule form, either as the d- or l-amino acid, which can impart differing antimicrobial activities, as shown by Guo *et al.*^538^ The peptide backbone can also be used as a scaffold for nanoparticles,^539^ nanotubes^540^ and other constructs.^541,542^ Functionalising these biomacromolecules can imbue them with targeting properties; Liu *et al.* added a galactose moiety to their peptide hydrogel, targeting a carbohydrate-binding protein called lectin found on the surface of *P. aeruginosa*.^543^ Carbohydrate–lectin interactions have been shown to be involved in many biological processes, including cellular growth, differentiation, tumour metastasis and bacterial infection.^544–546^ These interactions are relatively weak as they are comprised of a combination of hydrogen bonds, electrostatic and hydrophobic interactions that can be overcome through multivalent displays of the carbohydrate ligand on the surface of the cell.^547–550^

In an attempt to understand the mechanisms involved in the formation of the competing carbohydrate–lectin interactions, scaffolds that mimic the glycocalyx structure (the pericellular matrix that surrounds the cell membranes of some bacteria) have been synthesised.^543,551–553^ These scaffolds have been seen to block infection and biofilm formation through competitive binding with the bacteria.^549,554^ To specifically orientate the carbohydrate ligands for optimum binding, supramolecular tools, specifically non-covalent interactions, including π–π stacking, hydrogen bonding and hydrophobic associations, were utilised.^555,556^

One carbohydrate–lectin interaction that has been studied extensively is the PA–IL galactose binding lectin, LecA, also found on the surface of *P. aeruginosa*. *P. aeruginosa* is commonly found in wounds and is involved in acute and chronic lung infections, a common complication of cystic fibrosis and a prevalent co-morbidity in cancer.^557–561^ PA–IL plays a role in bacterial virulence, cellular adhesion and invasion, and biofilm formation.^550,562^ Liu *et al.* targeted the PA–IL binding lectin using a supramolecular hydrogel to inhibit these activities of PA–IL, thus acting as an anti-infective agent.

**Fig. 35:**
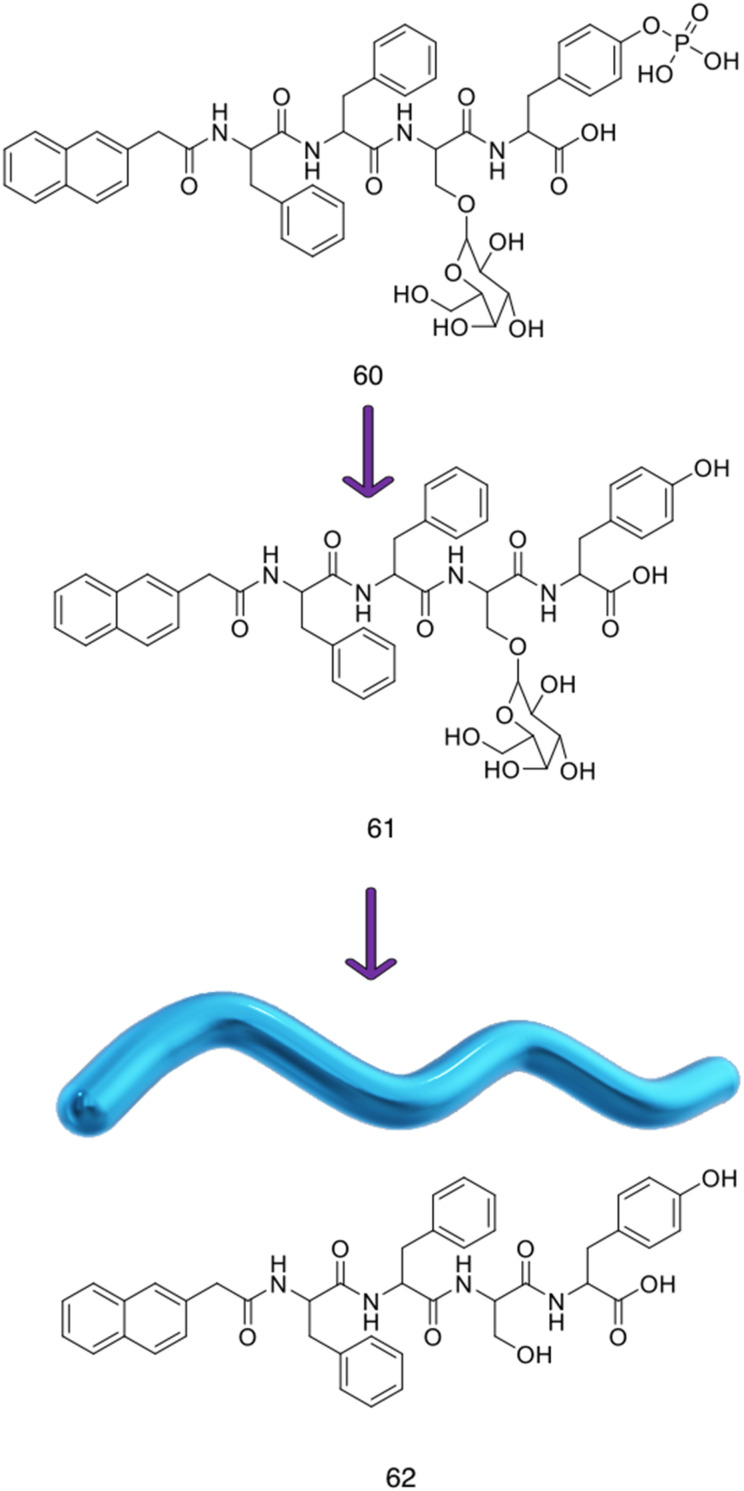
Chemical structures of compounds 60–62.^543^

Here, the authors created a peptidic scaffold with a terminal naphthyl group, a tetrapeptide segment (FFSY), H_2_PO_3_, a phosphate group to aid water solubility, and a sugar moiety linked to the side group of serine, in this case d-galactose, as shown in, producing compound 60 (Fig. 35). This peptidic scaffold was shown to self-assemble upon the enzymatic removal of the phosphate group by endogenous alkaline phosphatase (ALP) producing compound 61.^563,564^ The formation of hydrophobic and π–π interactions between the naphthyl and the two tyrosine moieties was hypothesised to confer rigidity to the self-assembled structure. The resulting nanofibers then self-assembled into a hydrogel.

The authors used a range of techniques to characterise the morphologies of compounds 60 and 61, using compound 62 as a control (which contains the same naphthyl tetrapeptide structure without the galactose moiety). The morphologies of compounds 60 and 61 before and after supramolecular self-assembly were investigated. SEM revealed the gel formed from 61 to have a fibrous/porous structure. Using CD, the authors found that compound 60 had no regular secondary structures in water, compared to the compound 61 gel which indicated β-sheets. FTIR was also used to confirm the presence of these β-sheets.

For this supramolecular hydrogel to display antimicrobial activity against *P. aeruginosa*, the galactose moieties must be accessible to the bacteria. The authors proved that this was the case by performing turbidity assays using peanut agglutinin (PNA), a plant protein that preferentially binds to galactose containing carbohydrates, and concanavalin (ConA), a plant protein that preferentially binds to mannose. In the presence of galactose, the compound 61 gel would agglutinate (antigen binding) with PNA, giving rise to a peak at 420 nm in the absorbance spectrum. Such a peak was observed when testing the compound 61 gel with PNA, indicating that the galactose moieties were freely available for PNA to bind to. A solution of 60 was also tested and yielded a negative result, which the authors attributed to the low binding affinity between a single 60 molecule and PNA. A concentration of compound 61 below its aggregation concentration was also tested, again yielding a negative result. These data indicated that self-assembly is necessary to form the multivalent clusters of galactose on the surface of the self-assembled structures. As expected, compound 62 molecules also did not trigger the agglutination of PNA as galactose was not present within the molecule. To ensure that the binding properties of 61 were specific, the authors performed the same assays against glucose/mannose specific lectin ConA,^565^ under the same environmental conditions. No absorbance was seen across all samples, indicating the specificity to galactose over mannose.

As previously stated, inhibition of the *P. aeruginosa* galactose-binding lectin PA–IL has been reported to prevent biofilm development.^566–568^ Diggle *et al.* showed that removal of the lecA gene *via* mutation, causing a reduction of PA–IL in the bacteria, reduced biofilm depth and coverage.^569^ This reduction in activity of PA–IL can also be achieved by outcompeting the ligands on the surface using multivalent galactose ligands.^570,571^ The authors examined the potential of the compound 61 gel to inhibit *P. aeruginosa* (ATCC 27853) in both planktonic and biofilm phenotypes. Bacteria were incubated with the compound 61 gel for 48 hours, then stained with crystal violet. Crystal violet binds to proteins and DNA,^572^ including the eDNA and polysaccharides found in the EPS.^573,574^ As this dye binds to both the matrix and the cells themselves, this assay can be used to evaluate total biofilm mass *via* fluoresence.^575^ The authors tested three experimental conditions and a bacteria control, the compound 62 gel, the compound 61 gel, and a combination of the 61 gel with the antimicrobial polymyxin B at 20 μg mL^−1^. The compound 62 gel showed no noticeable inhibitory effect against the biofilms, with fluorescence intensities similar to that of the control group. In comparison, the compound 61 gel caused a 43% inhibition in biofilm formation. Polymyxin B by itself decreased *P. aeruginosa* biofilm biomass by 55%, whilst the combination of 61 and PMB caused an 80% inhibition, indicating co-operative effects.

Unlike most conventional antibiotics, antibiofilm compounds are often anti-virulent; they do not affect bacterial growth, instead interfering with virulence factors and QS pathways.^576^ Virulence factors are required for a bacterium to cause disease *i.e.*, fimbrillae and clumping factors (to adhere to host cells), proteases (to hydrolyse proteins), lipases (to decompose lipids), specific toxins (*i.e.* pore forming toxins) and non-specific exotoxins (to disrupt cell signalling).^576–579^ Due to the hydrophobic nature of the compound 61 gel, the authors predicted it would have the ability to disrupt bacterial membranes.^580^*P. aeruginosa* was incubated on the surface of the compound 61 gel, the compound 62 gel and on the surface of the culture plates as the control, and after 24 hour incubation at 37 °C, bacterial viability was measured using colony counting. Similar to the biofilm results, the compound 62 gel showed very little inhibition (5%) whereas the compound 61 gels induced a 53% inhibition. The authors then performed a live/dead assay. Incubation of the bacteria with the compound 61 gel revealed high levels of dead bacteria adhered to the surface of the gel. The compound 62 gel showed high cell viability, similar results to that of the control.

To ensure that the antibacterial and antibiofilm activity of compound 61 was due to the specific multivalent galactose–PA–IL interaction, a series of bacterial growth tests were performed on *E. coli*, which do not exhibit PA–IL on the surface of the cells. *E. coli* (ATCC 25922) cultured on the surface of the compound 61 gel showed extremely high cell viability, and SEM showed that the bacteria grown on the gel had cell surfaces similar to those of the control group. These results were compared to *P. aeruginosa* cultured on the compound 61 gel. These bacteria displayed ‘wrinkled’ surfaces with broken and damaged membranes, indicating that it is the PA–IL interactions causing these effects. To understand the mode of action of the compound 61 gel, the authors carried out two cellular uptake assays. In brief, *P. aeruginosa* was washed with buffer, then re-suspended in ANS or DiSC_3_(5). ANS is a hydrophobic dye that can permeate membranes and DiSC_3_(5) is a voltage sensitive dye which accumulates on hyperpolarised membranes, causing quenching, followed by release upon antimicrobial induced depolarisation and subsequent flurescence.^581,582^ Bacterial suspensions were then added to the three samples and the fluorescence intensity was measured; as the membranes of the bacteria are disrupted, an increase in fluorescence is observed. The authors discovered that the cell death of *P. aeruginosa* was induced by depolarisation and permeation of both inner and outer membranes, whereas the compound 61 gel could only disrupt the outer membrane of *E. coli*. Finally, the authors tested the effect of the compound 61 gels on the cell viability of human umbilical vein endothelial cells (HUVECs). Over the course of 72 hours, the cells showed over 90% cell viability, indicating good biocompatibility.

#### 6.1.3. Chitosan–PEG–peptide conjugates

**Fig. 36:**
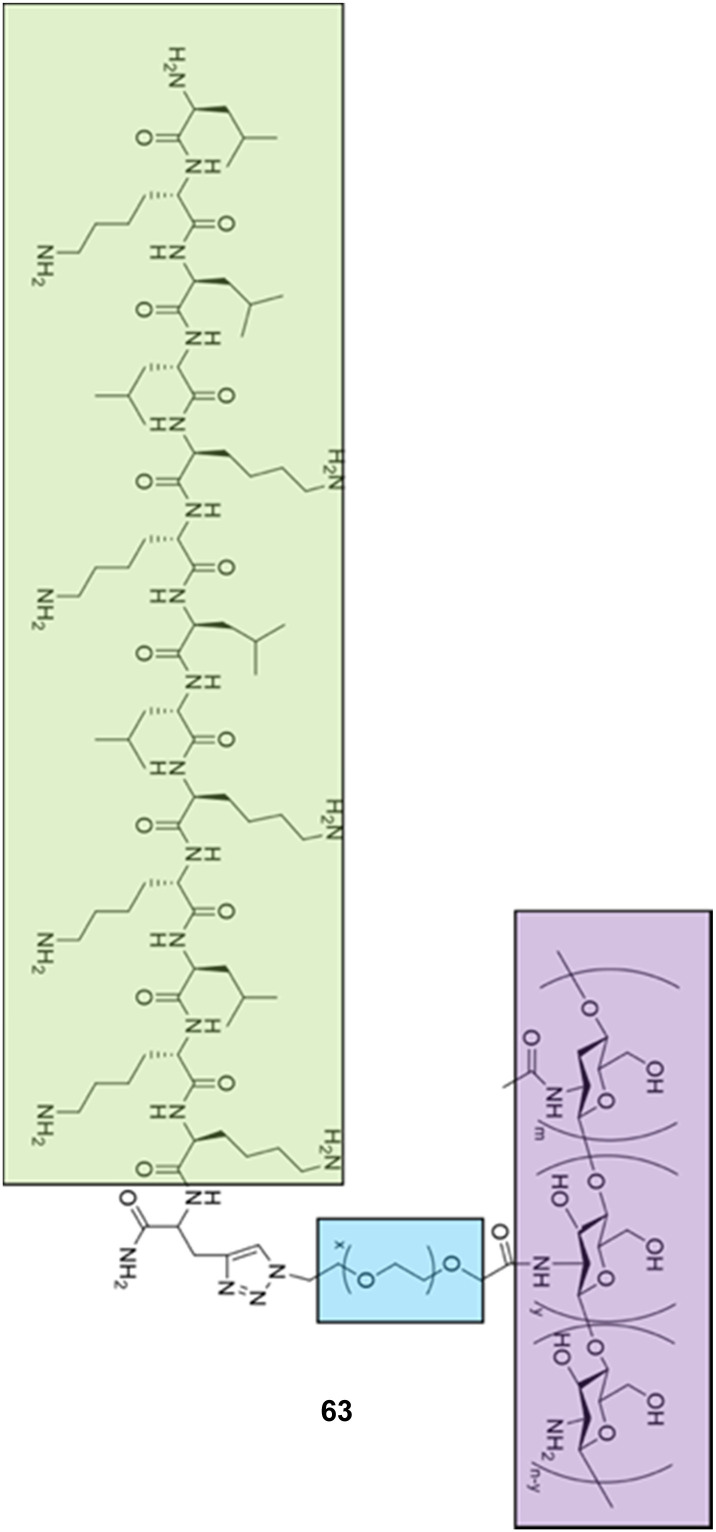
Chitosan–polyethylene glycol–LK_13_ peptide, 63, with chitosan outlined in green, polyethylene glycol in blue, and the LK_13_ peptide in purple.^587^

As previously discussed, biofilms contain channels and pores for nutrient and waste transportation between cells and their environment.^490,508,583^ These channels and pores range in size, from ≈10 nm to the micrometer scale, depending on function.^473,522,584,585^ The EPS matrix displays an overall anionic charge, due to its composition.^474,475^ Therefore, uncharged antimicrobial and antibiofilm agents are able to freely diffuse through the EPS.^475^ For example, the positively charged aminoglycoside antimicrobial tobramycin has little effect on *P. aeruginosa* biofilms, whereas the neutral antibiotic fluoroquinolone ciprofloxacin can penetrate easily.^586^ In an attempt to take advantage of these effects, Ju *et al.* synthesised antimicrobial peptides which self-assemble into neutral nanospheres.^587^ The peptide, 63, developed for this purpose was a chitosan–polyethylene glycol–peptide conjugate (LKLLKKLLKKLKK, LK_13_), as shown in Fig. 36.^587^ Compound 63 was shown to self-assemble into a neutral nanosphere in aqueous conditions, where the PEG moiety was presented on the surface of the structure while the LK_13_ peptide remained contained within the core of the structure. Upon interaction with bacterial membranes the nanosphere disassembles, enabling the antimicrobial action of the LK_13_ peptide of compound 63.^580,588–590^

Similar to Liu *et al.*, the authors used a combination of FTIR and CD to characterise compound 63.^543^ The authors also used a combination of TEM and DLS to determine the diameter of the nanospheres, which were found to be ≈100 nm. Zeta potential measurements were used to deduce the surface properties and charge of the nanospheres as 0 mV, compared to the LK_13_ peptide alone which exhibited a value of ≈+20 mV. The authors also measured the zeta potential and DLS at pH 5.5 to model the EPS, and compound 63 remained 0 mV and ≈100 nm in diameter. To investigate the secondary structure of compound 63, the authors used CD in a range of solutions. In a solution of sodium dodecyl sulfate (SDS), which due to its amphiphilic nature (hydrophobic head group and hydrophilic tail) can mimic a biological membrane,^591,592^ both the LK_13_ peptide and 63 were able to form α-helices. In pure water, both showed random conformations, mimicking results obtained with natural AMPs.^580,593^ The authors went on to investigate what secondary structures would be formed when compound 63 interacted with membranes of different compositions. Two bi-layers of synthetic phospholipids, phosphatidylglycerol (POPG) and phosphatidylcholine (DOPC) as general models for bacterial and mammalian membranes respectively were chosen.^115^ The different synthetic lipids were applied to compound 63 within the CD experiment. Compound 63 exhibited an α-helical secondary structure in the presence of POPG, but in DOPC it remained in a random conformation. The authors attributed the formation of these supramolecular structures to be due to two factors: (i) the electrostatic interactions between negatively charged POPG and positively charged LK_13_; and (ii) the hydrophobic interactions between LK_13_ and the phospholipids. These differences in secondary structure suggests that compound 63 disassembles upon interacting with the negatively charged bacterial membrane, allowing the LK_13_ to form an α-helix, which is essential for antimicrobial and antibiofilm activity. This indicates selectivity for anionic bacterial membranes over neutral mammalian cells.

To measure the antibacterial efficacy of compound 63 and the LK_13_ peptide, the authors determined the MIC against *P. aeruginosa* (ATCC 15442), *E. coli* (ATCC 25922) and *S. aureus* (ATCC 6538). The MIC values for the LK_13_ peptide and compound 63 determined against *P. aeruginosa* and *E. coli* were the same, at 8 and 16 μg mL^−1^ respectively, however against *S. aureus*, the LK_13_ peptide had an MIC of 16 μg mL^−1^, while compound 63 showed a decrease in activity to 64 μg mL^−1^. The authors attributed this decrease in activity to the outer membrane of Gram-negative bacteria which induced the disassembly of compound 63. However, Gram-positive bacteria, such as *S. aureus*, only have one bacterial membrane surrounded by peptidoglycan, thus exhibit a comparative decrease in cell surface charge density, leaving compound 63 assembled and the LK_13_ peptide inactive. To test the biocompatibility of the LK_13_ peptide and compound 63, a haemolytic assay was performed. The higher the value of the HC_10_ (amount of peptide/conjugate required to lyse 10% of the cells), the more biocompatible it is. The LK_13_ peptide had an HC_10_ of 13.1 mg mL^−1^ compared to compound 63 which had a HC_10_ of >32.0 mg mL^−1^, indicating enhanced biocompatibility.

To investigate the antibacterial mechanism of action, the authors investigated cell morphology using a combination of SEM, TEM and fluorescence spectroscopy. In the absence of compound 63, *P. aeruginosa* has a smooth cell surface, however after incubation with 63, the cells were observed to shrink and collapse, gaining a ‘wrinkled’ appearance with vesicle-like bulges. TEM indicated that the membrane had become permeable after incubation with compound 63. Two fluorescent dyes, ANS and PI, were used to further investigate these membrane disrupting effects. ANS binds to the hydrophobic regions of the membrane, with fluorescence enhancement trigger by damage to the outer membrane.^581^ PI is a dye that stains nucleic acids that cannot penetrate an intact membrane, thus intracellular PI indicates permeability.^594^

As a negative control, the authors used the aminoglycoside tobramycin in conjunction with ANS and PI in *P. aeruginosa* cells. As tobramycin does not target the bacterial membrane, there is no increase in the fluorescence response of ANS.^595^ Incubation of *P. aeruginosa* with 63 yielded an increase in the fluorescence intensity of ANS, demonstrating the ability of the compound 63 to disrupt the outer membrane, similar to the primary mechanism of action of AMPs.^580,588,589,596^ The increased fluorescence intensity of PI also indicated that compound 63 destroyed the bacterial membrane, thus allowing PI to interact with the intracellular nucleic acids. Unconjugated LK_13_ alone caused a slight increase in the fluorescence of both ANS and PI, but at a lower intensity for ANS and at a reduced rate for PI, when compared to 63, indicating that compound 63 was more effective than either the CS-PEG or the LK_13_ peptide. The authors attributed this phenomena to the effect of aggregation-enhanced antibacterial activity;^581,596,597^ the self-assembly of 63 into spheres results in a higher local concentration of LK_13_ at the membrane, which in turn causes greater disruption.

The authors proceeded to evaluate the antibiofilm activity of compound 63 on mature biofilms of *P. aeruginosa*. The mature biofilms were incubated with 63, LK_13_ and tobramycin at 8 times the MIC for 24 hours, and the number of CFU was calculated. The LK_13_ peptide induced a 15% and the tobramycin a 34% decrease in CFU respectively, while 63 induced a 73% growth inhibition. The authors attributed this significant difference between the inhibitory action of LK_13_ and 63 to the ability of compound 63 neutral nanospheres to travel through the EPS. Increasing the dosage to 12.5× the MIC enabled 63 to reach 98% inhibition. A live/dead assay was performed at this concentration and showed that compound 63 could penetrate throughout the entire biofilm.

Finally, the authors assessed compound 63, LK_13_ and a positive control of tobramycin (1562 times the MIC) *in vivo via* implanted *P. aeruginosa* biofilms in an albino mouse wound model. The mice were injected at the biofilm site with compound 63, LK_13_ or tobramycin, all at 6.7 mg kg^−1^, with a vehicle control of PBS 24 hours after biofilm implantation. After 72 hours, mice treated with compound 63 or excess tobramycin all showed well healed wounds, with no visible inflammation. LK_13_ injected mice wounds were unhealed, but there were no visible signs of inflammation. The mice exposed to the vehicle only control all showed visible inflammation, with unhealed and festering wounds. The implanted biofilm was removed, and the cell viability was calculated. LK_13_ at 12.5 times the MIC reduced cell viability to 25% whereas compound 63 and excess tobramycin decreased cell viability to near zero. The authors also used histological staining and examined the expression of necrosis factors to visualise the infection, with similar results to the wounds: compound 63 and excess tobramycin showed almost no signs of infection, LK_13_ displayed increased infection levels, whilst the control group showed the greatest evidence of infection. The side effects of the treatments were also evaluated by haematoxylin and eosin staining. In this experiment, neutrophils are stained blue, and in this case an increased presence of neutrophils is indicative of infection. Treatment with compound 63 displayed fewer neutrophils than the vehicle only control group. Liver cells in the excess tobramycin group were seen to be disordered, which the authors attributed to the hepatotoxic effects of tobramycin.^598,599^

#### 6.1.4. Curcumin modified micelles

NPs are another avenue currently being investigated as probes and drug nanocarriers against biofilms, as they have a well-documented ability to penetrate the EPS.^600–602^ The chemical composition, surface topography and size of NPs can be tailored and designed towards increased antibiofilm activity.^603^ Commonly used metallic NPs (gold, silver, copper, iron and zinc) offer poor biocompatibility and an undesirable environmental fate.^604,605^ Polymeric NPs however, offer an alternative with increased biocompatibility and are often biodegradable.^606^ Polymeric micelles are particularly attractive as they are readily synthesised using amphiphilic co-polymers, offer tuneable properties, are easy to prepare and low cost,^607^ thus they find use in many biological applications.^608^ These micelles can encapsulate bioactive molecules, including water-insoluble compounds, and deliver them into biofilms.^609,610^ The delivery capability of copolymer micelle is often termed their ‘stealth abilities’; non-specific binding between the encapsulated cargo and the EPS is prevented, as the groups on the surface of the micelle do no interact with the EPS.^611,612^ The delivery can then be triggered by external stimuli, *i.e.* pH,^613^ enzymes^614^ or light.^615^

To increase the efficacy of this delivery strategy, Barros *et al.* suggested imbuing the micelle itself with antimicrobial activity. By covalently binding a bioactive compound to the polymeric backbone, antimicrobial activity would be conferred to the micelle.^616,617^ Li *et al.* achieved this by including triclosan and biguanide groups into the backbone of a NP, giving it inherent activity against MRSA.^617^ To prevent the need for covalent modification of the polymeric backbone, antimicrobial activity can also be achieved by adding antimicrobial moieties to the outside of the micelles.^618^ Inherently antimicrobial micelles already used as delivery vehicles include chitosan-PLGA and Soluplus® micelles.^619^

**Fig. 37:**
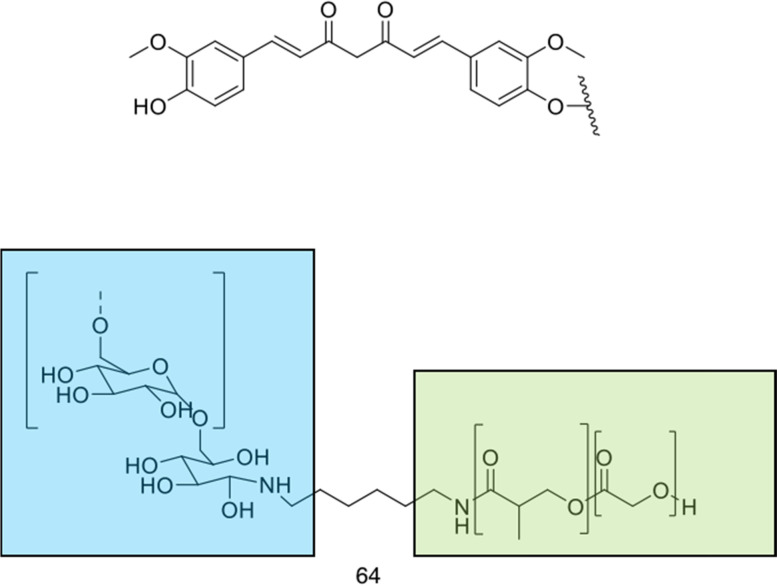
(top) Curcumin and its attachment point to the poly(lactic-*co*-glycolic acid)-dextran_10_) (PLGA-Dex_10_) copolymer. (bottom) Compound 64 (PLGA-Dex_10_) copolymer synthesised by Barros *et al.*^620^ Compound 65 is 64 attached to curcumin.

Barros *et al.* used poly(lactic-*co*-glycolic acid) (PLGA) as the hydrophobic component of a micelle due to its biocompatibility, low cost, and ease of chemical modification.^620,621^ Dextran was chosen as the hydrophilic shell for similar reasons; it can be functionalised easily, is biodegradable in nature and is compatible with the EPS matrix,^622,623^ (Fig. 37). Curcumin, a natural product derived from *Curcuma longa* (turmeric),^624^ (Fig. 37), was linked to the PLGA-dextran co-polymer. Although curcumin is not yet approved for medical use, curcumin has well established biological activities including antiinflammatory,^625^ anticancer^626^ and antibacterial properties.^627^

The copolymer 64 (PLGA-Dex_10_) was synthesised and characterised using FTIR and UV-Vis. The spontaneously self-assembled micelles were shown to have a hydrodynamic diameter of ∼100 nm using DLS measurements, and a zeta potential of −6 mV. Despite the weak electrostatic stabilisation of the zeta potential, the authors noted that the dispersions were stable for months, attributed to steric stabilisation. These micelles were also visualised by SEM, although the sizes were larger than expected, hypothesised to be due to Ostwald ripening. This is a process in which small particles deposit or combine with larger structures to reach a more thermodynamically stable state.^628^ Compound 64 was then functionalised with curcumin (PLGA-Dex_10_-cur), producing compound 65 through an EDC/NHS coupling, resulting in a 5-fold increase in hydrodynamic diameter (≈500 nm) and zeta potential readings of +26 mV. The higher zeta potential readings were attributed to residual *N*-(3-dimethylaminopropyl)-*N*′-ethylcarbodiimide hydrochloride (EDC) which was used in excess to form the functionalised micelles and was still present after repeated dialysis and centrifugal washes. The micelles were also viewed using SEM, with compound 65 micelles shown to have a rough surface, compared to the smooth surface of unfunctionalised micelles.

The authors confirmed the presence of curcumin in compound 65*via* the observation of an absorbance band at 425 nm in the UV-Vis spectrum and calculated that 65 contained 1.8% w/w curcumin loading. Compound 65 was also characterised using FTIR and CMC. The functionalisation of 64 with curcumin increased the CMC 2-fold, from 620 to 1240 μg mL^−1^. This was attributed to the hydrophobicity of curcumin; its inclusion reduces the difference in hydrophilicity between the dextran shell and PGLA core, and thus reduces the amphiphilic nature of the micelle. The increase in CMC was postulated to be caused by more co-polymer units needed to form a micelle due to the curcumin.

The antibacterial properties of both compound 64 and 65 micelles and free curcumin (at equivalent concentrations to the micelles) were investigated against *Pseudomonas putida* (*P. putida*) (PCL 1482) and *P. fluorescens*) (PCL 1701). Interestingly, compound 64 had an antimicrobial effect against *P. fluorescens*, but not *P. putida*. The authors attributed this activity to the dextran moiety, as dextran has been previously observed to exhibit antibacterial and antibiofilm effects against some *Pseudomonas* strains but not others, due to differences in the phenotype and genotype.^629,630^ Against *P. putida*, compound 64 slowed down the growth rate of the planktonic cells. However, compound 65 was shown to have antimicrobial activity against both strains in a concentration dependent manner. Free curcumin displayed no inhibitory activity against either strain, hypothesised to be due to the insolubility of curcumin; 65 may thus act as either a transporter or solubilising agent for the curcumin, enabling activity. There are many theories as to the mechanism of the antibacterial activity of curcumin, including the induction of membrane damage and leakage of intracellular components.^631^ Changes in gene expression is one proposed mechanism in the case of *Dictyostelium discoideum*^632^ and *P. aeruginosa*,^465^ however no unifying mechanism of action has been determined.^633^

The biofilm-inhibition properties of compounds 65, 64 and free curcumin were elucidated by estimating the biomass of the biofilms after a 24 hour incubation with *P. fluorescens* and *P. putida* using crystal violet staining. All showed anti-biofilm activity compared to the control, but no significant trend could be seen with respect to either the concentration or formation of micelles. The authors attributed the lack of trend to the micelles inability to inhibit bacterial adhesion. Next, the ability of the micelles and free curcumin to disrupt pre-formed biofilms was evaluated against both bacteria. Disruption was observed with both micelles and free curcumin, although 65 caused a greater decrease (46%) in biomass at lower concentrations, 0.625 mg mL^−1^, compared to 64 (33%) against *P. fluorescens*. The ability of compound 65 micelles to disrupt biofilms was attributed to its negatively charged phenolates and the interaction between curcumin and the EPS. This second mechanism of action was deemed to be more likely, as *in silico* studies show that free curcumin can disrupt biofilms.^634^

To study the cell viability of the biofilms, the authors used the MTT assay. In the case of *P. fluorescens*, compound 64 was able to significantly reduce cell viability at 1.25 mL^−1^, a result also seen against the planktonic cells. Compound 64 did not have any effect on *P. putida* biofilms, mirroring the planktonic cell results. Compound 65 showed a significant reduction in cell viability in all concentrations tested against *P. putida* but did not outperform free curcumin. This data is very different from the other antibiofilm assays, which the authors attributed to the other experiments not distinguishing between live and dead bacteria, indicating that inhibition of the EPS does not necessarily correlate to dead bacteria.

To visualise how the micelles interact with the biofilm, the authors labelled 64 with two fluorophores: fluorescein, for imaging mCherry-expressing *P. fluorescens*, and rhodamine B, for imaging green fluorescent protein (GFP)-expressing *P. putida*. These cell lines naturally express fluorescent proteins, allowing the bacteria within the biofilm to be imaged using fluorescence microscopy. The functionalised 65 could not be imaged due to the inherently fluorescent nature of curcumin, which features a wide excitation and emission range (≈460–600 nm) which overlap both GFP and mCherry excitation and emission ranges.^635^ Compound 64 micelles were unable to penetrate deep into the *P. fluorescens* biofilms, as evidenced by the micelles not being visible within the densely packed regions of the biofilm. This contradicts the intrinsic antimicrobial activity previously reported, in which the authors attributed the reduction in biomass produced by *P. fluorescens* biofilms. Conversely, 64 could be seen in the denser regions of *P. putida* biofilm, indicating its ability to penetrate more readily through biofilms of this bacteria.

## 7. Conclusions

After analysis of the self-assembling based therapies covered; self-assembling peptides, self-assembling small molecules and self-assembling macromolecules, a common mechanism by which each achieves antimicrobial activity is evident. Membrane targeting and subsequent permeabilization can be achieved through assembly into macrostructures, which results in bacterial lysis. All three classes of self-assembling therapies utilise the inherent difficulties for microbes to develop resistance to membrane targeting antimicrobials^147^ offering potentially useful tools in the global struggle against AMR. Comparing across the three self-assembling building block types, specific advantages and trade-offs are incurred, and thus researchers must consider which qualities they wish to incorporate into the foundation of their system. Specifically, peptides provide a catalogue of naturally derived molecules evolved for antimicrobial purposes, providing clear starting structures for development, however this is counterbalanced by their stability and haemolysis issues; small molecules are backed by a wealth of PK and PD data, theoretically facilitating a faster route towards clinical application, whilst in doing so trade off complexity that could reduce efficacy; macromolecules allow for significant tailorability and instillation of complexity, but data is reduced/less favourable^256,636^ compared to small molecules on important parameters required for clinical translation including that of PK and PD data^637^ (*e.g.* not available for oral administration^255^), as a result of the increased complexity and diverse array of potential structures. Within each system, researchers demonstrated the ability to reach MICs comparable to commonly used traditional antibiotics, demonstrating the efficacy of these approaches, with steps towards animal studies already being undertaken in several examples yielding promising results. Of the self-assembling systems discussed, self-assembling peptides present the most probable system for prompt translation into the clinic. Systems of this nature have already participated in and demonstrated favourable results for clinical trials in wound healing,^638^ allowing one to imagine a near future with self-assembling peptides prescribed as antimicrobial treatments.

While the self-assembling systems covered produced a diverse array of nanostructures and aggregate materials, NPs, NPMs and nanoscale delivery vehicles were discussed as a separate category due to their distinct mechanisms of action. NPs, with their high aspect ratio and ROS inducing capabilities demonstrate potent MICs against a variety of microbes. Whilst this is promising preliminary data, the field of NPs and their use in medicine is still in its infancy, and requires further investigation for performance in humans, specifically with regard to dosing and clearance.^359^ In addition, AMR development against NPs has already been demonstrated in rare instances for several NP systems,^639^ indicating susceptibility in the future. Despite these challenges, hope can be gained from the clinical trials launched investigating NPs for applications including medical imaging and cancer treatments, with some NP systems having received FDA approval.^640,641^ With the increase in data for NP performance in humans, the feasibility of NP based antimicrobials entering clinical trials in the very near future is promising.

NPMs focus on the specific application of medical implant antifouling. Such materials are vital to ensure that AMR does not hinder the progress achieved in medical implant technology, reducing complications of diseases including that of cardiovascular disease (CVD), the current leading cause of death in western society.^642^ Currently five clinical trials are being undertaken on medical implant devices containing nanopatterned materials,^643^ highlighting the very real future for clinical application. The nanoscale delivery vehicles discussed within this review all aimed to enhance the efficacy of currently available antibiotics. With good levels of selectivity observed and improved MICs compared to the antibiotic alone, nanoscale delivery vehicles have been established as likely candidates for the future of antimicrobial treatments. However, even with these favourable performances in pre-clinical studies, nanoscale delivery vehicles have been plagued with multiple issues, specifically those of intellectual property, scalability, reproducibility, administration route in addition to poor biocompatibility.^644^ These fundamental roadblocks will need to be addressed to enable line-of-site into the clinic. As a consequence, we hypothesise that NPs and NPMs are the most likely immediate solution to AMR in the nanomaterial area.

Finally, we addressed the application of these therapy types towards biofilm eradication, a key focus within AMR research. Each of the systems discussed demonstrated successes in eradicating biofilms, with one study achieving favourable results in a murine model, demonstrating a significant step towards success in pre-clinical studies. With further enhanced understanding of the aforementioned systems against planktonic microbes, increased application may be observed towards biofilms.

It is imperative with all drug development that control experiments look to determine cytotoxicity against all relevant human cells, in addition to the drugs antimicrobial activity. In the case of antimicrobial peptides, often only haemolysis assays are reported.^645,646^ However, whilst this represents a major source of AMP toxicity, more thorough investigation into other cell lines using established methods (such as the MTT assays) should become the standard when investigating self-assembling and nanomaterials. Furthermore, when considering both antimicrobial activity and cytotoxicity, it can be difficult to deconvolute the cause of the observed effects. As with traditional antimicrobial therapies, it is possible to measure a MIC with a self-assembling molecule before it reaches the concentration at which it assembles. However, unless there is significant activity before the point of self-assembly it is difficult to differentiate if the observed effects are due to increased concentration or as a consequence of the self-assembly process.

Though this review does not focus on the development of classical antimicrobial therapies, it is worth discussing the attempts at de-risking small molecule antibiotic development as an investment, an effort being made by governments and philanthropic organisations. The so-called “product development partnership” model was introduced in the 1990's in order to accelerate drug development for neglected diseases,^647^ and those which primarily affect impoverished regions which do not yield returns on investment. The success of this model in the development of treatments for malaria by the Medicines for Malaria Venture is exemplified by the 11 new medicines it has translated into clinical practice since 1999.^647^ The Global Antibiotic Research and Development Partnership (GADPR), established in 2016, seeks to mirror this success and bring five new successful antimicrobial treatments into the clinic by 2025.^648^ Based in Switzerland but funded by a range of international governments and private institutions, the GADPR was set up as a collaboration between the WHO and the Neglected Disease Initiative, focusing on the treatment of childhood infection, sexually transmitted infections and sepsis, so far raising € 104.7 million in funding to date.^649^ Crucially, as well as helping to drive the development of these new therapies, the GADPR takes an active role in the stewardship of these treatments. Antimicrobial stewardship is designed to help maintain efficacy of treatments for as long as possible through carefully restricted usage; the importance of this topic and resulting implementations is extensively discussed in a review by Hooton and co-workers.^650^

It is clear from all the therapies discussed, including self-assembled systems and nanoscale materials, similar barriers of an unclear pipeline towards a successful final product are present, hindering potential progression to the clinic. Such issues can be expected due to the separation between the academics pursuing these technologies and the large industrial companies able to progress the therapy towards clinical trials.^651^ A review by Metseaar and Lammers suggested a larger focus for academics pursuing nanomedicine development should be directed towards the therapeutic endpoints, with consideration of preclinical setup, formulation specifications and manufacturing methods from the outset, to enable smoother transition into the clinical setting.^652^

Given the wealth of technological innovations undergoing development within the fields of antimicrobial nanoscale materials and self-assembling systems, it is important for scientists hoping to move towards clinical application to carefully consider the later stage barriers that tend to hinder these therapies entrance into the clinic. We hope that by discussing the technologies within this review, scientists can select the best starting point to develop their technology, and develop an understanding of streamlined processes that can accelerate the translation of the fundamental research.

## Abbreviations

*A. baumannii*
: *Acinetobacter baumannii*
ADMA: 2,2′-(Anthracene-9,10-diylbis(methylene))dimalonic acid
ADME: Absorption, distribution, metabolism and excretion
AFM: Atomic force microscopy
ALP: Alkaline phosphatase
AMP: Antimicrobial peptides
AMR: Antimicrobial resistance
ANS: 8-Anilino-1-naphthalenesulfonic acid
ANTS: 8-Aminonaphthalene-1,3,6-trisulfonic acid disodium salt
AO/PI: Acridine orange/propidium iodide
ATR: Attenuated total reflectance
*B. cereus*
: *Bacillus cereus*
*B. subtilis*
: *Bacillus subtilis*
BC: BODIPY TR cadaverine
bSi: Black silicon
*C. albicans*
: *Candida albicans*
*C. galbrata*
: *Candida galbrata*
*C. parapsilosis*
: *Candida parapsilosis*
CD: Circular dichroism
cFDA-SE: Carboxyfluorescein diacetate succinimidyl ester
CFU: Colony forming units
*c*log*P*: Calculated log*P*
CLSM: Confocal laser scanning microscopy
CMC: Critical micelle concentration
CMC: Critical aggregation concentrations
ConA: Concanavalin
CONS: Coagulase negative *staphylococcus*
CTAB: Cetrimonium bromide
CVD: Cardiovascular disease
DBCz-BT: (4,7-Dibromo-5, 6-di(9*H*-carbazol-9-yl)benzo[*c*][1,2,5] thiadiazole
DEPs: Differentially expressed proteins
DiSC_3_(5): 3,3′-Dipropylthiadicarbocyanine iodide
DLS: Dynamic light scattering
DOPC: Dioleoyl phosphatidylcholine
DPMC: Dimyristoyl phosphatidylcholine
DPPE: Dipalmitoyl-phosphatidylethanolamine
DPPG: Dipalmitoyl-phosphatidylglycerol
DPX: *p*-Xylene-bis-pyridinium bromide
DSC: Differential scanning calorimetry
DSPE-PEG: 1,2-Distearoyl-*sn-glycero*-3-phosphoethanolamine polyethylene glycol
*E. coli*
: *Escherichia coli*
EDC: *N*-(3-Dimethylaminopropyl)-*N*′-ethylcarbodiimide hydrochloride
eDNA: Extracellular DNA
EPS: Extracellular polymeric substance
ESI: Electrospray ionisation
FTIR: Fourier transform infrared spectroscopy
GADPR: Global Antibiotic Research and Development Partnership
GFP: Green fluorescent protein
HDF: Human dermal fibroblasts
HGT: Horizontal gene transfer
HUVECs: Human umbilical vein endothelial cells
ITC: Isothermal titration calorimetry
IVIS: *In vivo* imaging systems
*K. aerogene*
: *Klebsiella aerogenes*
*K. pneumoniae*
: *Klebsiella pneumoniae*
*L. monocytogenes*
: *Listeria monocytogenes*
LCST: Lower critical solution temperature
LPS: Lipopolysaccharide
*M. luteus*
: *Micrococcus luteus*
*M. phlei*
: *Mycobacterium phlei*
*M. smegmatis*
: *Mycobacterium smegmatis*
MBC: Minimum bactericidal concentration
MBIC: Minimum biofilm inhibition concentration
MDR: Multi-drug resistant
MGC: Minimum gelation concentration
MIC: Minimum inhibition concentration
MRSA: Methicillin resistant *Staphylococcus aureus*
MS: Mass spectrometry
MTT: Methylthiazolydiphenyltetrazolium bromide
NDI: Naphthalene diimide
NPM: Nanopatterned materials
NPN: *N*-Phenyl-1-naphthylamine
*P. aeruginosa*
: *Pseudomonas aeruginosa*
*P. fluorescens*
: *Pseudomonas fluorescens*
*P. putida*
: *Pseudomonas putida*
*P. syringae*
: *Pseudomonas syringae*
PAMAM: Poly(aryl ether) dendron-polyamidoamine
PBAE-G-B: Poly(β-amino ester)-guanidine-phenylboronic acid
PBP2A: Penicillin binding protein 2a
PBS: Phosphate buffered saline
PC: Phosphatidylcholine
PD: Pharmacodynamic
PDEGMA: Poly(di(ethylene glycol)methyl ether methacrylate)
PDI: Polydispersity index
PE: Phosphatidylethanolamine
PG: Phosphatidyl glycerol
PI: Propidium iodide
PK: Pharmacokinetic
PLGA: Poly(lactic-*co*-glycolic acid)
PLL: Poly-l-lysine
PNA: Peanut agglutinin
POPG: Synthetic phosphatidylglycerol
PS-*b*-P2VP: Polystyrene-*block*-poly(2-vinylpyridine)
QS: Quorum sensing
*R. capsulatus*
: *Rhodobacter capsulatus*
*R. radiobacter*
: *Rhizobium radiobacter*
RBC: Red blood cell
ROS: Reactive oxygen species
*S. aureus*
: *Staphylococcus aureus*
*S. enteritidis*
: *Salmonella enteritidis*
*S. epidermidis*
: *Staphylococcus epidermidis*
*S. lutea*
: *Micrococcus lutea*
*S. pneumonia*
: *Streptococcus pneumonia*
*S. typhimurium*
: *Salmonella typhimurium*
SDS: Sodium dodecyl sulfate
SEM: Scanning electron microscopy
SLP: Surfactant-like peptides
SME: Small/medium sized enterprises
SSA: Self-associating amphiphilic salts
STM: Scanning tunnelling microscopy
TB: Tuberculosis
TBA: Tetrabutylammonium
TCPS: Tissue culture polystyrene
TEM: Transmission electron microscopy (p10)
*T*_m_
: Melting temperature
TPY: Multitpic terpyridine
TSP: Thrombin sensitive peptide
TWIM-MS: Travelling wave ion mobility-mass spectrometry
WHO: World Health Organisation

## Author contributions

Conceptualization (JAD, GTW); data curation and formal analysis (JAD, GTW, KLFH, RC); funding acquisition (JSF, BTG, JRH); project administration (GTW); supervision (JSF, GTW, BTG, JRH); validation (JAD, GTW, KLFH, RC, JSF, BTG, JRH); writing – original draft (JAD, GTW, KLFH, RC); writing – review & editing (JAD, GTW, KLFH, JSF, BTG, JRH).

## Conflicts of interest

There are no conflicts to declare.

## Supplementary Material
